# Angiotensin II-Regulated Autophagy Is Required for Vascular Smooth Muscle Cell Hypertrophy

**DOI:** 10.3389/fphar.2018.01553

**Published:** 2019-02-05

**Authors:** David Mondaca-Ruff, Jaime A. Riquelme, Clara Quiroga, Ignacio Norambuena-Soto, Fernanda Sanhueza-Olivares, Paulina Villar-Fincheira, Tomás Hernández-Díaz, Nicole Cancino-Arenas, Alejandra San Martin, Lorena García, Sergio Lavandero, Mario Chiong

**Affiliations:** ^1^Advanced Center for Chronic Diseases (ACCDiS), Centro de Estudios en Ejercicio, Metabolismo y Cáncer (CEMC), Departamento Bioquímica y Biología Molecular, Facultad Ciencias Químicas y Farmacéuticas, Universidad de Chile, Santiago, Chile; ^2^Advanced Center for Chronic Diseases (ACCDiS), División de Enfermedades Cardiovasculares, Facultad de Medicina, Pontificia Universidad Católica de Chile, Santiago, Chile; ^3^Division of Cardiology, Department of Medicine, Emory University, Atlanta, GA, United States; ^4^Department of Internal Medicine (Cardiology Division), University of Texas Southwestern Medical Center, Dallas, TX, United States

**Keywords:** angiotensin II, VSMC, autophagy, hypertrophy, AT1R, ROCK, losartan

## Abstract

Hypertension is a disease associated to increased plasma levels of angiotensin II (Ang II). Ang II can regulate proliferation, migration, ROS production and hypertrophy of vascular smooth muscle cells (VSMCs). However, the mechanisms by which Ang II can affect VSMCs remain to be fully elucidated. In this context, autophagy, a process involved in self-digestion of proteins and organelles, has been described to regulate vascular remodeling. Therefore, we sought to investigate if Ang II regulates VSMC hypertrophy through an autophagy-dependent mechanism. To test this, we stimulated A7r5 cell line and primary rat aortic smooth muscle cells with Ang II 100 nM and measured autophagic markers at 24 h by Western blot. Autophagosomes were quantified by visualizing fluorescently labeled LC3 using confocal microscopy. The results showed that treatment with Ang II increases Beclin-1, Vps34, Atg-12–Atg5, Atg4 and Atg7 protein levels, Beclin-1 phosphorylation, as well as the number of autophagic vesicles, suggesting that this peptide induces autophagy by activating phagophore initiation and elongation. These findings were confirmed by the assessment of autophagic flux by co-administering Ang II together with chloroquine (30 μM). Pharmacological antagonism of the angiotensin type 1 receptor (AT1R) with losartan and RhoA/Rho Kinase inhibition prevented Ang II-induced autophagy. Moreover, Ang II-induced A7r5 hypertrophy, evaluated by α-SMA expression and cell size, was prevented upon autophagy inhibition. Taking together, our results suggest that the induction of autophagy by an AT1R/RhoA/Rho Kinase-dependent mechanism contributes to Ang II-induced hypertrophy in VSMC.

## Introduction

Cardiovascular diseases are a major cause of mortality around the world (Roth et al., 2017). Hypertension, which is generally characterized by high angiotensin II (Ang II) plasma levels, is a highly prevalent vascular disease, given that by 2015, 874 million people were reported to have a systolic blood pressure of 140 mm Hg or higher (Forouzanfar et al., 2017). Vascular smooth muscle cells (VSMCs), a major component of the vessel wall, are responsible for the control of blood flow and arterial pressure by regulating the lumen’s diameter of resistance vessels in response to Ang II (Rzucidlo et al., 2007; Gomez and Owens, 2012) through the activation of the AT1 receptor (AT1R) (Mehta and Griendling, 2007). In addition to this physiological response, sustain activation of the AT1R results in vascular remodeling that permanently increased vascular resistance (Taubman, 2003).

It has been well-established that Ang II induces VSMC hypertrophy, characterized by an increase in cell size. α-smooth muscle actin (α-SMA) and protein synthesis (Geisterfer et al., 1988; Andrawis et al., 1993). Moreover, the size of VSMCs of hypertensive patients is increased in comparison to normotensive ones (Owens, 1985; Gariepy et al., 1993). Besides VSMC hypertrophy and contraction, Ang II-mediated AT1R activation can also regulate cell proliferation, migration and reactive oxygen species production (Rosendorff, 1996; Touyz, 2005). Many of these processes are mediated by the activation of the RhoA/Rho Kinase (ROCK) signaling pathway (Seko et al., 2003). Pursuing new mechanisms involved in vascular remodeling, autophagy has been described to regulate VSMCs phenotype (Jia et al., 2006; Salabei and Hill, 2013). Autophagy, is a tightly regulated protein degradation mechanism that begins with the formation of a phagophore, then a double membrane vesicle called the autophagosome that fuses with the lysosome to promote the degradation and recycling of proteins and organelles (Gatica et al., 2015; Lavandero et al., 2015). Autophagy is a multistep process comprehended by an initiation complex conformed by Beclin 1 and Vps34 and two ubiquitin-like system formed by LC3 and the AuTophaGy-related (ATG) proteins. Beclin-1 induces autophagy by forming a complex with Vps34, a phosphatidyl inositol-3-kinase type III (PI3KCIII) that generates phosphatidylinositol-3-phosphate [PtdIns (3) P], which is necessary for the elongation of the autophagosome (Yin et al., 2016). Following the induction process, elongation is achieved by the activation of two parallel pathways: ATG proteins and the LC3 protein system (Yang and Klionsky, 2010). In this pathway, Atg7 presents activity similar to the E1 enzyme of the ubiquitin-proteasome system, which mediates the conjugation of Atg12 to Atg5, forming the Atg12-Atg5 complex, which subsequently is coupled with Atg16 forming the complex Atg12-Atg5-Atg16, which is required for the elongation of the autophagosome. In the other pathway, Atg4 can proteolyze the pro-LC3 to produce LC3 I. Then, LC3 I is conjugated to phosphatidyl ethanolamine (PE) by the action of Atg7 and Atg3 (ubiquitin-like E2 enzyme), generating LC3 II, which binds to the autophagosome membrane favoring its elongation (Mizushima et al., 1998). The final step is carried out by the fusion of the autophagosome with the lysosome, forming an autolysosome. The degradation of the content located within the autolysosome is carried out by lysosomal hydrolases, and the catabolized products are released into the cytosol and recycled to cover the nutrient and/or structural needs (Gatica et al., 2015; Lavandero et al., 2015). In line with this, it has been reported that TNF-α (Jia et al., 2006) and PDGF-BB (Salabei et al., 2013), which are increased in vascular pathologies, may also induce autophagy and dedifferentiation of VSMCs (Chen et al., 2013; Salabei et al., 2013). Nonetheless, the association between autophagy and phenotype switching of VSMCs, remains to be fully elucidated. Therefore, we sought to investigate whether Ang II induces VSMC hypertrophy through an autophagy dependent mechanism.

Here, we show that Ang II induces autophagy through the activation of phagophore initiation and elongation by an AT1R/RhoA/Rho Kinase-dependent mechanism. Moreover, autophagy inhibition completely prevents Ang II-induced hypertrophy in VSMC.

## Materials and Methods

### Materials

Ang II (100 nM), losartan (1 μM), Y-27632 (10 μM), chloroquine (30 μM), siRNA scramble and siRNA against Beclin-1 were purchased from Sigma (Sigma-Aldrich, Corp., St. Louis, MO, United States). Spautin 1 (10 μM) was purchased from Cayman (Cayman Chemical Company, Ann Arbor, MI, United States). The following antibodies were used for the Western blot experiments: LC3 1:1000 (cat #2775 Cell Signaling, Danvers, MA, United States), Atg7 1:1000 (cat #8558 Cell Signaling, Danvers, MA, United States), Atg12–Atg5 1:500 (cat #2630 Cell Signaling, Danvers, MA, United States), Atg4 1:1000 (cat # 5299 Cell Signaling, Danvers, MA, United States), Beclin1 1:2000 (cat #3738 Cell Signaling, Danvers, MA, United States), p-Beclin1 Thr^119^ 1:500 (cat #ABC118 EMD Millipore, Darmstadt, Germany), Vps34/phosphatidylinositol 3-kinase class III (Vps34) 1:1000 (cat # 3811 Cell Signaling, Danvers, MA, United States), p-mTOR Ser^2448^ 1:1000 (cat # 2971 Cell Signaling, Danvers, MA, United States), p-TSC2 Thr^1462^ 1:1000 (cat # 3617 Cell Signaling, Danvers, MA, United States), p-p70s6k Thr^389^ 1:1000 (cat # 9205 Cell Signaling, Danvers, MA, United States), p-AMPKα Thr^172^ 1:1000 (cat # 50081 Cell Signaling, Danvers, MA, United States), p-4E-BP1 Thr^37/46^ 1:1000 (cat # 2855 Cell Signaling, Danvers, MA, United States), MYPT1 (cat # 2634 Cell Signaling, Danvers, MA, United States), p-MYPT1 Thr^853^ 1:1000 (cat # 4563 Cell Signaling, Danvers, MA, United States), α-SMA 1:20000 (cat #ab7817 Abcam, Cambridge, MA, United States), calponin 1:5000 (cat # ab46794 Abcam, Cambridge, MA, United States), SM22 1:5000 (cat #ab14106 Abcam, Cambridge, MA, United States), GAPDH 1:50000 (cat #8795 Sigma-Aldrich, Corp., St. Louis, MO, United States), β-tubulin 1:5000 (cat # T0198, Sigma-Aldrich, Corp., St. Louis, MO, United States) and Horseradish peroxidase-linked secondary antibody 1:5000 anti-mouse and anti-rabbit from Calbiochem (Calbiochem, La Jolla, CA, United States).

### Cell Culture

Primary rat aortic vascular smooth muscle cells (RASMCs) were prepared from Sprague-Dawley rats (200–250 g). All animal experiments were performed in accordance with the Guide for the Care and Use of Laboratory Animals (8th Edn, 2011) and were approved by the institutional bioethics Committee from the School of Medicine of Emory University. RASMCs were prepared by removing the thoracic aorta and cleaned it in Hanks’ balanced salt solution. Then vessels were incubated in Hanks’ solution with 175 units/mL of collagenase at 37°C for 30 min. Then adventitia and endothelium were removed from the tissue and incubated overnight in Dulbecco’s modified Eagle’s medium (DMEM) with 10% fetal bovine serum (FBS) at 37°C with 95% O_2_ and 5% CO_2_. To complete the enzymatic digestion, aortas were incubated in Hanks’ solution with collagenase 175 units/mL and elastase 0.5 mg/mL for 2 h. The digestion was terminated by addition of 10 mL of DMEM with 20% of calf serum. VSMCs were centrifuged and plated at 1 × 10^4^ cells/cm^2^ in DMEM with 10% fetal calf serum, 2 mM glutamine and antibiotics (penicillin and streptomycin). Cells were gradually weaned to 10% calf serum after three passages. Passages from 5 to 10 were used for experiments.

The A7r5 cell line, derived from embryonic rat aorta, was purchased from the American Type Culture Collection (ATCC, CRL-1444). A7r5 cells were cultured in DMEM supplemented with 10% FBS and 2 mM pyruvate and incubated at 37°C with 95% O_2_ and 5% CO_2_. Prior to stimulation, 80–90% confluent RASMCs and A7r5 were partially serum-starved overnight by incubating them in DMEM 2% FBS. Experiments were performed between passages 6 to 8.

### Western Blot

Vascular smooth muscle cells were lysed using the RIPA lysis buffer (Tris-HCl 10 mmol/L, EDTA 5 mmol/L, NaCl 50 mmol/L, 1% deoxycholic acid and 1% Triton X-100, pH 7.4). Protein concentration was determined in A7r5 line by Bradford method (BioRad protein assay) while in RASMCs, protein concentration was determined by the bicinchoninic acid assay (Pierce BCA protein assay, Thermo Scientific). Equal amounts of protein from cells were separated by 7–15% SDS polyacrylamide gel electrophoresis and electrotransferred to PVDF membranes and blocked with 5% defatted milk in Tris-buffered saline pH 7.6, containing 0.1% (v/v) Tween 20 (TBS-T). Membranes were incubated with the primary antibodies at 4°C overnight. Membranes were then incubated with horseradish peroxidase-linked secondary antibody in 1% (w/v) defatted milk in TBST. The bands were detected using ECL (cat # NEL103001EA, Perkin Elmer, Waltham, MA, United States) luminescence was assessed using a digital imaging system (Syngene). Quantification of the bands by densitometry was performed using UN-SCAN-IT gel software. Protein content was normalized by β-tubulin or GAPDH.

### Autophagy

Autophagy was evaluated by measuring LC3 II content by Western blotting and by the quantitation of autophagosome vesicles. Autophagic flux were evaluated in A7r5 and RASMCs using chloroquine (CQ) 30 μM during the last 4 h of the stimulation with Ang II (1–100 nM for dose response and 100 nM for rest of the experiments). Autophagosome vesicles were visualized in the A7r5 cell line seeded in 12 well plates with glass coverslips (18 mm) containing 3 × 10^5^ cells per well. Cells were incubated in DMEM, 2% FBS for 24 h and transduced with the adenovirus LC3-GFP (Ad LC3-GFP) for 24 h using a multiplicity of infection (MOI) of 180. After transduction, cells were stimulated with Ang II 100 nM for 24 h in the presence or absence of CQ and losartan. Cells were washed with cold PBS and fixed with 4% paraformaldehyde and the nuclei was stained with Hoechst (1:1000). The images were analyzed using a Carl Zeiss Pascal 5 confocal microscopy. Inhibition of autophagy were performed using autophagy inhibitor spautin-1 (Liu et al., 2011) and siRNA against Beclin-1. Cells were pretreated with spautin 1, 10 μM, 1 h prior to stimulation with Ang II. For genetic inhibition of autophagy, cells were transfected with 100 nmol/L of siRNA scrambled or siRNA against Beclin-1 using oligofectamine (Life Technologies) in Optimem medium (Life Technologies) for 6 h, following the manufacturer’s instructions and then stimulated with Ang II.

### VSMCs Hypertrophy

A7r5 cells were seeded in 12 well plates with glass coverslips (18 mm) containing 3 × 10^5^ cells per well. Cells were incubated in DMEM, 2% FBS for 24 h and then stimulated with Ang II 100 nM for 24 h in the presence or absence of CQ. Cells were washed with cold PBS and permeabilized with Triton X-100. Cells then were stained with phalloidin–Rhodamine (1:500; F-actin staining) and fixed with 4% paraformaldehyde, the nuclei was stained with Hoechst (1:1000). The images were analyzed using a Carl Zeiss Pascal 5 confocal microscopy. At least 30 cells from randomly fields were selected and the area was measured using the ImageJ software (NIH).

### Statistical Analysis

All data are shown as mean ± standard error (SEM) of independent experiments. Data were analyzed by one-way ANOVA or Student’s *t*-test, according to the experiment. Statistical significance was defined as *p* < 0.05. Newman–Keuls was used as *post hoc* test.

## Results

### Ang II Induces Autophagy in VSMCs

**FIGURE 1:**
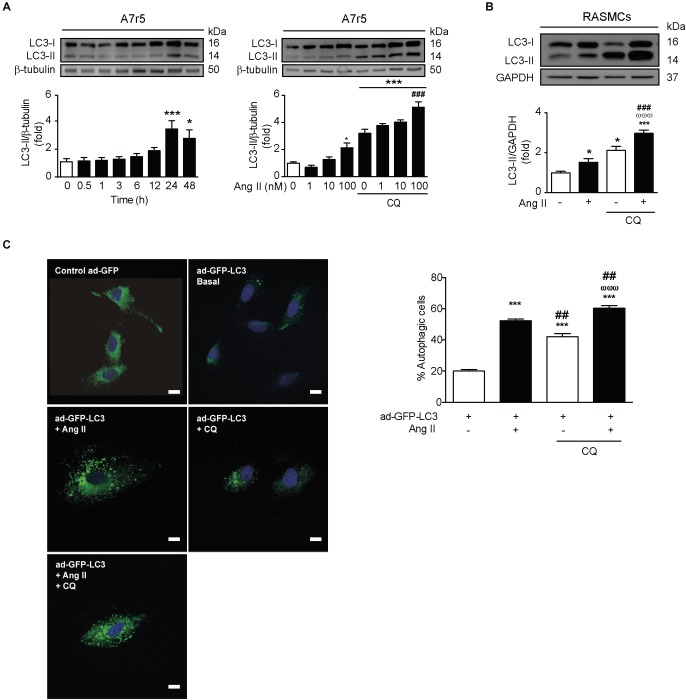
Ang II induces autophagy in A7r5 and RASMCs. **(A)** A7r5 cells were stimulated with Ang II 100 nM for 0, 0.5, 1, 3, 6, 12, 24, and 48 h (left panel) and with 1, 10, and 100 nM for 24 h, in presence and absence of CQ 30 μM, added for the last 4 h of stimulus (right panel). The LC3 II levels were determined by Western blot. The upper panels show the representative Western blots, whereas lower panels show the quantification of the LC3 II levels. β-Tubulin was used as loading control (*n* = 4–5). **(B)** Primary cultures of rat aortic VSMCs (RASMCs) were stimulated with 100 nM of Ang II for 24 h in the presence and absence of CQ 30 μM, added during the last 4 h of stimulus. LC3 II levels and autophagic flux were determined by Western blot. β-Tubulin was used as loading control (*n* = 4). **(C)** A7r5 cells were transduced with an adenovirus overexpressing LC3-GFP (ad-LC3-GFP), using a MOI of 180 and Hoechst as nuclear stain. After 24 h of incubation, cells were stimulated with 100 nM of Ang II for 24 h. During the last 4 h of stimulus, cells were then incubated in the presence or absence of 30 μM CQ. Representative images were obtained with a confocal microscope using a 40x lens and data are expressed percentage of autophagic cells (*n* = 3, 30 cells per n). Scale bar = 25 μm. The results are shown as mean ± SEM. Data were analyzed using ANOVA. Newman–Keuls was used as *post hoc* test. ^∗^*p* < 0.05, ^∗∗∗^*p* < 0.001 vs. control; ^##^*p* < 0.01, ^###^*p* < 0.001 vs. Ang II 100 nM, ^ωωω^*p* < 0.001 vs. CQ.

In order to evaluate if Ang II promotes autophagy, we stimulated A7r5 cells with Ang II 100 nM for 0, 0.5, 1, 3, 6, 12, 24, and 48 h and measured LC3 II levels by western blot. We observed that Ang II treatment gradually increased the expression of LC3 II peaking at 24 h (Figure 1A). The LC3 II increase triggered by Ang II occurs in a dose-dependent manner (Figure 1A). Then, we assess autophagic flux by concomitant administration of CQ (30 μM) during the last 4 h of a 24 h treatment with Ang II 100 nM. The further accumulation of LC3 II in the CQ-treated A7r5 and RASMCs suggest that Ang II increased the autophagic flux (Figure 1A,B). The accumulation of LC3-containing autophagic vesicles (punctuated pattern, Figure 1C) induced by Ang II in the presence of CQ (Figure 1C) further confirms that Ang II induces autophagic flux.

### Ang II Induces the Initiation and Elongation of Phagophore in VSMCs

**FIGURE 2:**
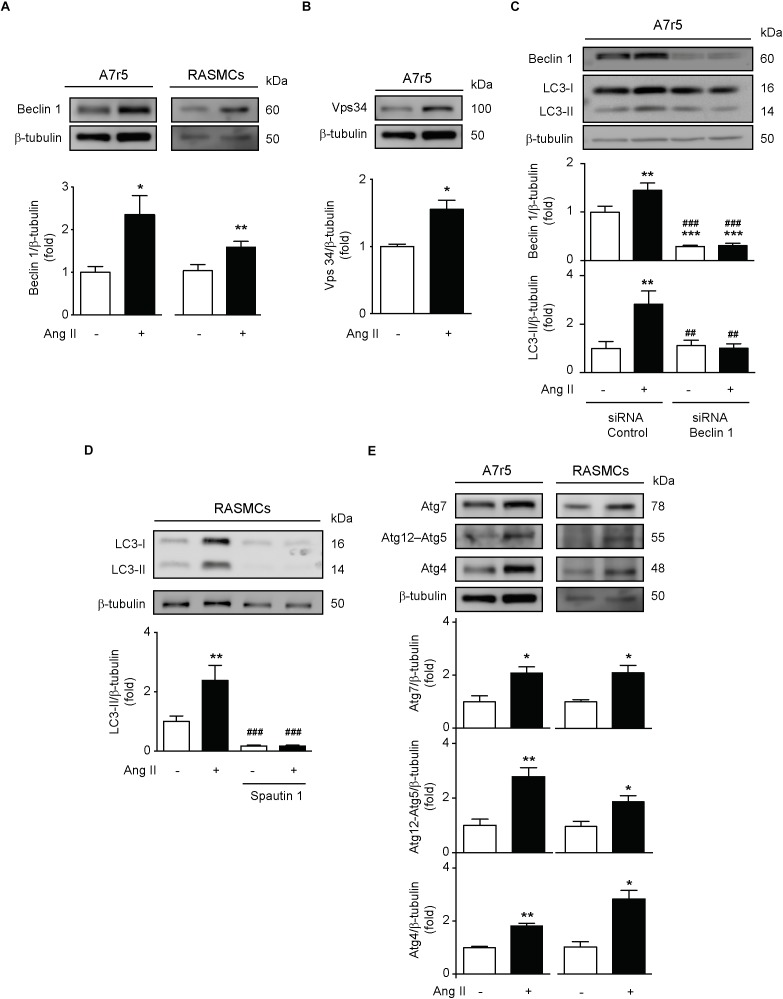
Ang induces phagophore initiation and elongation in A7r5 and RASMCs. **(A)** A7r5 and RASMCs cells were stimulated with Ang II 100 nM for 24 h. Protein levels of Beclin 1 were determined by Western blot in A7r5 and RASMCs and were normalized by β-tubulin (*n* = 4–5). **(B)** A7r5 cells were stimulated with Ang II 100 nM for 24 h and Vps34 levels were measured by Western blot and normalized to β-tubulin (*n* = 3). **(C)** A7r5 cells were transfected with 100 nM of siRNA against Beclin 1 for 6 h and incubated in DMEM with 2% of FBS for 16 h. Then, cells were stimulated with Ang II 100 nM for 24 h and protein levels of Beclin 1 and LC3 II were evaluated by Western blot and normalized by β-tubulin (*n* = 5). **(D)** Primary culture of RASMCs were pre-treated with Spautin 1, 10 μM, 1 h before the stimulation with Ang II 100 nM for 24 h. LC3 II levels were determined by Western blot and normalized by β-tubulin (*n* = 4). **(E)** A7r5 and RASMCs cells were stimulated with Ang II 100 nM for 24 h and protein levels of Atg7, Atg12–Atg5, and Atg4 were determined by Western blot and normalized by β-tubulin. Data were analyzed using Student’s *t*-test or one way ANOVA followed by Newman–Keuls *post hoc* test. Results are shown as mean ± SEM, *n* = 3–5. ^∗^*p* < 0.05, ^∗∗^*p* < 0.01, ^∗∗∗^*p* < 0.001 vs. control; ^##^*p* < 0.01, ^###^*p* < 0.001 vs. Ang II.

Considering that autophagy is a multi-step process, we evaluated if Ang II promotes the initiation of this process in VSMCs. To assess this, we quantified the expression of the initiation protein Beclin-1 in A7r5 and RASMCs stimulated with Ang II. We observed that Ang II significantly increased the expression of this protein in both cell types (Figure 2A). Moreover, Vps34 protein expression, a class III phosphatidylinositol 3-kinase type involved in phagophore initiation (Gatica et al., 2015; Lavandero et al., 2015), was also increased in response to Ang II in A7r5 (Figure 2B). These findings were further confirmed by showing that Ang II was unable to increase LC3 II levels when Beclin-1 was knocked down using a siRNA in A7r5 cells (Figure 2C). Additionally, when Ang II was co-administered with spautin-1, which promotes Vps34 degradation (Liu et al., 2011), LC3 II protein content was not augmented (Figure 2D). Furthermore, to evaluate the effects of this peptide in the autophagosome elongation, we treated both A7r5 and primary RASMCs with Ang II and we observed an increment in Atg7, Atg12–5, and Atg4 protein levels (Figure 2E). These results suggests that Ang II increases autophagy initiation and elongation in A7r5 and RASMCs.

### AT1R Activation Mediates Ang II-Induced Autophagy

**FIGURE 3:**
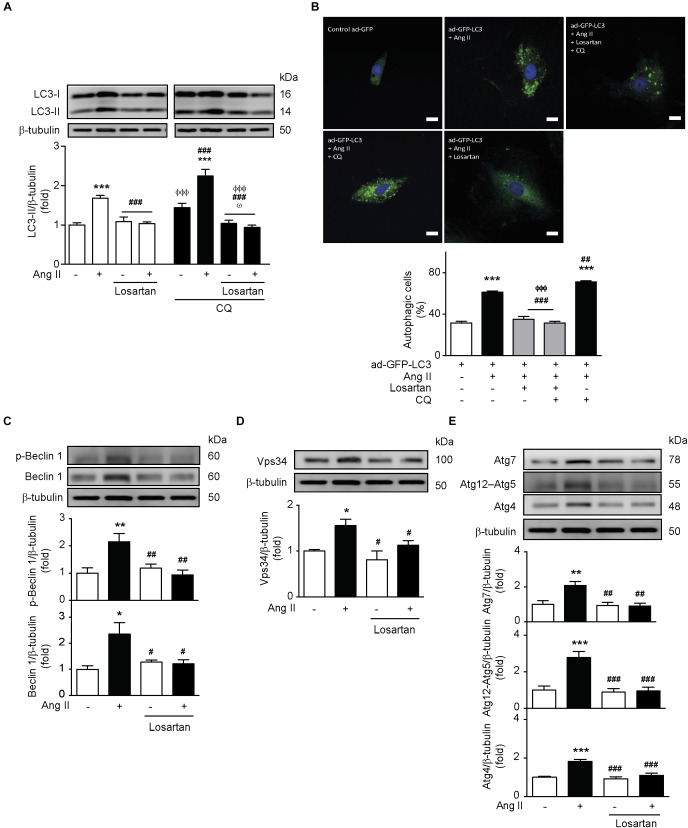
Ang II induces autophagy through an AT1R-dependent mechanism. **(A)** A7r5 cells were pre-treated with Losartan 1 μM 1 h before stimulation with Ang II 100 nM for 24 h. During the last 4 h of stimulus, cells were also treated in the presence and absence of CQ 30 μM. LC3-II levels were determined by Western blot and normalized by β-tubulin (*n* = 5). **(B)** A7r5 cells were transduced with an adenovirus overexpressing LC3-GFP (ad-LC3-GFP), using an MOI of 180. After 24 h of incubation, cells were pretreated with losartan 1 μM, 1 h before stimulation with Ang II 100 nM for 24 h. During the last 4 h of stimulus with Ang II, cells were incubated in the presence or absence of CQ 30 μM. The nucleus were stained with Hoechst. Cells were visualized by confocal microscopy. The images are representative of *n* = 3, 30 cells per n. Scale bar = 25 μm. **(C)** A7r5 cells were pre-treated with losartan 1 μM, 1 h before stimulation with Ang II 100 nM for 24 h. Levels of Beclin 1 and its phosphorylation in Thr^119^ (p-Beclin 1) were determined by Western blot and normalized by β-tubulin (*n* = 4). **(D)** A7r5 cells were pretreated with losartan 1 μM, 1 h before stimulation with Ang II 100 nM for 24 h. Vps34 levels were determined by Western blot and normalized by β-tubulin (*n* = 3). **(E)** A7r5 cells were pretreated with losartan 1 μM, 1 h before stimulation with Ang II 100 nM for 24 h. Levels of Atg7, Atg12–Atg5, and Atg4 were determined by Western blot and normalized by β-tubulin (*n* = 4–5). The results are shown as mean ± SEM. Data were analyzed using one way ANOVA followed by Newman–Keuls *post hoc* test. ^∗^*p* < 0.05, ^∗∗^*p* < 0.01, ^∗∗∗^*p* < 0.001 vs. control; ^#^*p* < 0.05, ^##^*p* < 0.01, ^###^*p* < 0.001 vs. Ang II; ^ΦΦΦ^*p* < 0.001 vs. Ang II + CQ; ^ω^*p* < 0.05 vs. CQ.

In order to investigate the participation of the AT1R in Ang II-mediated autophagy, we pre-treated A7r5 cells with losartan (1 μM) for 1 h before Ang II treatment. We observed that the increase in LC3 II levels triggered by Ang II was prevented upon pre-treatment with the AT1R antagonist (Figure 3A). This result was confirmed by quantification of the autophagic flux in the presence of CQ, followed by the assessment of LC3 II protein levels (Figure 3A) and the formation of autophagic vesicles (Figure 3B). In addition, losartan also prevented Ang II-induced initiation and elongation of the phagophore by abolishing Ang II-induced Beclin-1 phosphorylation in Thr^119^, as well as the expression of Beclin-1 (Figure 3C), Vps34 (Figure 3D), Atg7, Atg12–5, and Atg4 (Figure 3E).

### Ang II Induces Autophagy Through a ROCK-Dependent Mechanism

**FIGURE 4:**
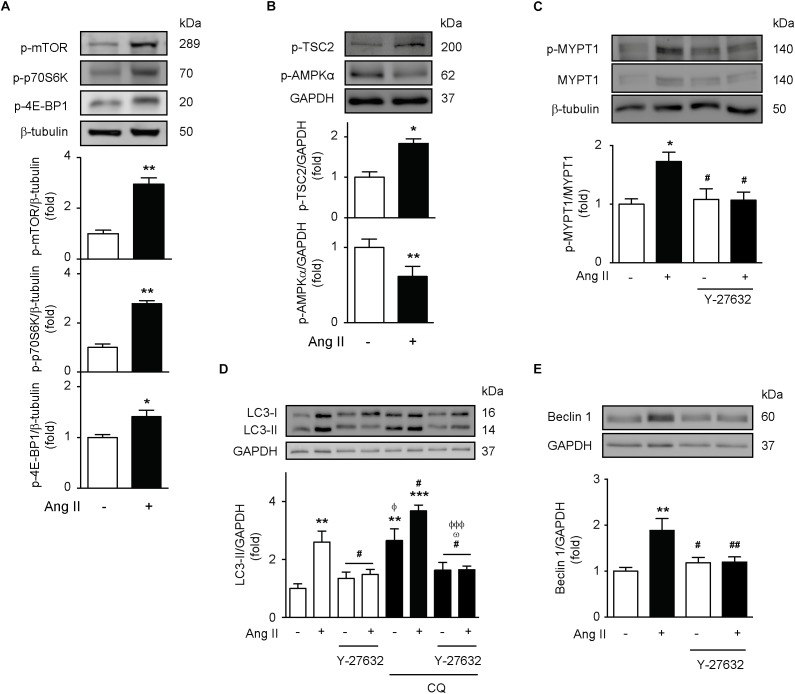
Ang II induces autophagy through a ROCK-dependent mechanism. **(A,B)** A7r5 cells were stimulated with 100 nM of Ang II for 24 h. Levels of p-mTOR Ser^2448^, p-p70S6K Thr^389^, p-4E-BP1 Thr^37/46^, p-TSC2 Thr^1462^, and p-AMPKα Thr^172^ were determined by Western blot and normalized by β-tubulin or GAPDH (*n* = 4–5). **(C)** A7r5 cells were pre-treated with Y-27632 (ROCK inhibitor, 10 μM), 1 h before stimulation with Ang II 100 nM for 24 h. ROCK activation levels were evaluated by MYPT1 phosphorylation in Thr^853^ assessed by Western blot. Protein levels were normalized by β-tubulin (*n* = 3). **(D)** A7r5 cells were pre-treated with 10 μM of Y-27632, 1 h before stimulation with Ang II 100 nM for 24 h. During the last 4 h of stimulus, cells were treated in the presence and absence of CQ 30 μM. LC3 II levels were determined by Western blot and normalized by GAPDH (*n* = 5). **(E)** A7r5 cells were pretreated with 10 μM of Y-27632, 1 h before stimulation with Ang II 100 nM for 24 h. Beclin 1 levels were determined by Western blot and normalized by GAPDH (*n* = 5). Results are shown as mean ± SEM. Data were analyzed using Student’s *t*-test or one way ANOVA followed by Newman–Keuls *post hoc* test. ^∗^*p* < 0.05, ^∗∗^*p* < 0.01, ^∗∗∗^*p* < 0.001 vs. control; ^#^*p* < 0.05, ^##^*p* < 0.01 vs. Ang II; ^Φ^*p* < 0.05, ^ΦΦΦ^*p* < 0.001 vs. Ang II + CQ; ^ω^*p* < 0.05 vs. CQ.

Since mTOR inhibition is the canonical autophagy activation mechanism (Gatica et al., 2015; Lavandero et al., 2015), we first evaluated the activity of this signaling pathway. The results show that Ang II triggers the phosphorylation of the mTOR Ser^2448^ and the mTOR downstream targets p70s6k and 4E-BP1 (Figure 4A). Moreover, Ang II also promotes TSC2 Thr^1462^ phosphorylation, as well as the inhibition of AMPK-α Thr^172^ phosphorylation (Figure 4B). Phosphorylation of Thr^1462^ inhibits TSC2 activity and induces mTOR activation (Huang and Manning, 2008). On the other hand, reduction of AMPK activity facilitates the activation of mTOR (Jeon, 2016). Thereby, our results suggest that mTOR is active after stimulation with Ang II.

Then, we investigated whether ROCK, a downstream component of the AT1R pathway, is required for the induction of autophagy triggered by Ang II. To test this, we stimulated A7r5 cells with Ang II and we determined the MYPT1 phosphorylation (a downstream target of ROCK). Ang II increased phospho-MYPT1 as compared to untreated cells, but this effect was lost upon co-administration of this peptide with the ROCK inhibitor, Y-27632 10 μM (Figure 4C). Moreover, to evaluate the role of the ROCK signaling pathway in autophagy, we measured LC3 II levels in response to Ang II. As expected, pre-treatment with Y-27632 completely abolished Ang II-dependent increase in LC3 II protein levels (Figure 4D). Additionally, ROCK inhibition also prevented Ang II-mediated increase in Beclin-1 protein content (Figure 4E).

### Ang II-Induced VSMC Hypertrophy Requires ROCK-Dependent Induction of Autophagy

**FIGURE 5:**
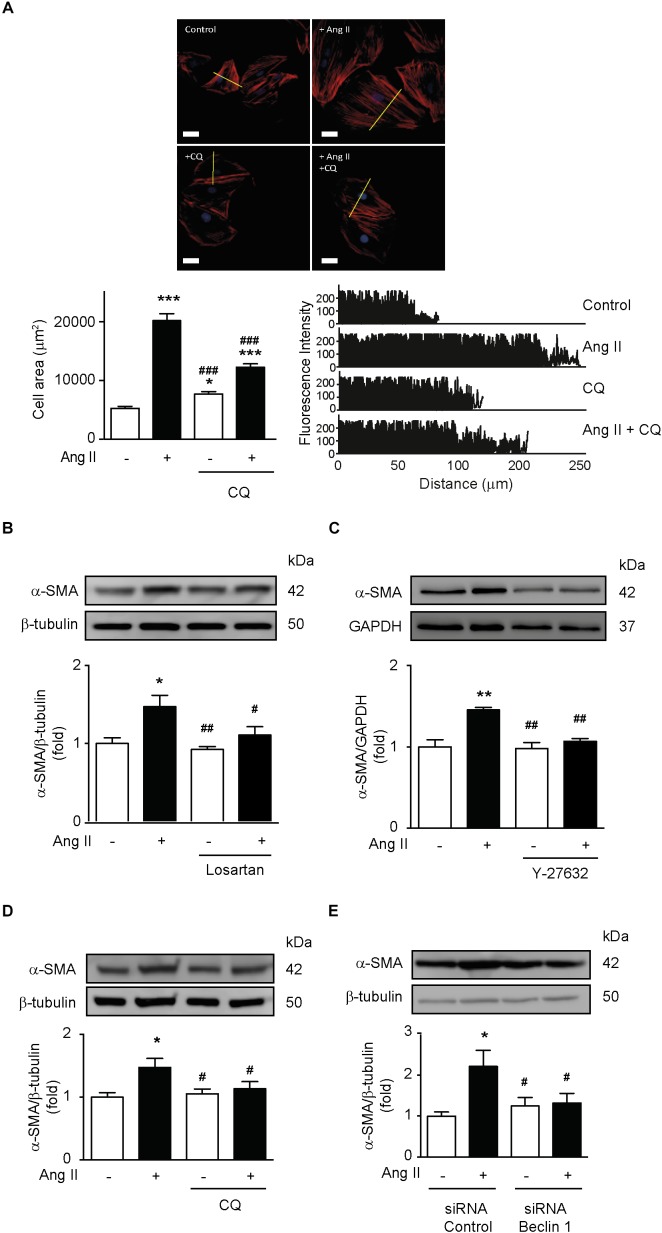
Ang II-dependent autophagy elicits hypertrophy in VSMCs. **(A)** A7r5 cells were treated with or without Ang II 100 nM. During the last 4 h of stimulus, cells were treated in the presence and absence of CQ 30 μM. Then cells were stained with phalloidin–rhodamine and nuclei were stained with Hoechst. Images were captured using a confocal microscope using 40x lens. Cell area and fluorescence intensity were measured using the ImageJ software. *n* = 3,30 cells per condition. Scale bar = 25 μm. A7r5 cells were pre-treated with **(B)** Losartan 1 μM or **(C)** Y-27632 10 μM, 1 h before stimulation with Ang II 100 nM for 24 h. During the last 4 h of stimulus, cells were treated in the presence and absence of CQ 30 μM **(D)**. **(E)** A7r5 cells were transfected with a control siRNA or siRNA against Beclin 1 100 nM for 6 h and incubated in DMEM with 2% of FBS for 16 h. Cells were then stimulated with Ang II 100 nM for 24 h. α-SMA levels were evaluated by Western blot and normalized by β-tubulin (*n* = 5). α-SMA levels were determined by Western blot and normalized by GAPDH (*n* = 5). Data were analyzed using Student’s *t*-test or one way ANOVA followed by Newman–Keuls *post hoc* test. The results are shown as mean ± SEM. ^∗^*p* < 0.05, ^∗∗^*p* < 0.01, ^∗∗∗^*p* < 0.001 vs. control; ^#^*p* < 0.05, ^##^*p* < 0.01, ^###^*p* < 0.001 vs. Ang II.

It is well-stablished that Ang II induces hypertrophy in VSMC and that α-SMA expression is a major component of this hypertrophic response (Geisterfer et al., 1988; Andrawis et al., 1993; Yoshida et al., 2004). To evaluate this, we stimulated A7r5 cells with Ang II and measured cell area and α-SMA protein levels. Ang II increased VSMCs area and fluorescence intensity, effect that was prevented by autophagy inhibition with CQ (Figure 5A). Ang II also increased α-SMA protein levels (Figure 5B). Moreover, AT1R antagonism (Figure 5B), ROCK inhibition (Figure 5C), as well as blockage of autophagy with CQ (Figure 5D) and knock down of Beclin 1 with siRNA (Figure 5E) prevented the α-SMA increase after Ang II stimulation.

## Discussion

The main findings of this study were: (i) Ang II induced autophagy in VSMCs, (ii) this peptide participated in the initiation and elongation of the phagophore, (iii) Ang II-induced autophagy is mediated by AT1R/ROCK signaling pathway and not by mTOR, and (iv) Ang II induced VSMC hypertrophy, as determined by an increase in cell area and α-SMA protein levels, through an autophagy-dependent mechanism.

Our findings show that 100 nM of Ang II activated autophagy with a maximum at 24 h of stimulus. In this context, Zhang et al. (2016), reported that this concentration of Ang II increased proliferation, migration and protein content of pro-inflammatory factors, such as MCP-1, VCAM-1, and IL-β1 in VSMCs. Moreover, cells treated with Ang II showed increased NAPDH oxidase activity and ROS production via a mechanisms involving MAPKs and PI3K/Akt signaling pathways, but these effects were lost in the presence of losartan and Ang-(1–7) (Zhang et al., 2016). In line with this, Hu et al. (2002) showed that Ang II 100 nM increased COX-2 mRNA and protein levels in VSMCs. PPAR α and γ activators abolish Ang II-dependent increase in migration and COX-2 levels (Hu et al., 2002). These alterations play an important role in the development and progression of hypertension (Oparil et al., 2018). Our results supports that autophagy may be a potential mechanism mediating the deleterious effects of Ang II in VSMCs in the context of vascular diseases. However, cause-effect experiments are required to further explore this possibility.

The link between Ang II and autophagy has been previously explored by Yu et al. (2014). The authors of this study evaluated the role of mitochondrial K_ATP_ channels in autophagy induced by Ang II in rat aortic smooth muscle cells (Yu et al., 2014). While the authors showed increased LC3 II levels in response to Ang II 100 nM, their autophagic flux experiments with bafilomycin did not produce accumulation of this protein, which indicates that autophagy was not activated. Moreover, they also evaluated formation of autophagic vesicles by immunofluorescence, but measurement of autophagic flux was not performed (Yu et al., 2014).

Our experiments with CQ confirmed that Ang II indeed triggered autophagy. However, this process requires different stages to be completed (Lavandero et al., 2015). Our data suggest that Ang II is involved in the initiation of autophagy, which was evidenced by an increase in Beclin-1 protein content and its phosphorylation in Thr^119^, along with the increased Vps34 protein levels. In order to interact with Vps34 and initiate autophagy, Beclin-1 is first dissociated from its inhibitory complex with Bcl-2 (Pattingre et al., 2005). In HeLa cells that, Beclin-1 can be phosphorylated in Thr^119^ by ROCK, thereby promoting its release from the inhibitory union with Bcl-2 (Gurkar et al., 2013). The next step in autophagy is the elongation of the phagophore (Gatica et al., 2015). Yu et al. (2014) found that knocking down Atg5 significantly reduced LC3 II levels in VSMCs treated with Ang II. However, there are no studies evaluating whether Ang II can regulate Atg7 or Atg4 in VSMCs. Our findings show that Ang II promoted an increase in the levels of Atg12–Atg5, Atg7 and Atg4, suggesting that this peptide can stimulate the elongation of the phagophore, which was confirmed by the increase in the percentage of cells with punctuated pattern. Nonetheless, while it has been thoroughly described that ATGs participate in the autophagic process (Mizushima et al., 1998; Mizushima et al., 2011), the mechanisms by which they are regulated, remains to be fully understood. It has been reported that the transcriptional factor FOXO1 can induce the expression of Atg7, Atg5 and Atg12, thus eliciting the induction autophagy (Zhao et al., 2010; Xu et al., 2011). Moreover, Qi et al. (2014) showed that in liver-specific FOXO1 knockout mice, blood pressure, as well as plasma levels of angiotensinogen and Ang II were reduced. Although this study was performed in liver tissue, the link between FOXO1 and Ang II further suggest this transcriptional factor may be responsible for the modulation of ATGs in the autophagy induced by Ang II in VSMCs, but future experiments should clarify this point.

The classic pathway to induce autophagy requires mTOR inhibition (Gatica et al., 2015). Nevertheless, our results showed that Ang II activated mTOR signaling pathway. Accordingly, it has been previously described that Ang II can activate the mTOR pathway by increasing the phosphorylation of its downstream targets, p70S6K and 4E-BP1 and that may be associated with higher vascular damage and hypertrophy (Haider et al., 2002; Yamakawa et al., 2003; Hafizi et al., 2004; Li et al., 2004; Zhu et al., 2012). mTOR is a key regulator of protein synthesis (Fonseca et al., 2014). Consistent with the role of mTOR as key determinant of the rate of protein synthesis, our findings indicate that Ang-induced VSMC hypertrophy requires mTOR activation. It’s important to note that while we do not show mTOR activation elicited by Ang II in RASMCs, the activation of the mTOR signaling pathway by Ang II has been described in primary cultures of aortic and coronary smooth muscle cells of rats and humans, respectively (Haider et al., 2002; Hafizi et al., 2004).

Under nutrient deprivation conditions, mTOR is inhibited and autophagy is activated (Jung et al., 2010). Under this setting, there is a decrease in the AMP/ATP ratio, thus triggering AMPK activation, which in turn, can inhibit mTOR by phosphorylating raptor and TSC2 (Gwinn et al., 2008; Jung et al., 2010). On the other hand, in the presence of nutrients, mTOR inhibits autophagy by a mechanism that involves ULK1 and AMBRA1 phosphorylations (Ganley et al., 2009; Nazio et al., 2013). Our findings suggest that Ang II-induced autophagy does not inhibit mTOR or activates AMPK. Therefore, we sought to explore the potential mechanism by which Ang II can promote autophagy by assessing the role of the AT1R in this effect. Our results indicated that pre-treatment with losartan prevented the Ang II-mediated increase in LC3 II, Vps34 and Beclin-1 levels, as well as Beclin 1 phosphorylation in Thr^119^. In addition autophagic flux and formation of autophagic vesicles were also decreased. Accordingly, Yu et al. (2014) found that pre-treatment with olmesartan and candesartan abolished the increase in LC3 II levels, although this study does not show autophagic flux experiments in the presence of AT1R antagonists.

Interestingly, Porrello et al. (2009) reported that Ang II administration in neonatal rat ventricular myocytes overexpressing AT1R, showed increased hypertrophy and higher autophagic activity, thereby increasing the number of autophagosomes. Moreover, autophagy was diminished in the presence of candesartan. In addition, they observed that AT2R overexpression reduced Ang II-induced autophagy even in presence of AT1R overexpression. Therefore, their results suggest that Ang II induces autophagy in cardiomyocytes by an AT1R dependent mechanism and that AT2R may antagonize this effect (Porrello et al., 2009). Moreover, Lin et al. (2014) showed that mechanical stress induced an increase in hypertrophy and autophagy in cardiomyocytes. However, these effects were reduced by losartan, but not with PD123319, which antagonizes AT2R, treatment (Lin et al., 2014).

The role of AT1R in the elongation of the phagophore has been only partially studied. Zhang et al. (2014) described that in a porcine model of renovascular hypertension, autophagy was activated in the myocardium, which was evidenced by an increase in LC3 II, Beclin-1 and Atg12–Atg5 (which is involved in phagophore elongation). This effect was prevented upon AT1R antagonism with valsartan. Our results further support these findings, given that losartan abolished the Ang II-induced increase in Atg7, Atg12–5 and Atg4 protein levels.

Pursuing AT1R downstream targets, we assessed the role of RhoA/ROCK in the Ang II-induced autophagy. This pathway is active in hypertension, given that, this disease is characterized by high levels of Ang II. Moreover, the RhoA/ROCK signaling pathway is a key regulator of vascular tone and remodeling induced by AT1R activation (Noma et al., 2006). ROCK has 2 isoforms; ROCK1 is expressed in lung, liver, spleen, testicles and kidney, whereas ROCK2 is mainly present in brain and heart (Wirth, 2010; Satoh et al., 2011). Thus, we used Y-27632, a ROCK inhibitor, which can inhibit both isoforms by binding to their catalytic site (Ishizaki et al., 2000). ROCK inhibition by Y-27632 was confirmed by assessing a reduction in the phosphorylation of MYPT1 in Thr^853^, which is the gold standard to evaluate the ROCK activity (Kitazawa et al., 2003; Dimopoulos et al., 2007; Khromov et al., 2009). Our data showed that increased autophagy in response to Ang II is lost upon pre-treatment with Y-27632. These findings are in agreement with the study performed by Gurkar et al. (2013) which described that ROCK phosphorylates Beclin-1 in Thr^119^ to activate autophagy.

Our data show that Ang II-dependent autophagy induced an increase in cell area and α-SMA protein levels in VSMCs. In hypertension, which involves remodeling of the vascular wall, α-SMA is increased in comparison to healthy blood vessels (Schildmeyer et al., 2000; Rzucidlo et al., 2007; Goulopoulou and Webb, 2014; Liu et al., 2015). Inhibition of ROCK-induced autophagy should be explored as a selective mechanism to reduce vascular damage during hypertension.

From a translational perspective, it’s important to highlight that *in vivo* studies linking autophagy to vascular hypertrophy are scarce. Martinet and De Meyer (2009) described that autophagy was increased in atherosclerotic plaques, suggesting its relevance in vascular diseases and therefore, this may be extrapolated to hypertension, but a potential effect in hypertrophy remains to be explored. Moreover, given that autophagy is a ubiquitous process that occurs in virtually all cells (Yin et al., 2016), it can be induced in an *in vivo* setting using starvation models (Mizushima et al., 2004). Nonetheless, there are no selective inhibitors of autophagy and therefore, there are still multiple obstacles to overcome in the road to a translational approach of the therapeutic targeting of autophagy in vascular diseases.

One of the main limitations of our study is that, although key findings were observed with RASMCs, not all experiments were performed using primary cells and future research should thoroughly address the role of autophagy in angiotensin II-induced hypertrophy using animal models in order to further confirm our results. In addition, we used CQ to block autophagy, but interestingly, it has been recently reported that its mechanism of action may involve defective fusion of the lysosome with the autophagosome, instead of modulating the acidity of lysosomes (Mauthe et al., 2018). Moreover, CQ was found to induce disorganization of the endo-lysosomal system and the Golgi apparatus via a mechanism that is independent of autophagy, which could be involved in the blockade of the fusion process (Mauthe et al., 2018). Given the off-target effects of CQ, a comparison with another compound to evaluate autophagic flux, such as Bafilomycin A1 may be needed in order to thoroughly confirm our findings.

Taken together, our results indicate that Ang II induces VSMC hypertrophy through a mechanism that involves autophagy activation via AT1R/ROCK-dependent signaling pathway. Therefore, autophagy may be a novel therapeutic target to prevent VSMC hypertrophy during Ang II-induced hypertension.

## Author Contributions

DM-R, ASM, CQ, LG, SL, and MC contributed to conception and design of the study. DM-R, IN-S, FS-O, PV-F, TH-D, and NC-A performed the experiments. DM-R performed the statistical analysis. DM-R and JR wrote the first draft of the manuscript. MC and ASM wrote the sections of the manuscript. All authors contributed to manuscript revision, read and approved the submitted version.

## Conflict of Interest Statement

The authors declare that the research was conducted in the absence of any commercial or financial relationships that could be construed as a potential conflict of interest. The reviewer MG and handling Editor declared their shared affiliation.

## Supplementary Material

FIGURES S1–S5 File containing all the original uncropped western blot images depicted in the Figures 1(A,B), 2(A–E), 3(A,C–E), 4(A–E), and 5(B–E).
