# Supplementary figures and images for: Angiotensin II-Regulated Autophagy Is Required for Vascular Smooth Muscle Cell Hypertrophy
Source: Front Pharmacol. 2019 Feb 5;9:1553. doi: 10.3389/fphar.2018.01553 (PMC6371839; doi:10.3389/fphar.2018.01553)

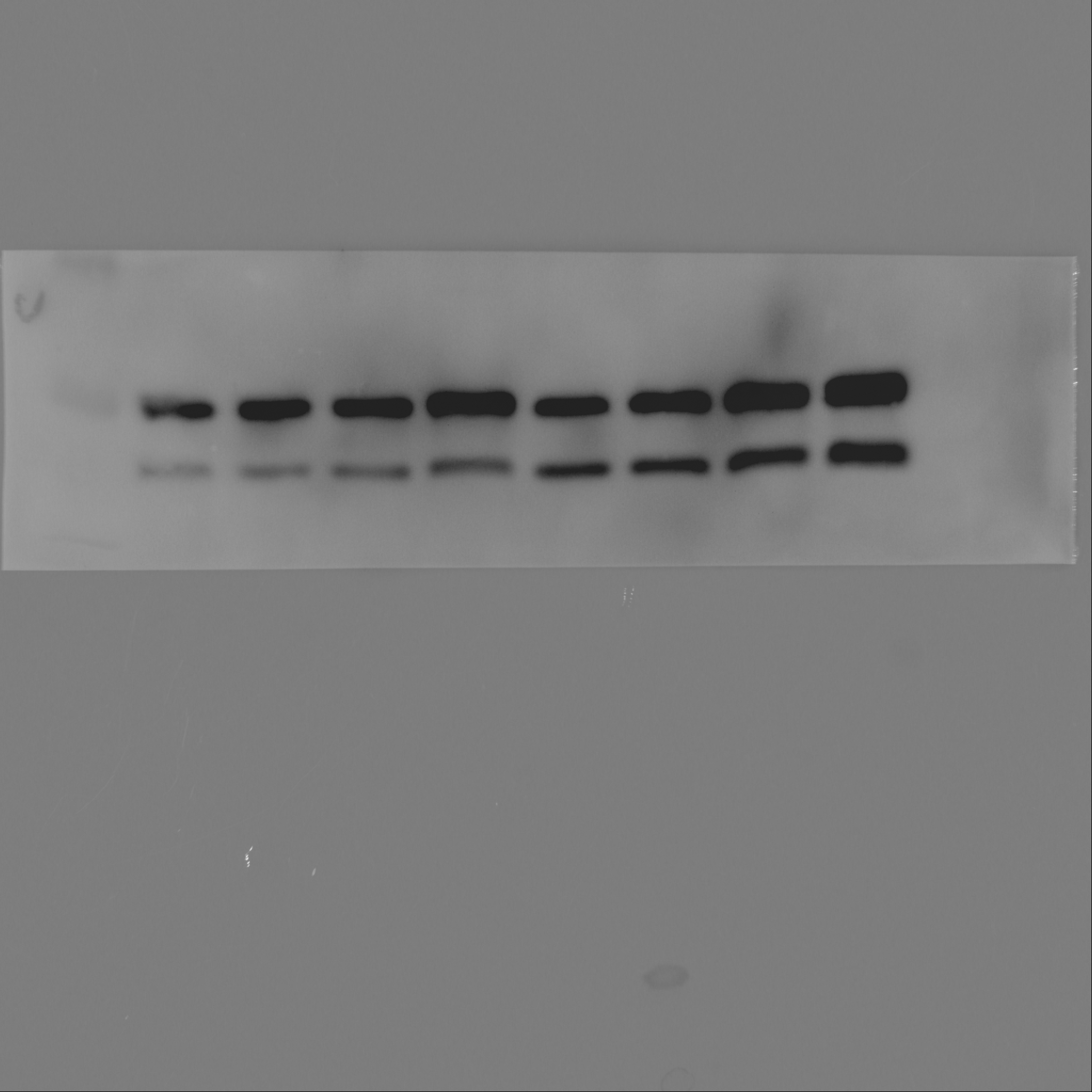

Supplement: FIGURES S1–S5 — File containing all the original uncropped western blot images depicted in the Figures 1(A,B), 2(A–E), 3(A,C–E), 4(A–E), and 5(B–E). [file Data_Sheet_1.ZIP › Figure 1 A/Dose Response/LC3/image.tif]

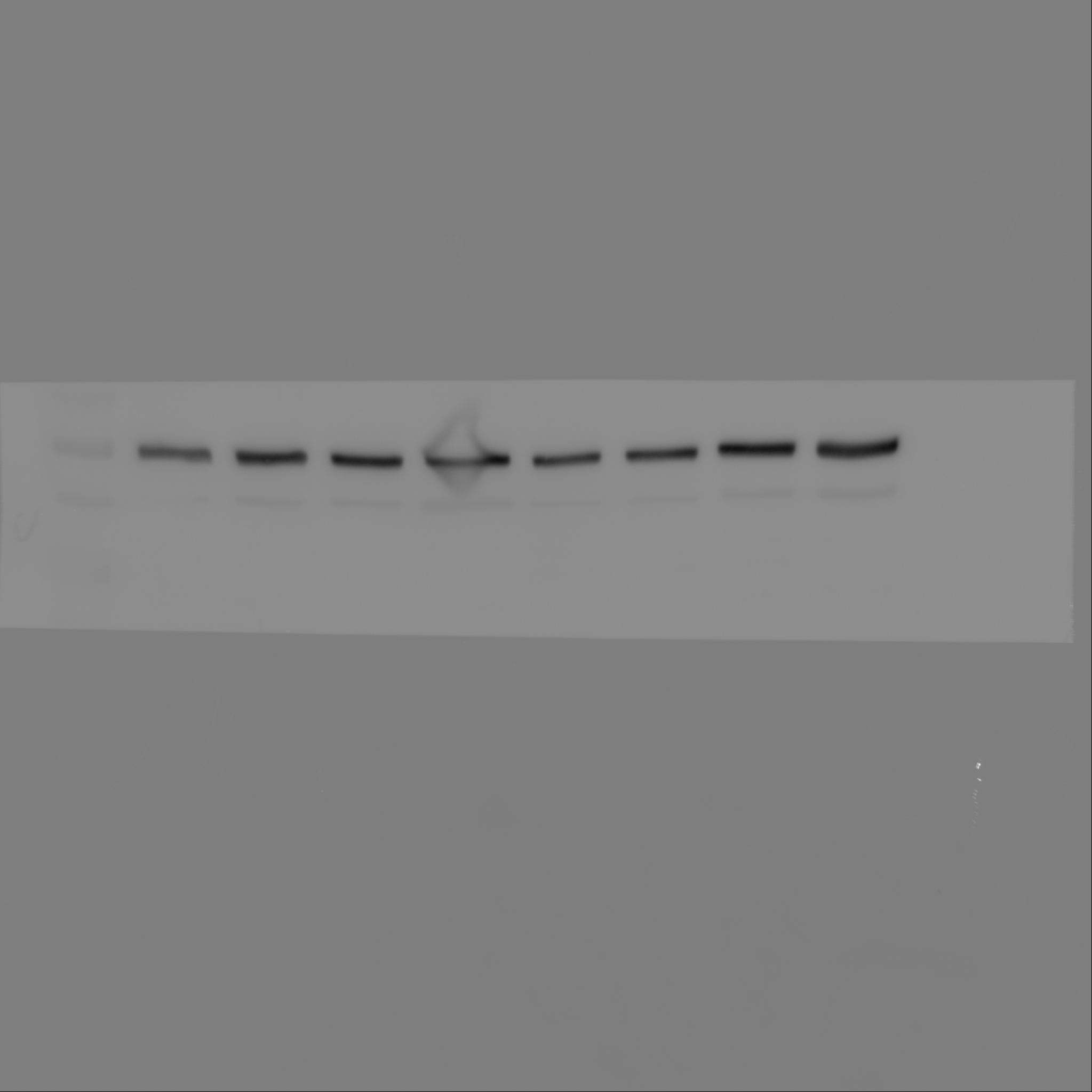

Supplement: FIGURES S1–S5 — File containing all the original uncropped western blot images depicted in the Figures 1(A,B), 2(A–E), 3(A,C–E), 4(A–E), and 5(B–E). [file Data_Sheet_1.ZIP › Figure 1 A/Dose Response/Tubulin/image.tif]

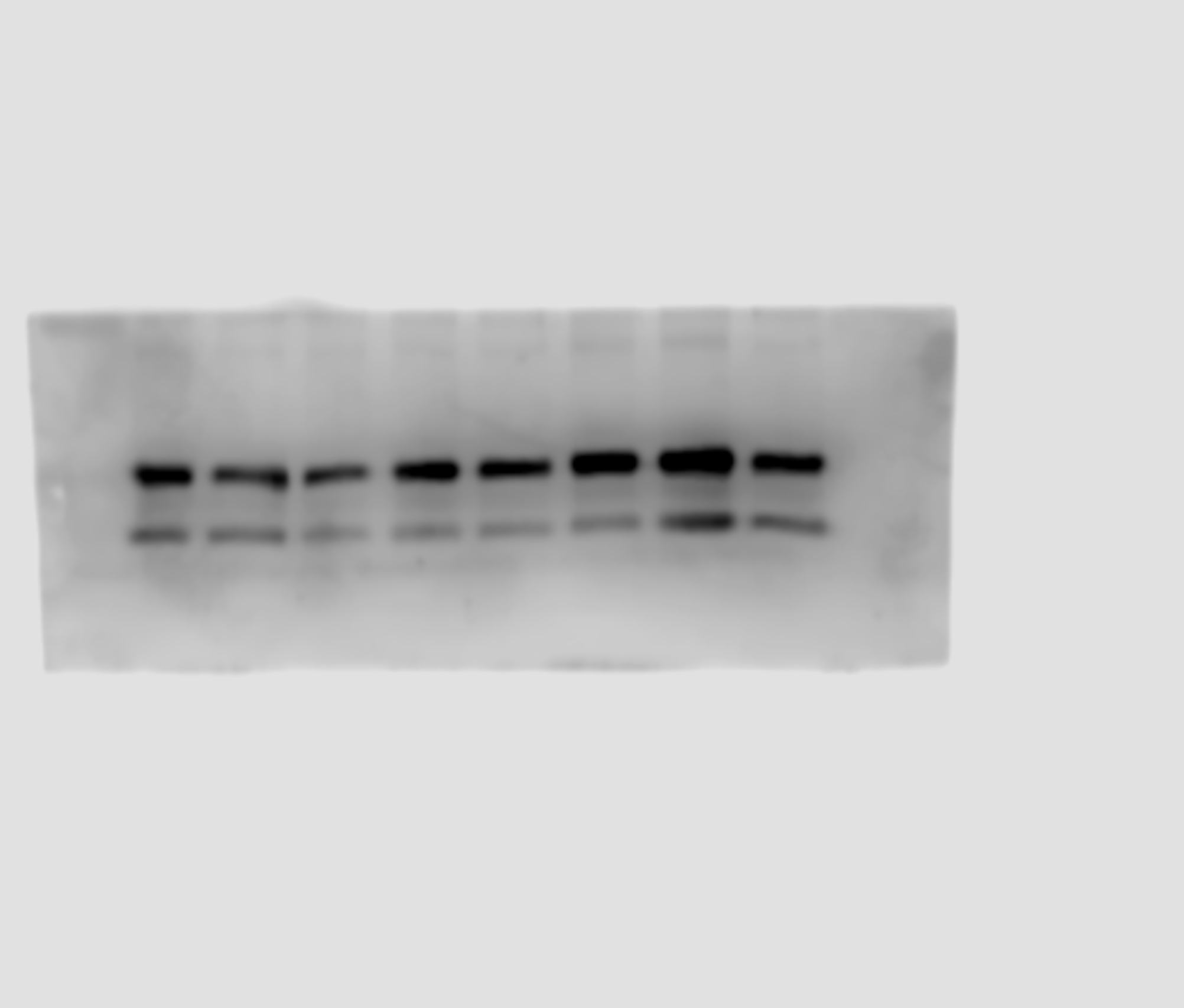

Supplement: FIGURES S1–S5 — File containing all the original uncropped western blot images depicted in the Figures 1(A,B), 2(A–E), 3(A,C–E), 4(A–E), and 5(B–E). [file Data_Sheet_1.ZIP › Figure 1 A/Kinetics/LC3/image.tif]

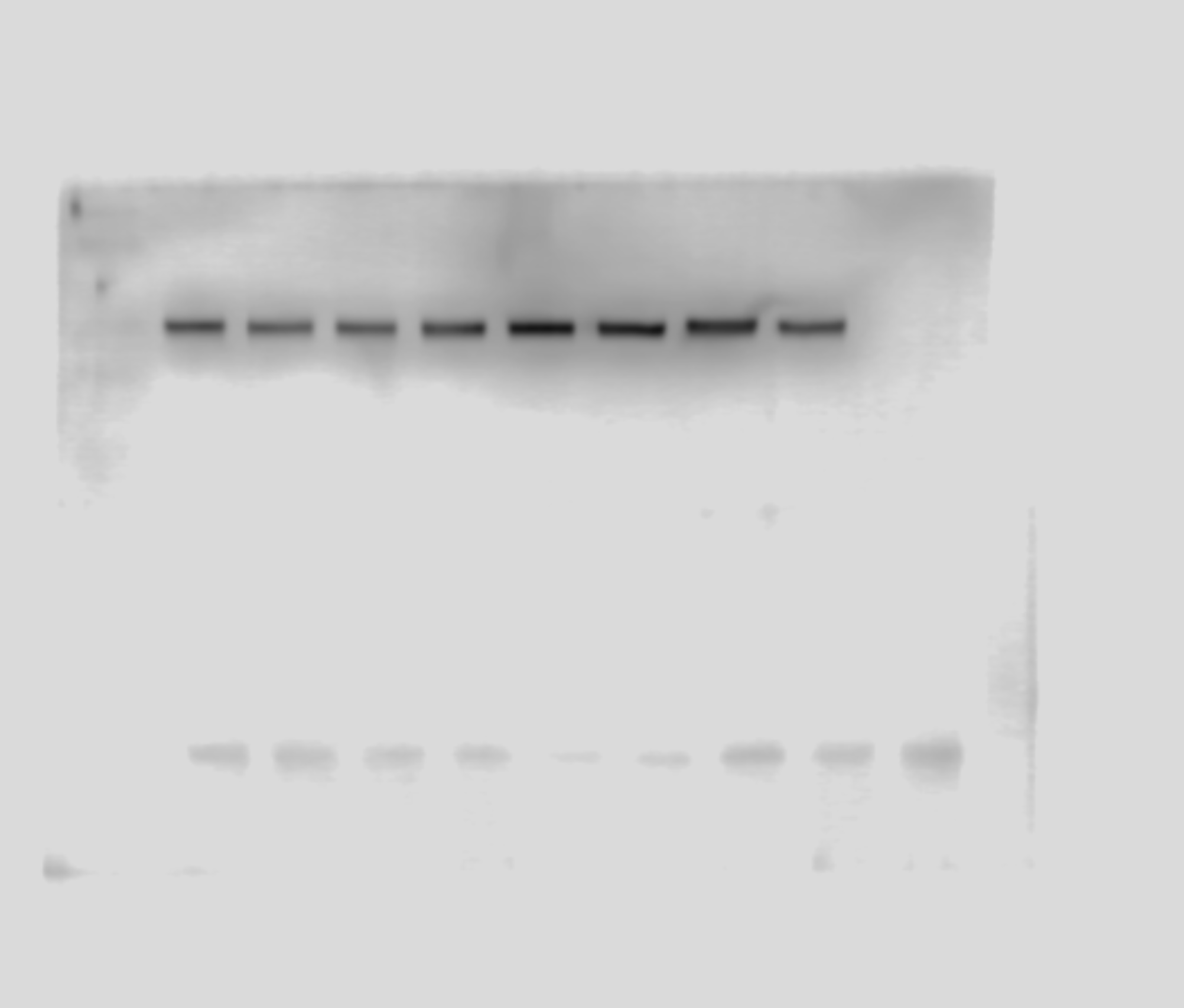

Supplement: FIGURES S1–S5 — File containing all the original uncropped western blot images depicted in the Figures 1(A,B), 2(A–E), 3(A,C–E), 4(A–E), and 5(B–E). [file Data_Sheet_1.ZIP › Figure 1 A/Kinetics/Tubulin/image.tif]

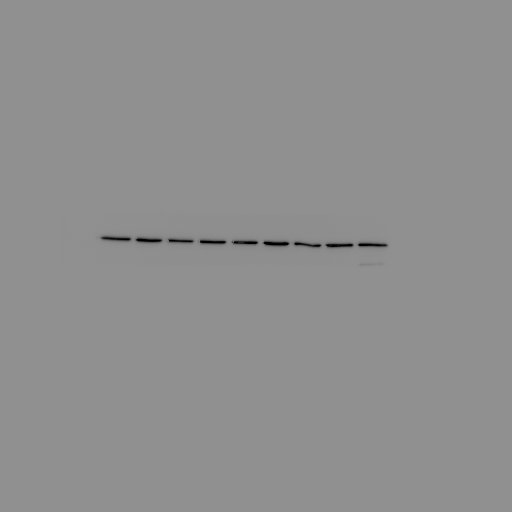

Supplement: FIGURES S1–S5 — File containing all the original uncropped western blot images depicted in the Figures 1(A,B), 2(A–E), 3(A,C–E), 4(A–E), and 5(B–E). [file Data_Sheet_1.ZIP › Figure 1 B/GAPDH/image.jpg]

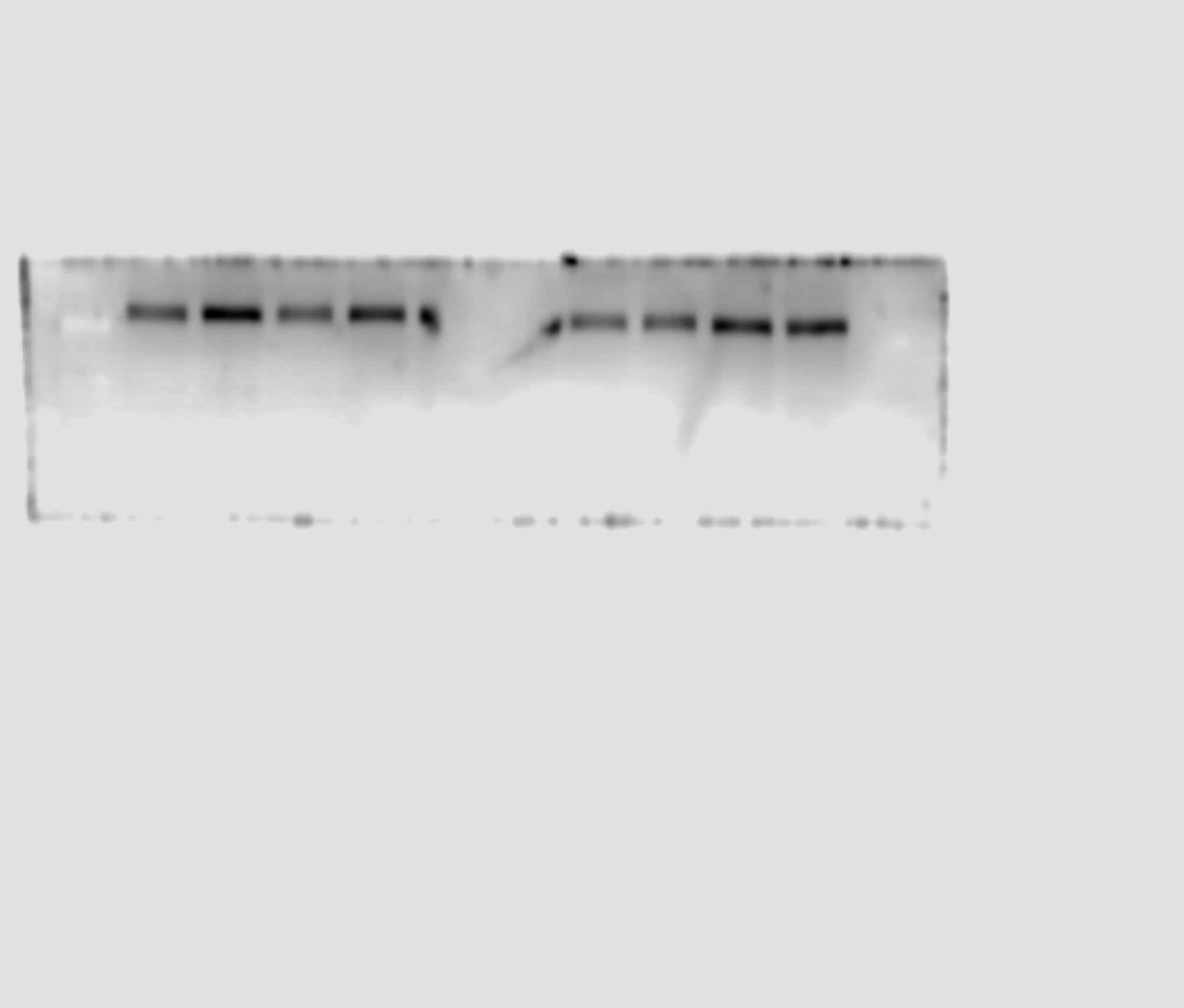

Supplement: FIGURES S1–S5 — File containing all the original uncropped western blot images depicted in the Figures 1(A,B), 2(A–E), 3(A,C–E), 4(A–E), and 5(B–E). [file Data_Sheet_1.ZIP › Figure 2 A/A7r5/Beclin1/beclin1.tif]

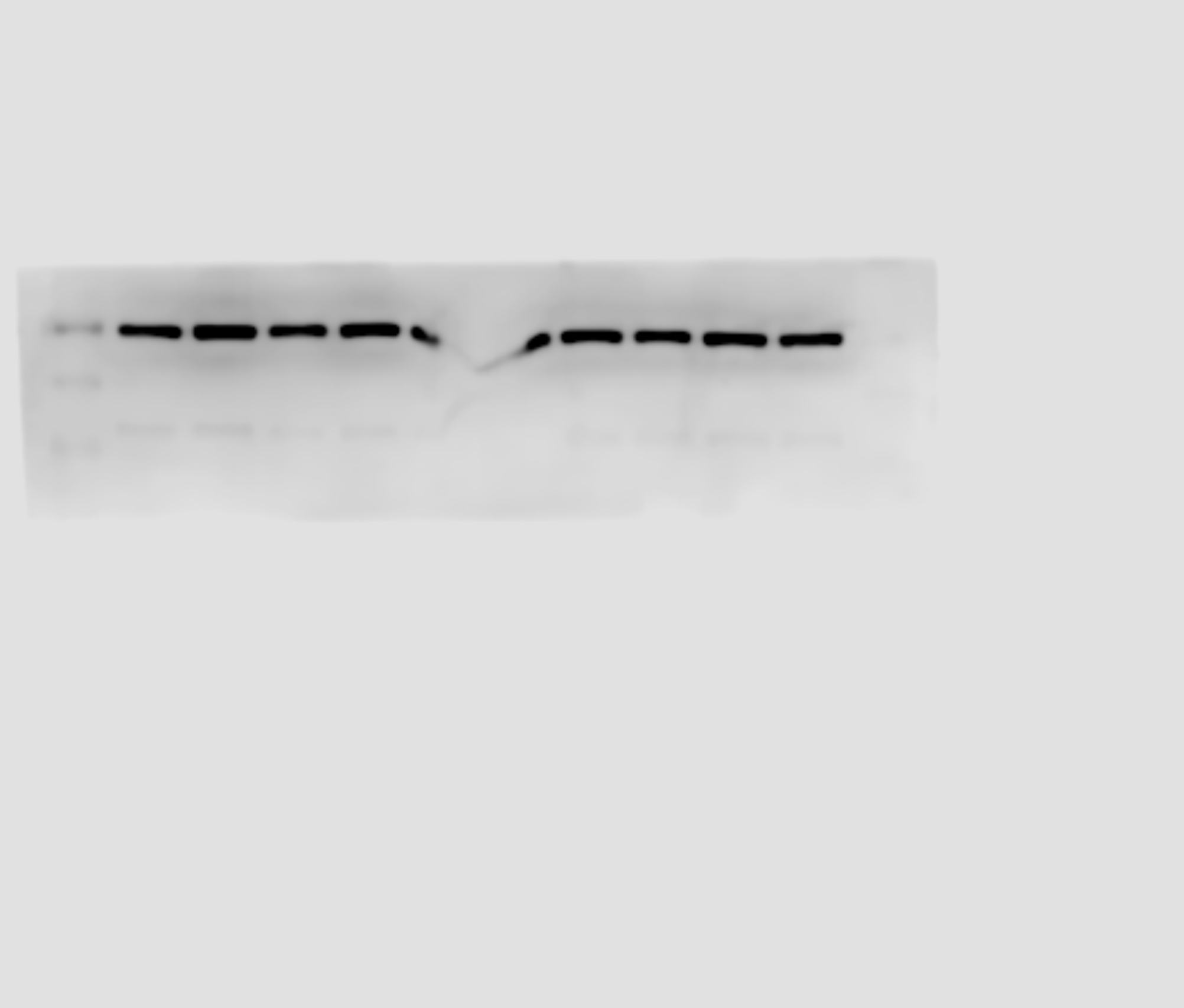

Supplement: FIGURES S1–S5 — File containing all the original uncropped western blot images depicted in the Figures 1(A,B), 2(A–E), 3(A,C–E), 4(A–E), and 5(B–E). [file Data_Sheet_1.ZIP › Figure 2 A/A7r5/Tubulin/Image_0000202_01 b.tif]

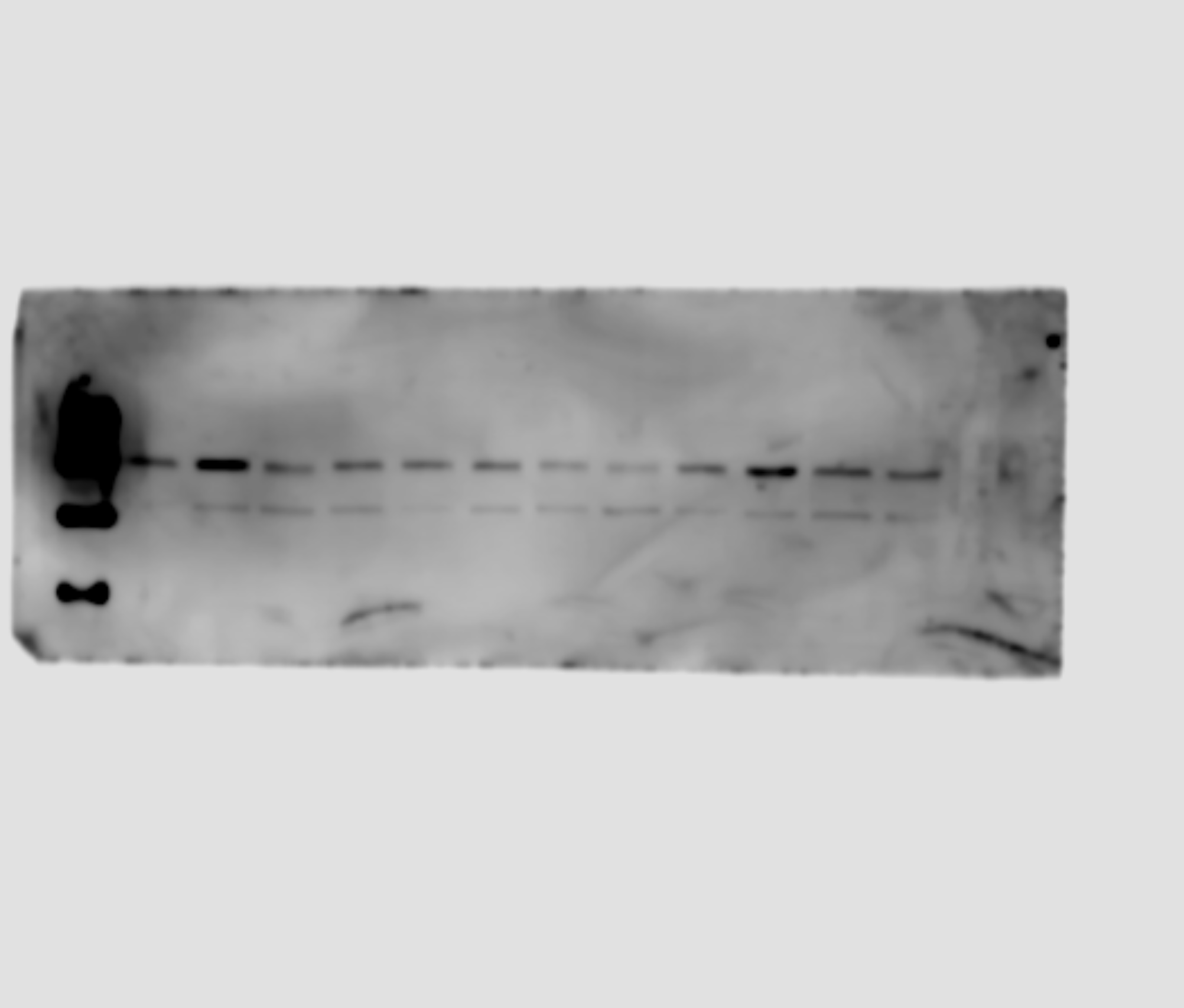

Supplement: FIGURES S1–S5 — File containing all the original uncropped western blot images depicted in the Figures 1(A,B), 2(A–E), 3(A,C–E), 4(A–E), and 5(B–E). [file Data_Sheet_1.ZIP › Figure 2 A/RASMs/Beclin1/Beclin1.tif]

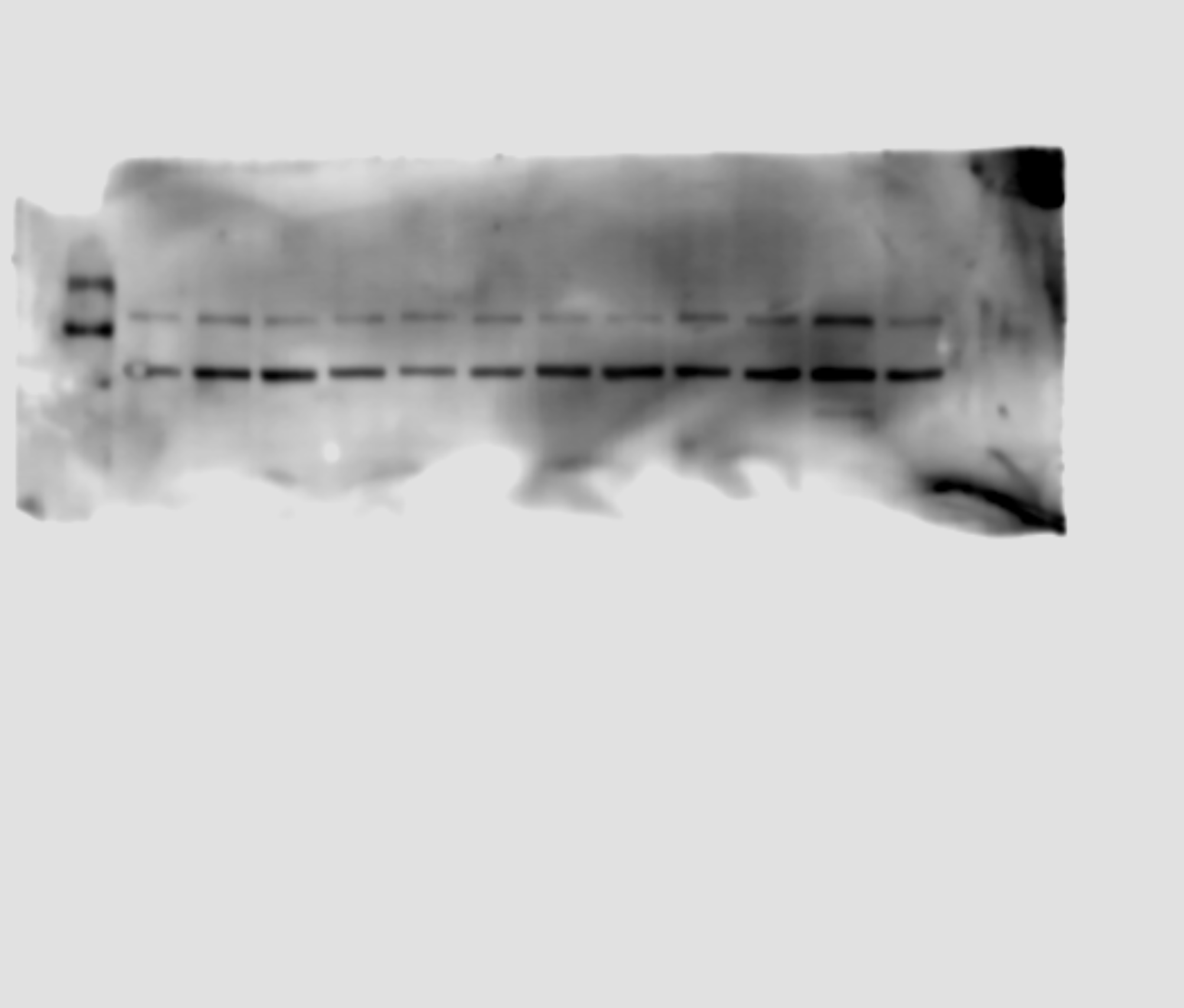

Supplement: FIGURES S1–S5 — File containing all the original uncropped western blot images depicted in the Figures 1(A,B), 2(A–E), 3(A,C–E), 4(A–E), and 5(B–E). [file Data_Sheet_1.ZIP › Figure 2 A/RASMs/Tubulin/image.tif]

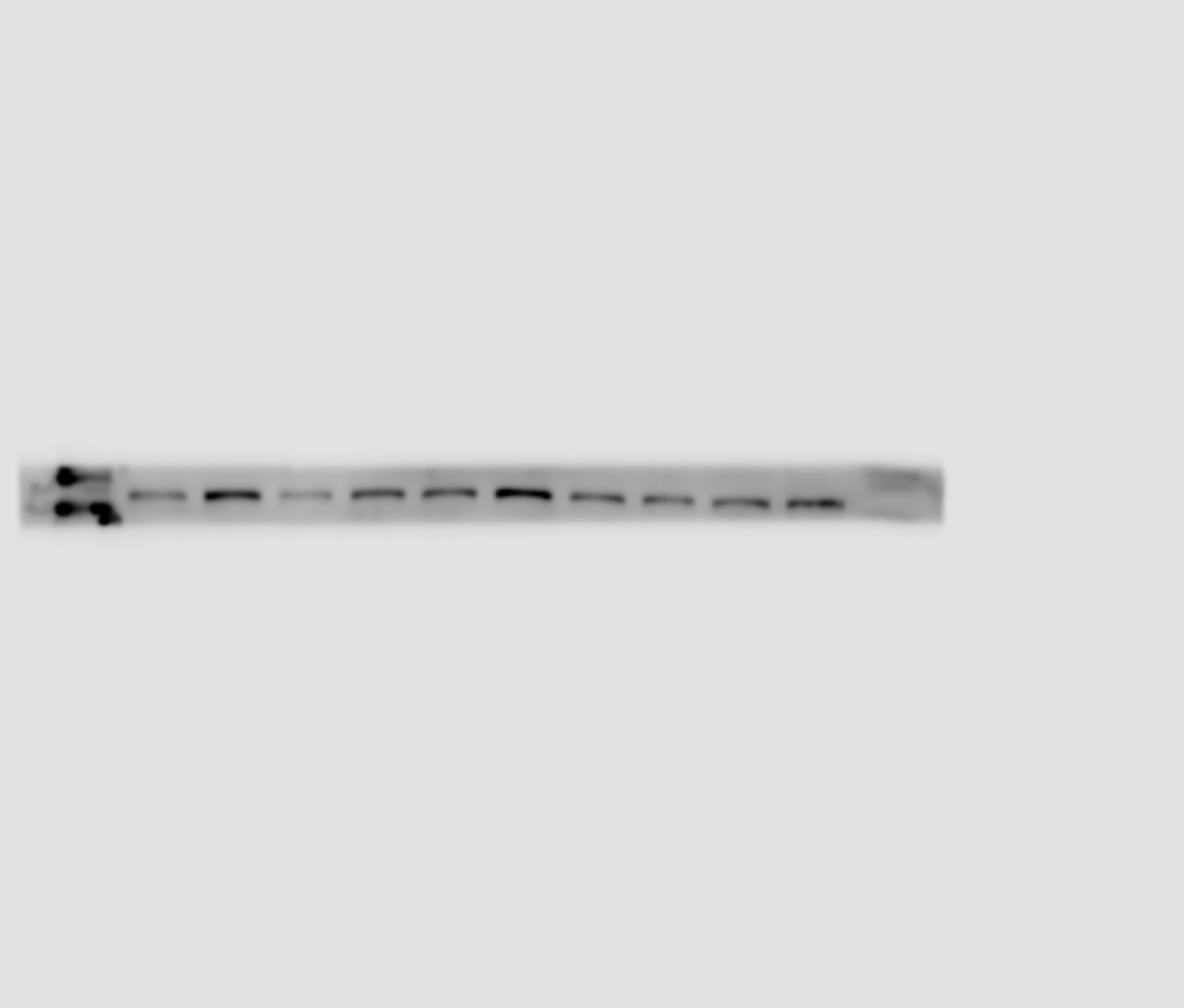

Supplement: FIGURES S1–S5 — File containing all the original uncropped western blot images depicted in the Figures 1(A,B), 2(A–E), 3(A,C–E), 4(A–E), and 5(B–E). [file Data_Sheet_1.ZIP › Figure 2 B/Vps34/Image_0000272_01.tif]

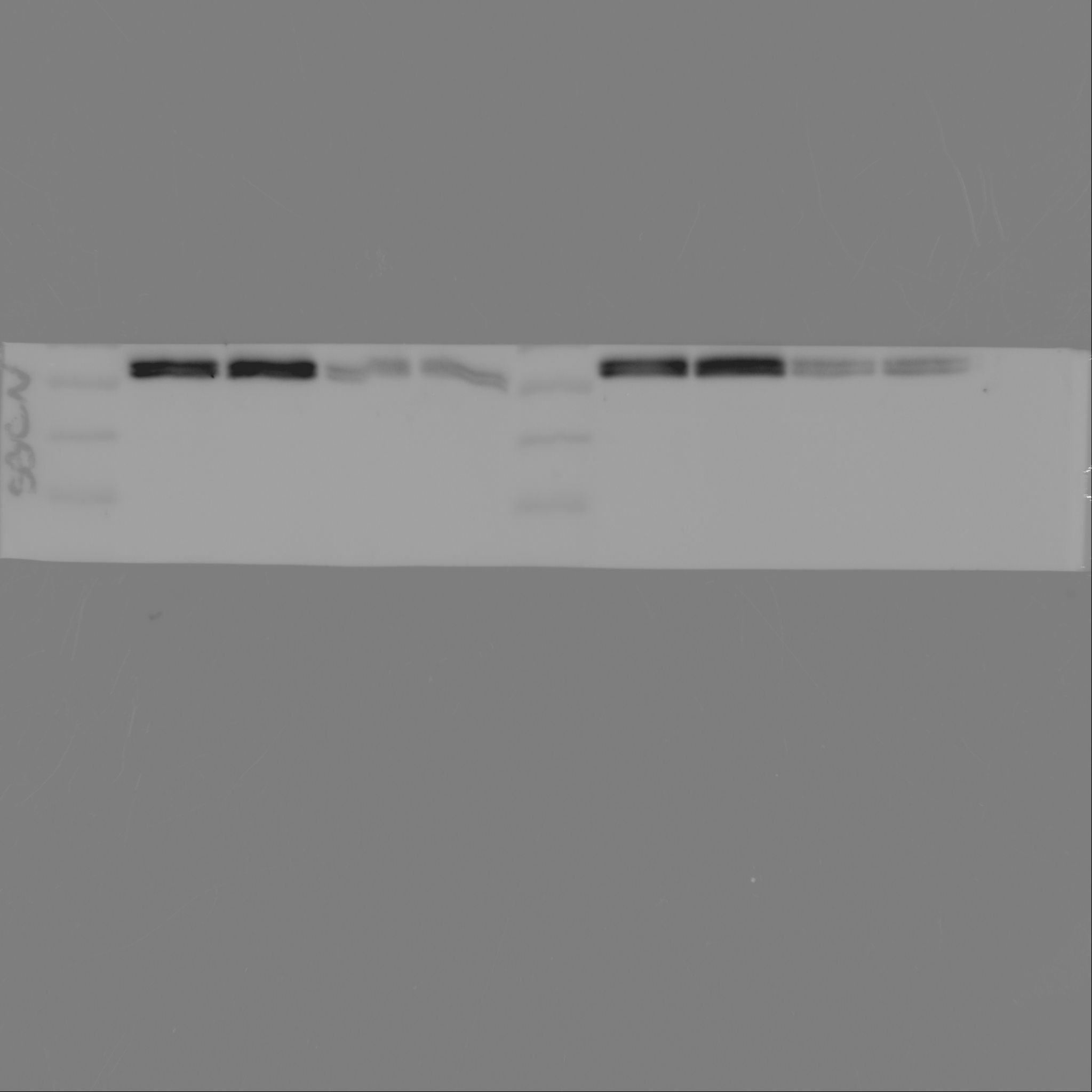

Supplement: FIGURES S1–S5 — File containing all the original uncropped western blot images depicted in the Figures 1(A,B), 2(A–E), 3(A,C–E), 4(A–E), and 5(B–E). [file Data_Sheet_1.ZIP › Figure 2 C/siRNA/Beclin1/image.tif]

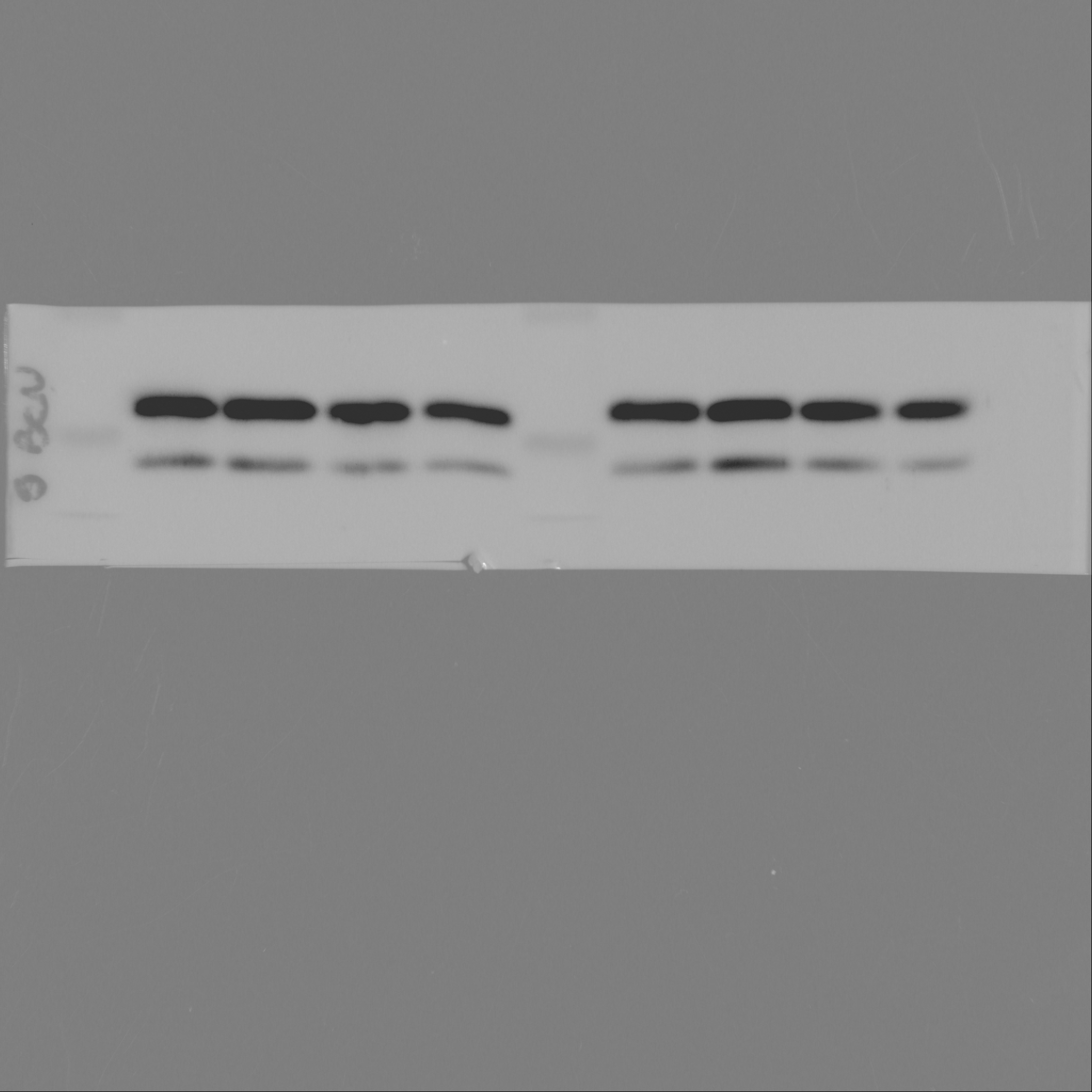

Supplement: FIGURES S1–S5 — File containing all the original uncropped western blot images depicted in the Figures 1(A,B), 2(A–E), 3(A,C–E), 4(A–E), and 5(B–E). [file Data_Sheet_1.ZIP › Figure 2 C/siRNA/LC3/image.tif]

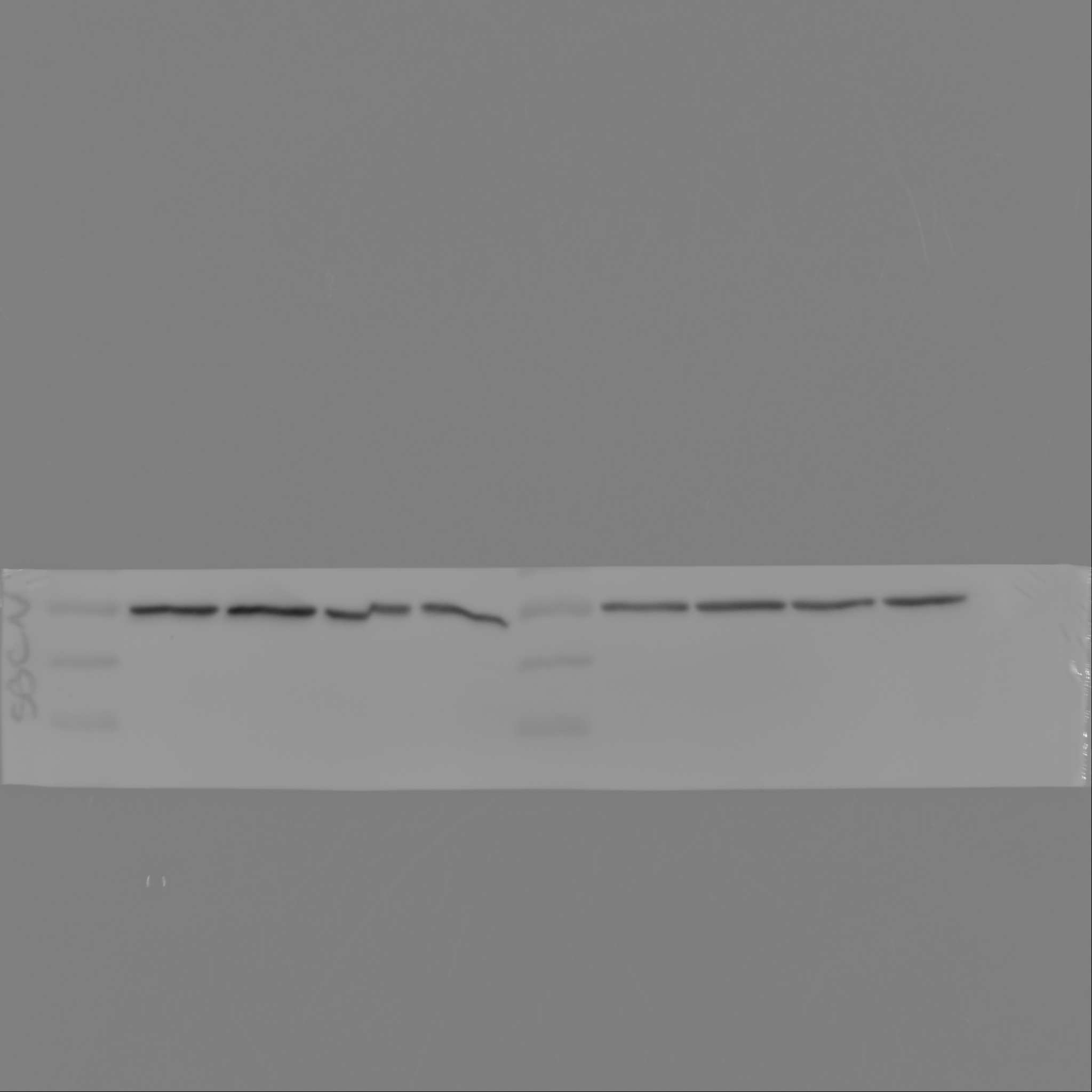

Supplement: FIGURES S1–S5 — File containing all the original uncropped western blot images depicted in the Figures 1(A,B), 2(A–E), 3(A,C–E), 4(A–E), and 5(B–E). [file Data_Sheet_1.ZIP › Figure 2 C/siRNA/Tubulin/image.jpg]

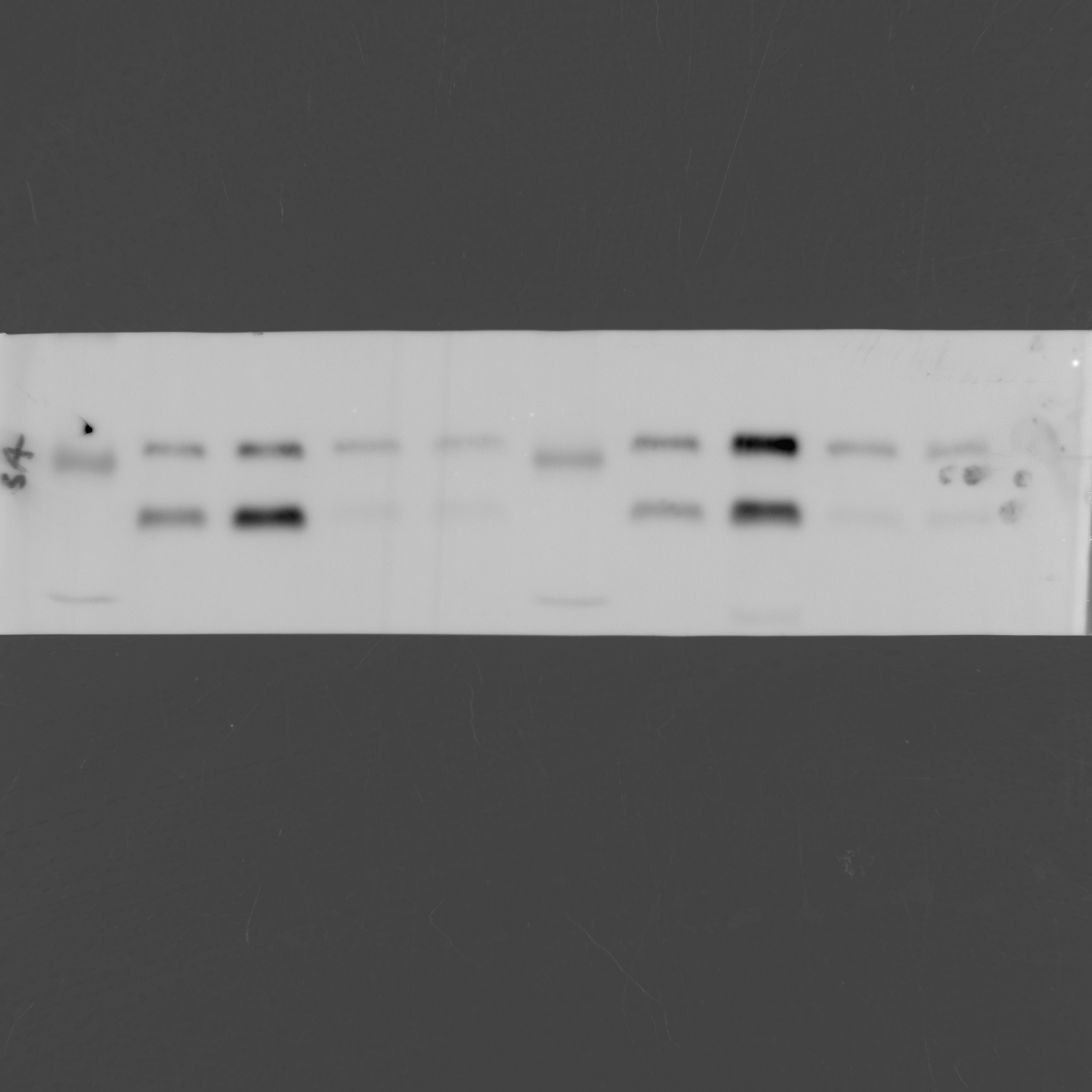

Supplement: FIGURES S1–S5 — File containing all the original uncropped western blot images depicted in the Figures 1(A,B), 2(A–E), 3(A,C–E), 4(A–E), and 5(B–E). [file Data_Sheet_1.ZIP › Figure 2 D/Spautin 1/LC3/image.tif]

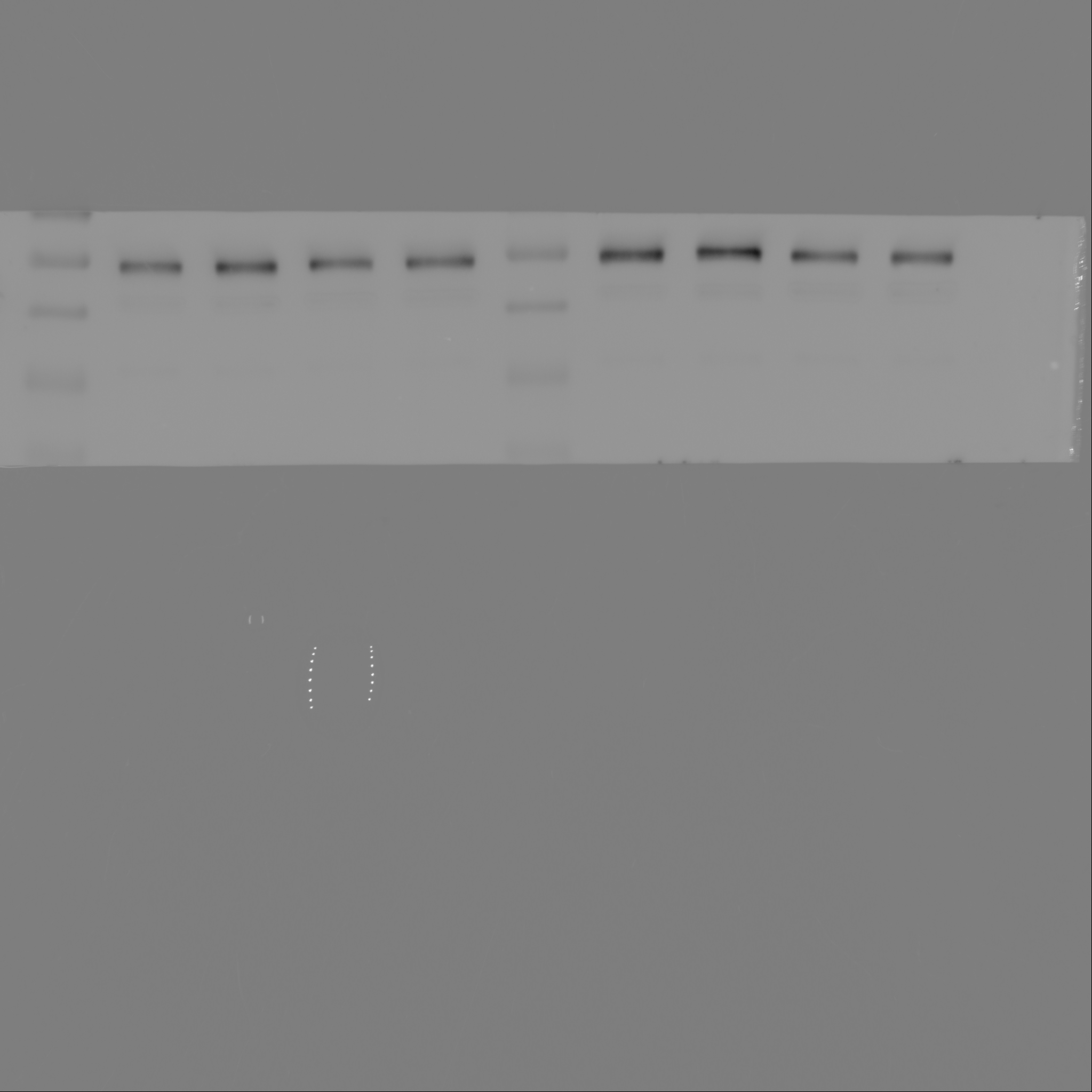

Supplement: FIGURES S1–S5 — File containing all the original uncropped western blot images depicted in the Figures 1(A,B), 2(A–E), 3(A,C–E), 4(A–E), and 5(B–E). [file Data_Sheet_1.ZIP › Figure 2 D/Spautin 1/Tubulin/image.tif]

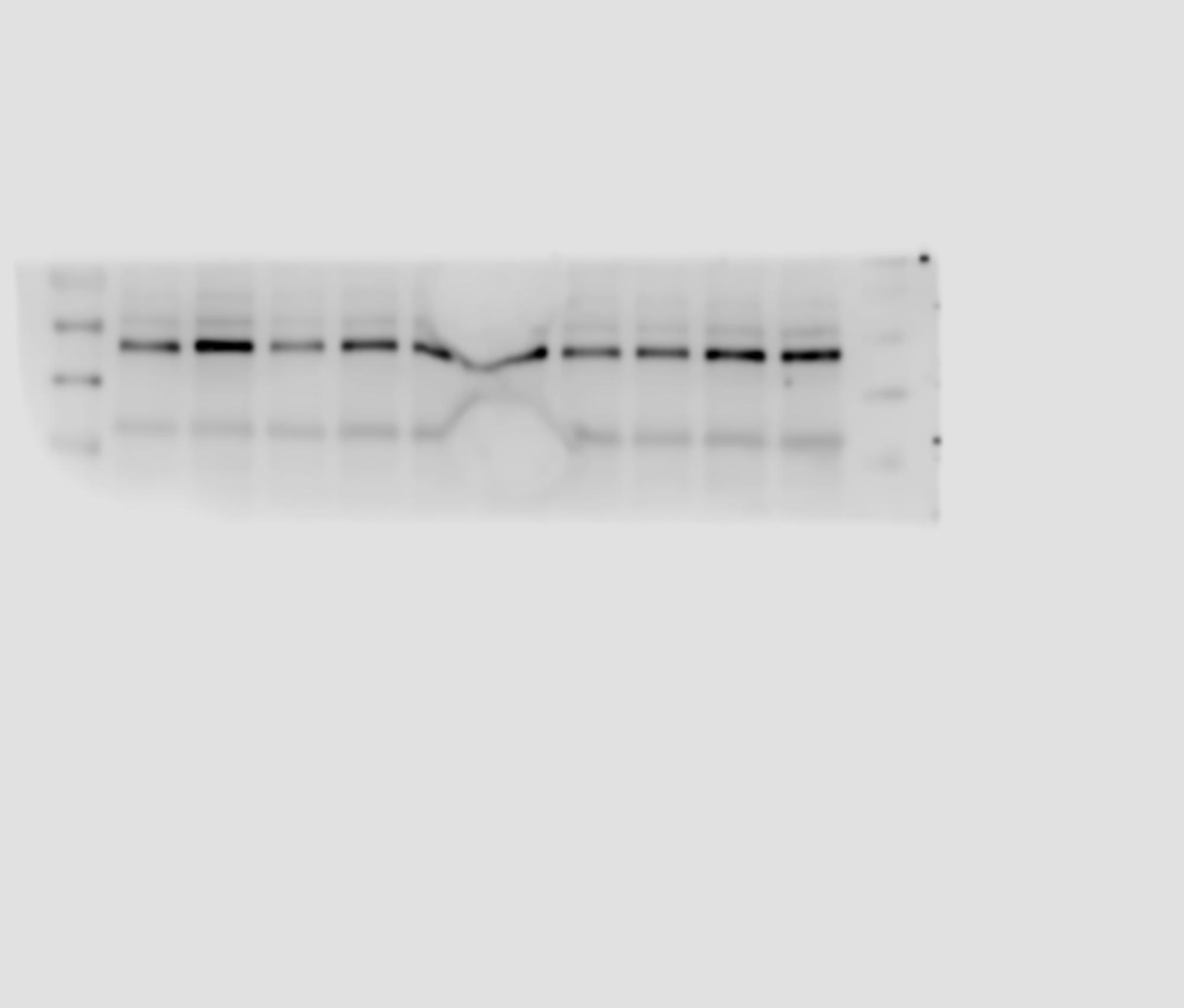

Supplement: FIGURES S1–S5 — File containing all the original uncropped western blot images depicted in the Figures 1(A,B), 2(A–E), 3(A,C–E), 4(A–E), and 5(B–E). [file Data_Sheet_1.ZIP › Figure 2 E/Atg/A7r5/Atg4/Image_0000151_01 1.tif]

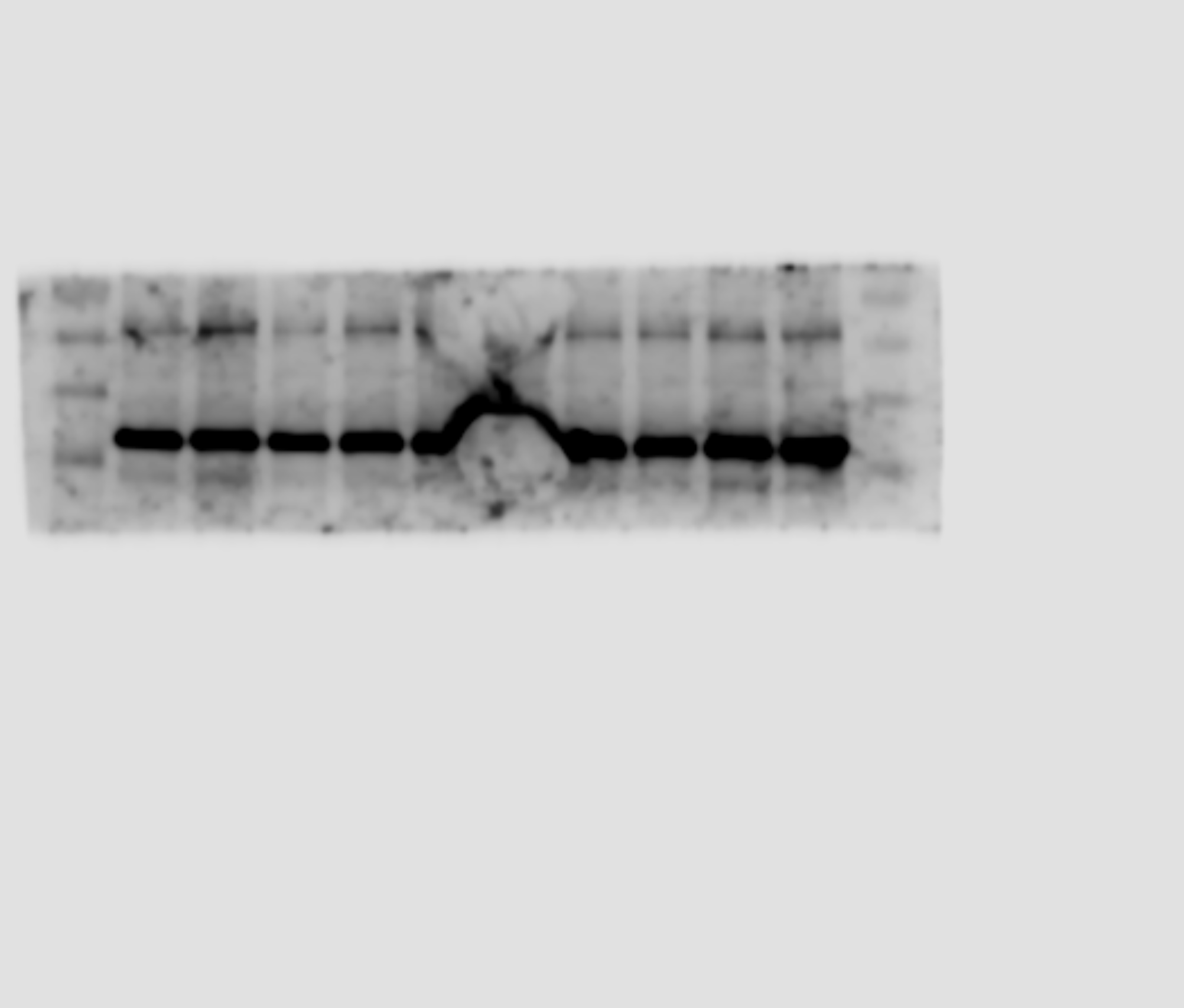

Supplement: FIGURES S1–S5 — File containing all the original uncropped western blot images depicted in the Figures 1(A,B), 2(A–E), 3(A,C–E), 4(A–E), and 5(B–E). [file Data_Sheet_1.ZIP › Figure 2 E/Atg/A7r5/Atg5/Image_0000131_01.tif]

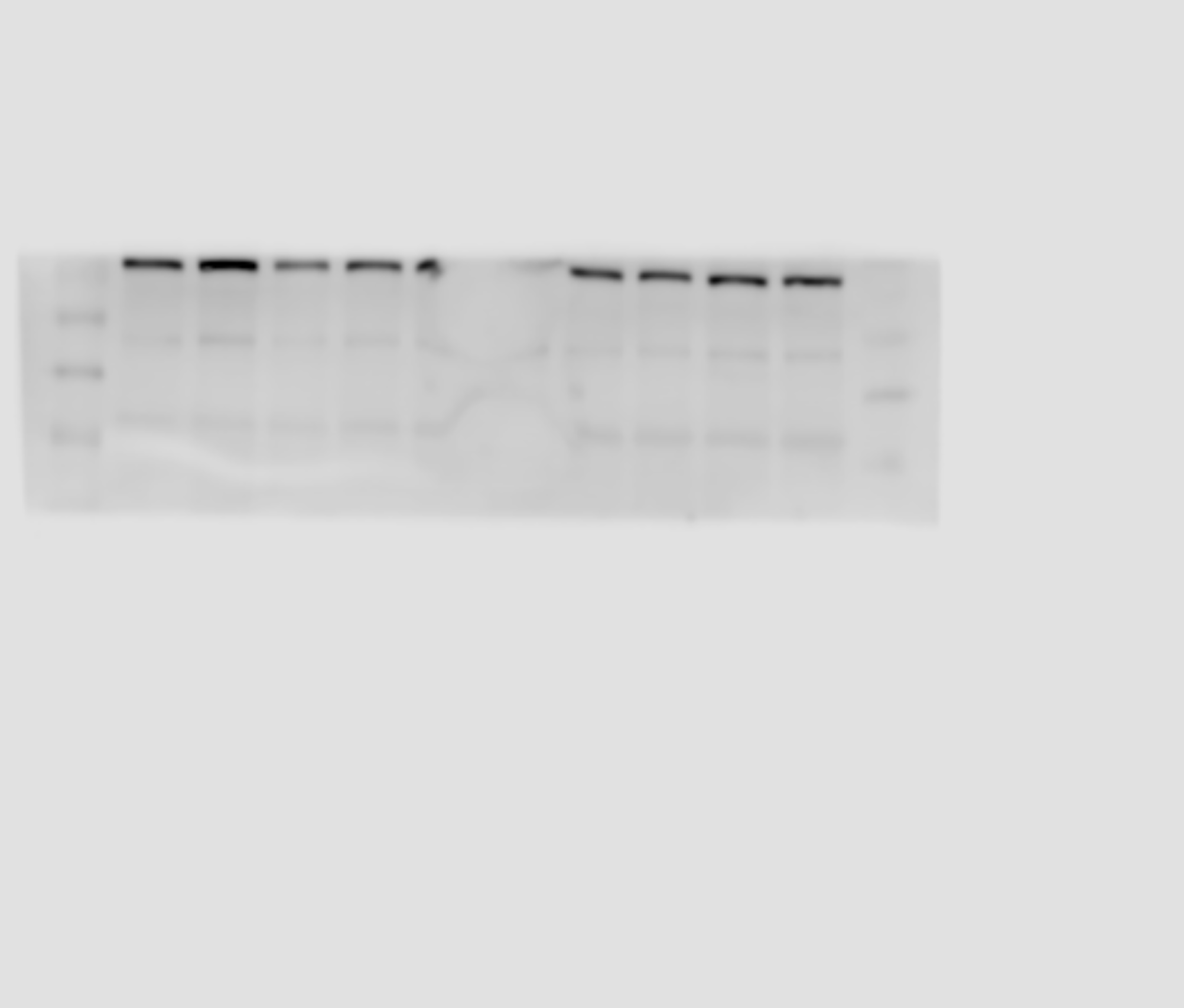

Supplement: FIGURES S1–S5 — File containing all the original uncropped western blot images depicted in the Figures 1(A,B), 2(A–E), 3(A,C–E), 4(A–E), and 5(B–E). [file Data_Sheet_1.ZIP › Figure 2 E/Atg/A7r5/Atg7/Image_0000166_01 1.tif]

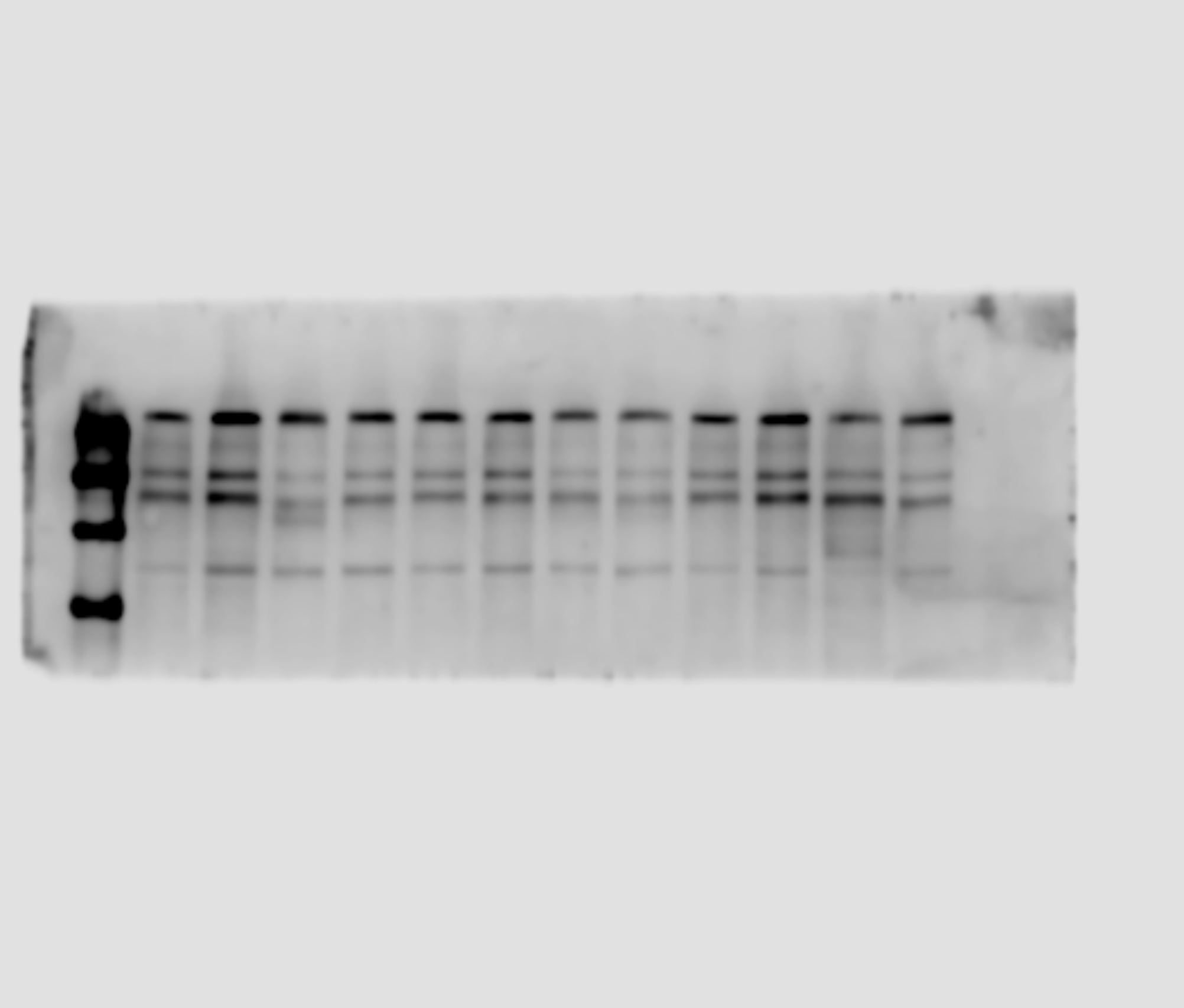

Supplement: FIGURES S1–S5 — File containing all the original uncropped western blot images depicted in the Figures 1(A,B), 2(A–E), 3(A,C–E), 4(A–E), and 5(B–E). [file Data_Sheet_1.ZIP › Figure 2 E/Atg/RASM/Atgs (Atg 7 12-5 and 4)/Image_0000197_01 a.tif]

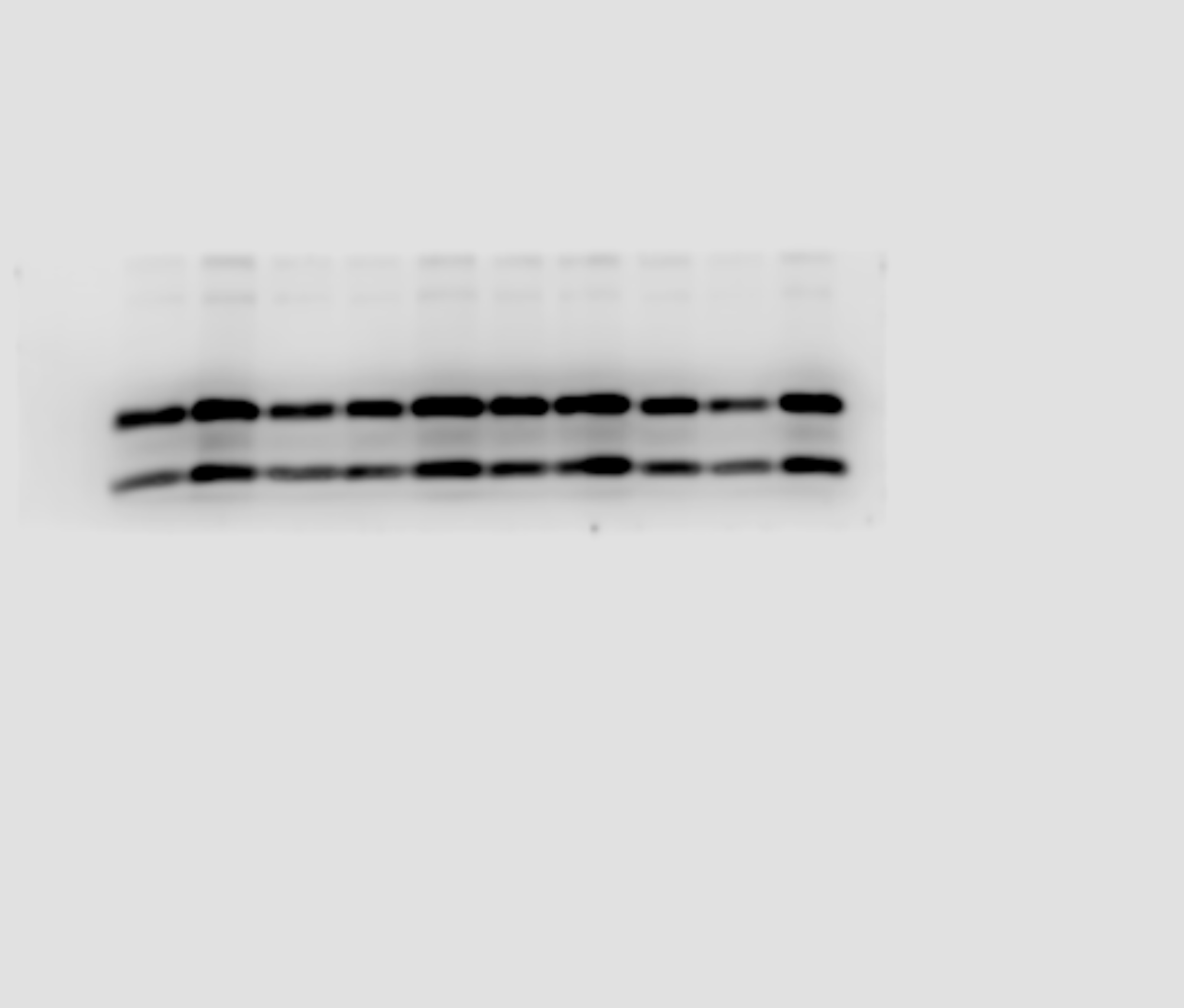

Supplement: FIGURES S1–S5 — File containing all the original uncropped western blot images depicted in the Figures 1(A,B), 2(A–E), 3(A,C–E), 4(A–E), and 5(B–E). [file Data_Sheet_1.ZIP › Figure 3 A/LC3/Image_0000225_01.tif]

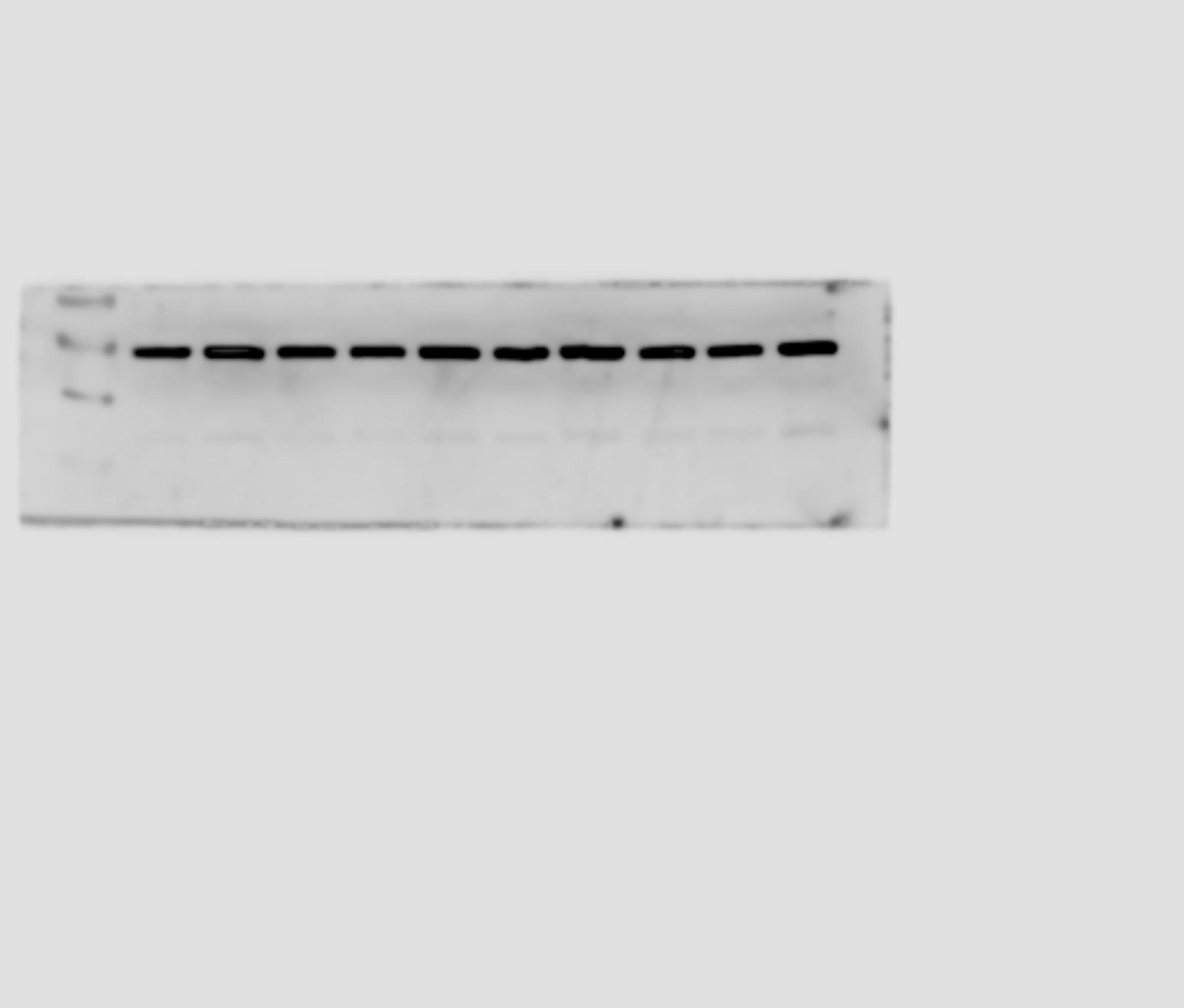

Supplement: FIGURES S1–S5 — File containing all the original uncropped western blot images depicted in the Figures 1(A,B), 2(A–E), 3(A,C–E), 4(A–E), and 5(B–E). [file Data_Sheet_1.ZIP › Figure 3 A/Tubulin/Image_0000293_01.tif]

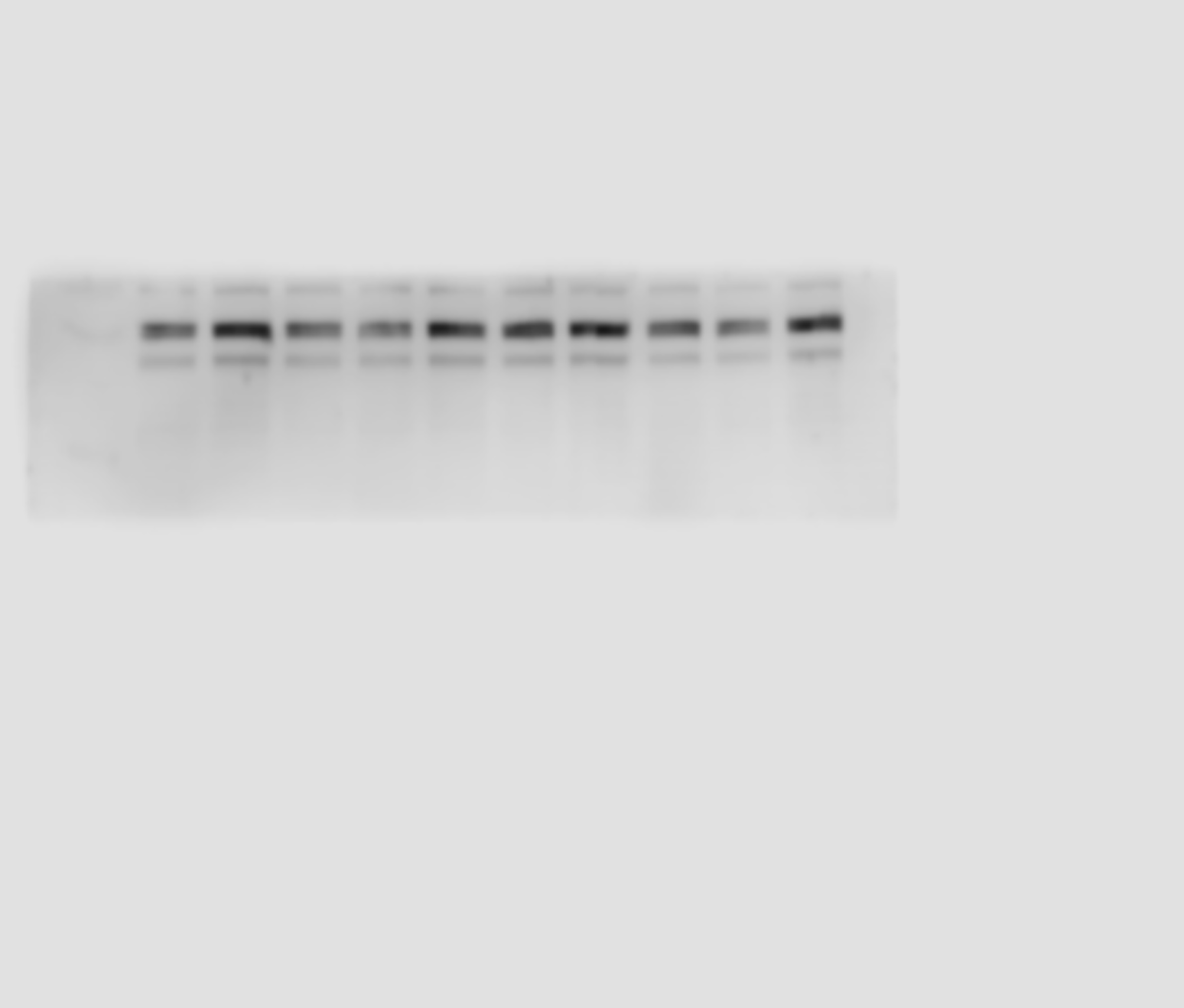

Supplement: FIGURES S1–S5 — File containing all the original uncropped western blot images depicted in the Figures 1(A,B), 2(A–E), 3(A,C–E), 4(A–E), and 5(B–E). [file Data_Sheet_1.ZIP › Figure 3 C/Beclin 1/Beclin1/Image_0000269_01.tif]

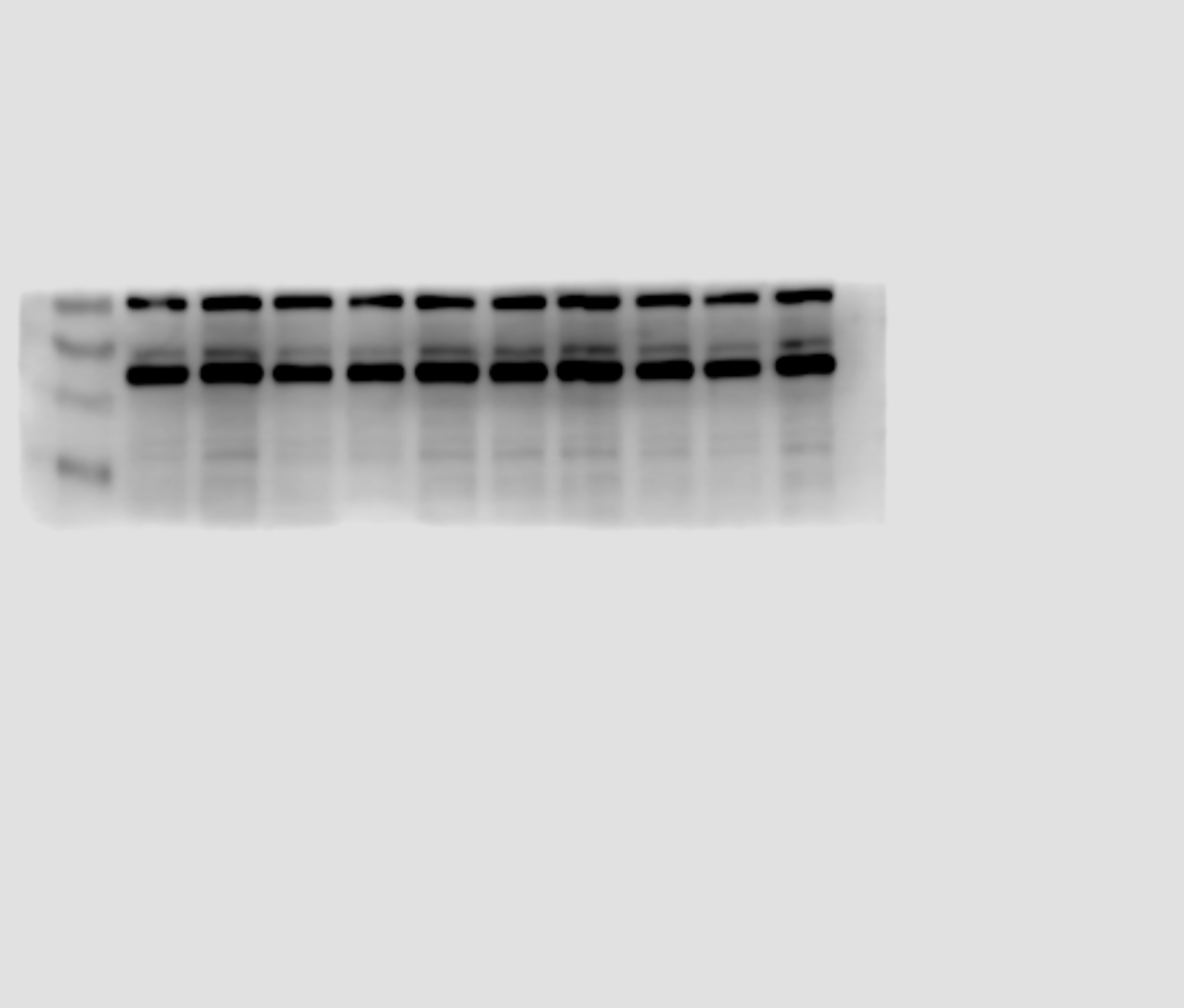

Supplement: FIGURES S1–S5 — File containing all the original uncropped western blot images depicted in the Figures 1(A,B), 2(A–E), 3(A,C–E), 4(A–E), and 5(B–E). [file Data_Sheet_1.ZIP › Figure 3 C/pBeclin1/pBeclin1/Image_0000254_01.tif]

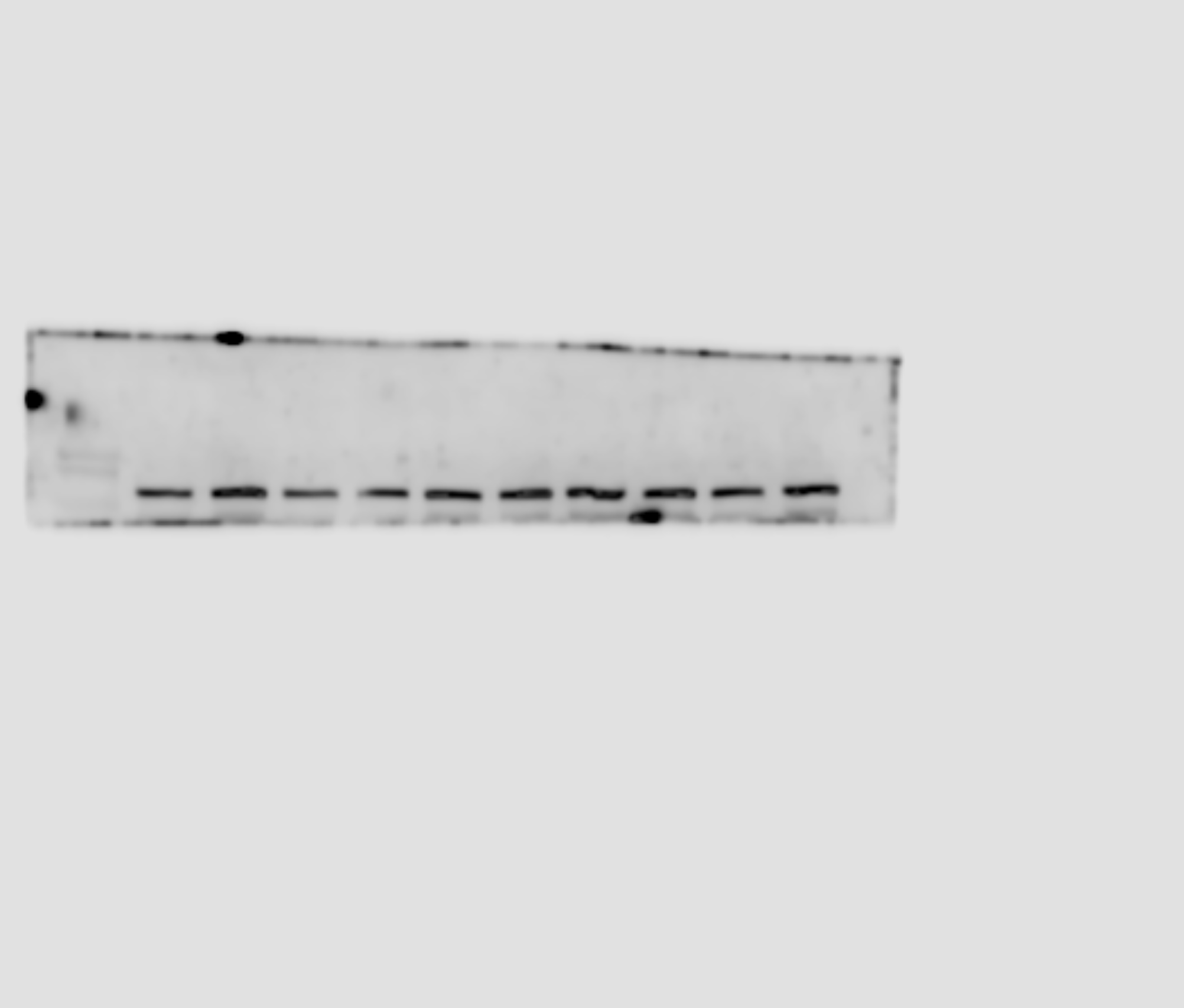

Supplement: FIGURES S1–S5 — File containing all the original uncropped western blot images depicted in the Figures 1(A,B), 2(A–E), 3(A,C–E), 4(A–E), and 5(B–E). [file Data_Sheet_1.ZIP › Figure 3 D/Vps34/Image_0000280_01.tif]

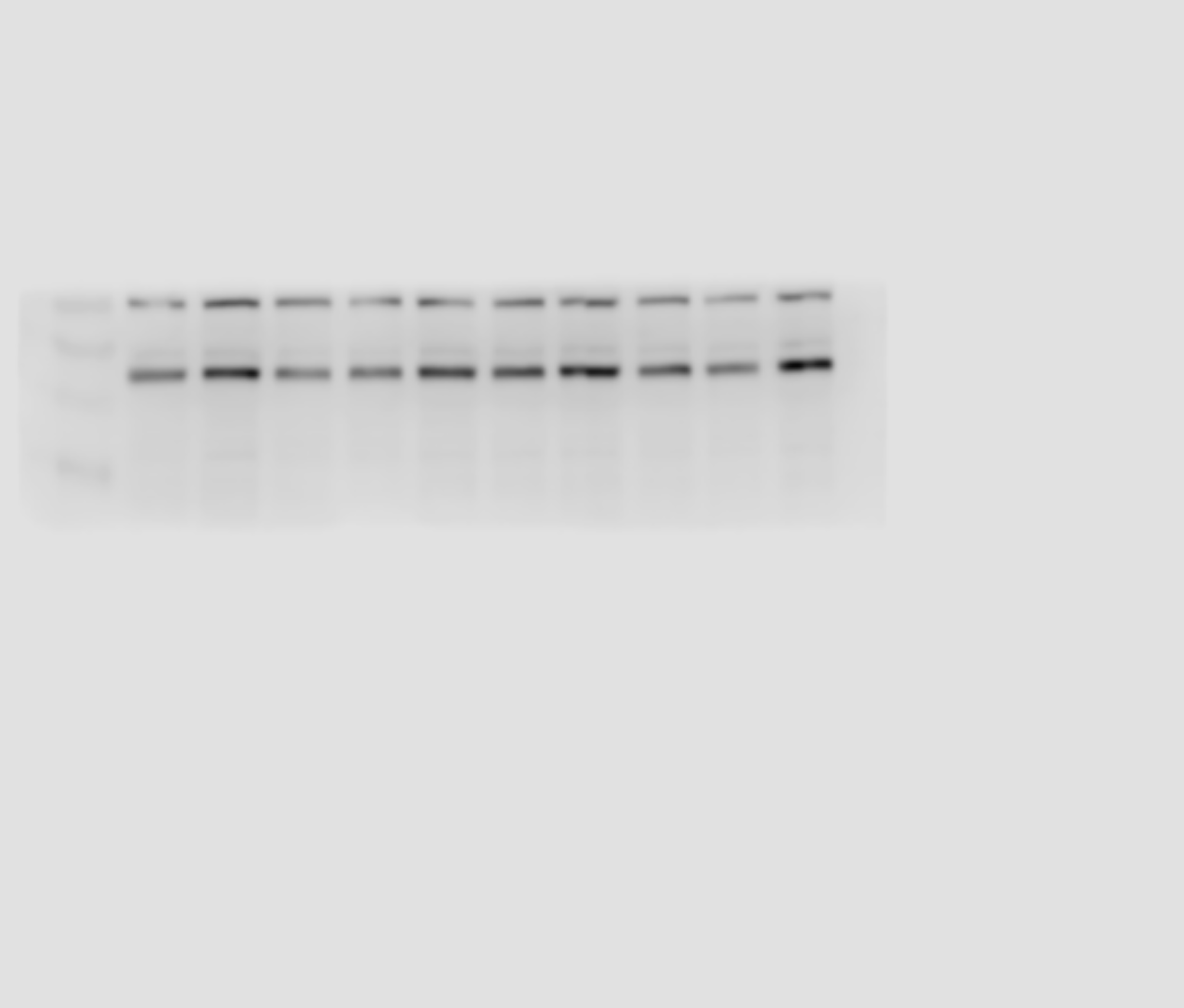

Supplement: FIGURES S1–S5 — File containing all the original uncropped western blot images depicted in the Figures 1(A,B), 2(A–E), 3(A,C–E), 4(A–E), and 5(B–E). [file Data_Sheet_1.ZIP › Figure 3 E/Atg4/Image_0000254_01.tif]

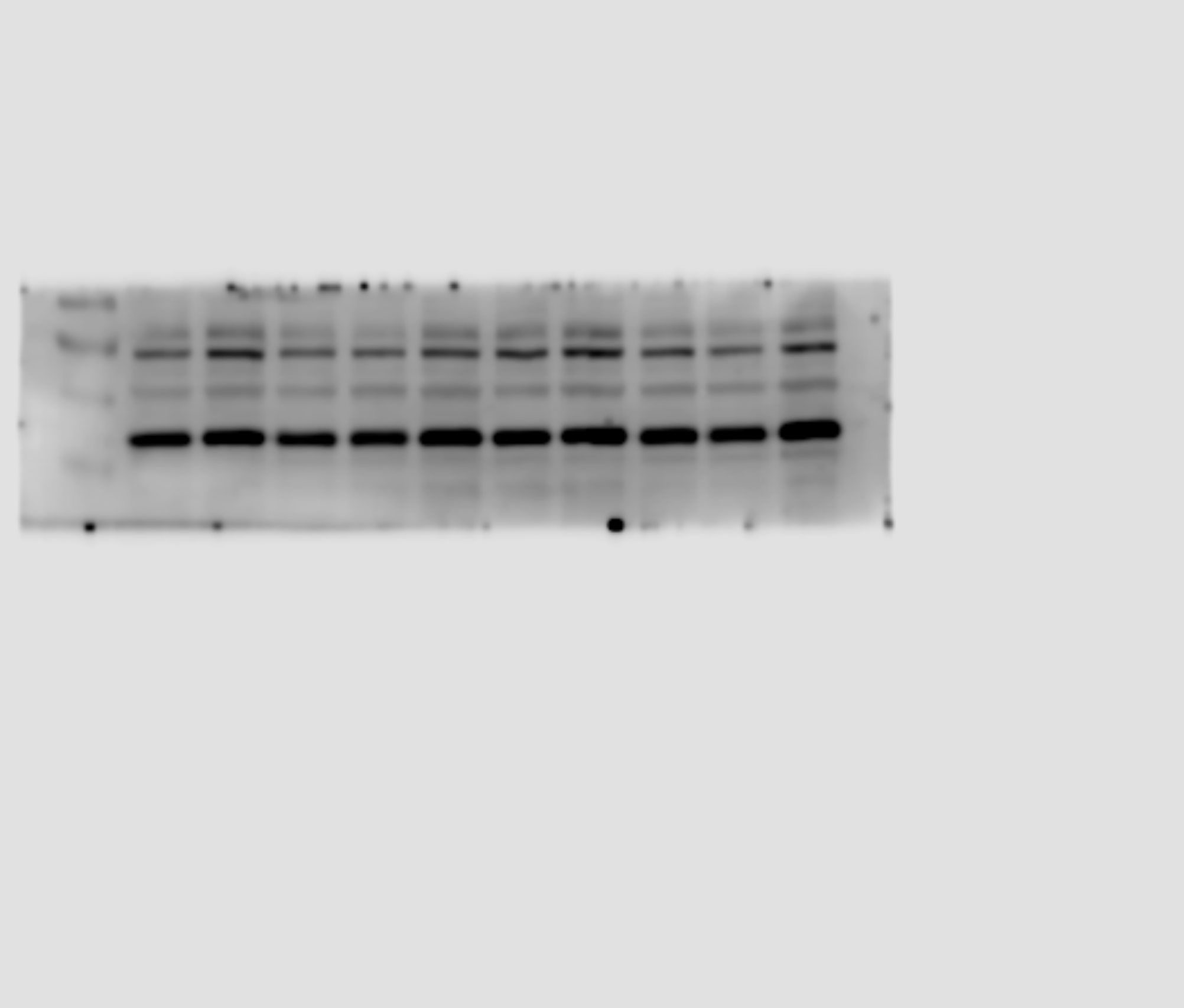

Supplement: FIGURES S1–S5 — File containing all the original uncropped western blot images depicted in the Figures 1(A,B), 2(A–E), 3(A,C–E), 4(A–E), and 5(B–E). [file Data_Sheet_1.ZIP › Figure 3 E/Atg5/Image_0000297_01.tif]

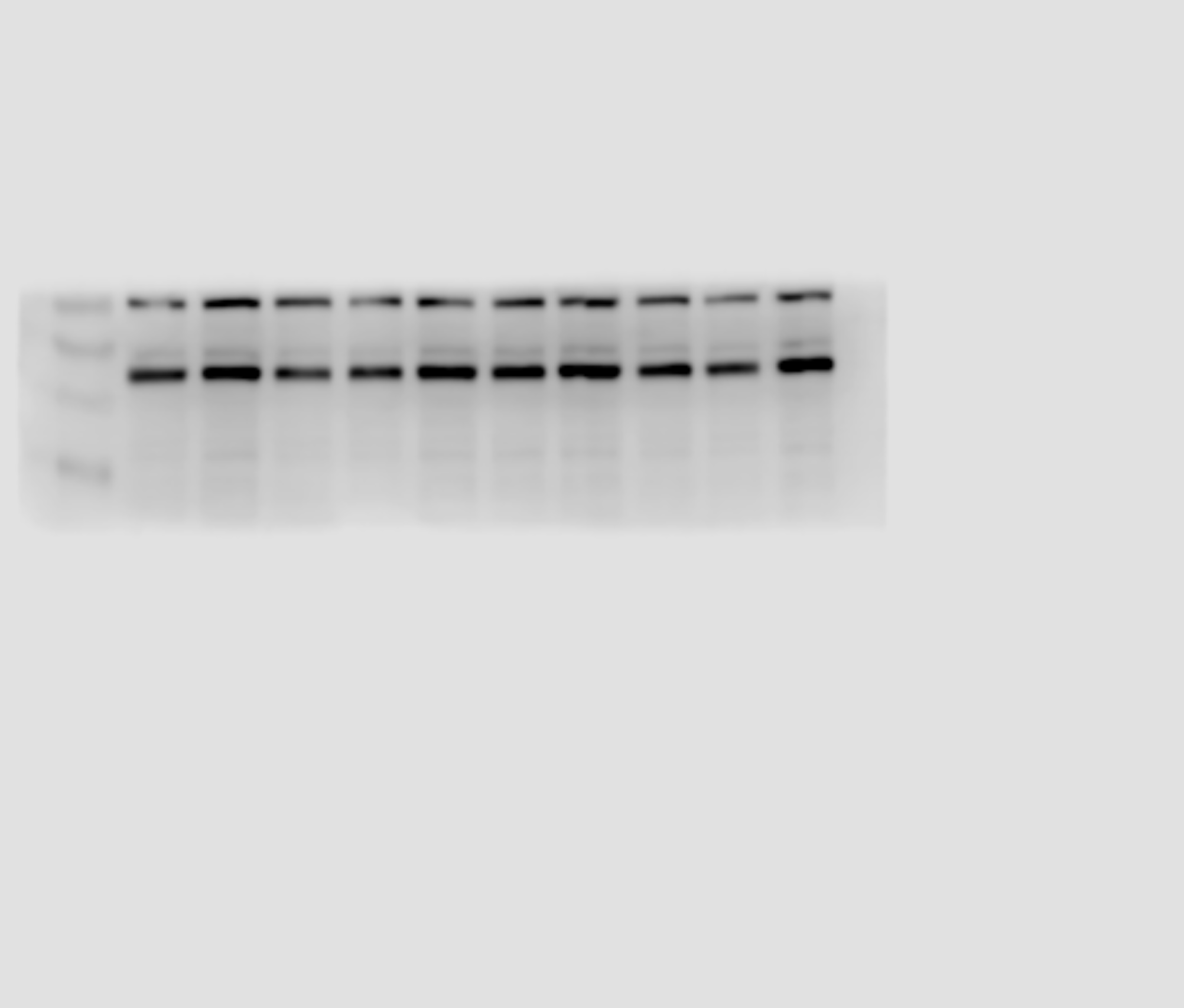

Supplement: FIGURES S1–S5 — File containing all the original uncropped western blot images depicted in the Figures 1(A,B), 2(A–E), 3(A,C–E), 4(A–E), and 5(B–E). [file Data_Sheet_1.ZIP › Figure 3 E/Atg7/Image_0000254_01.tif]

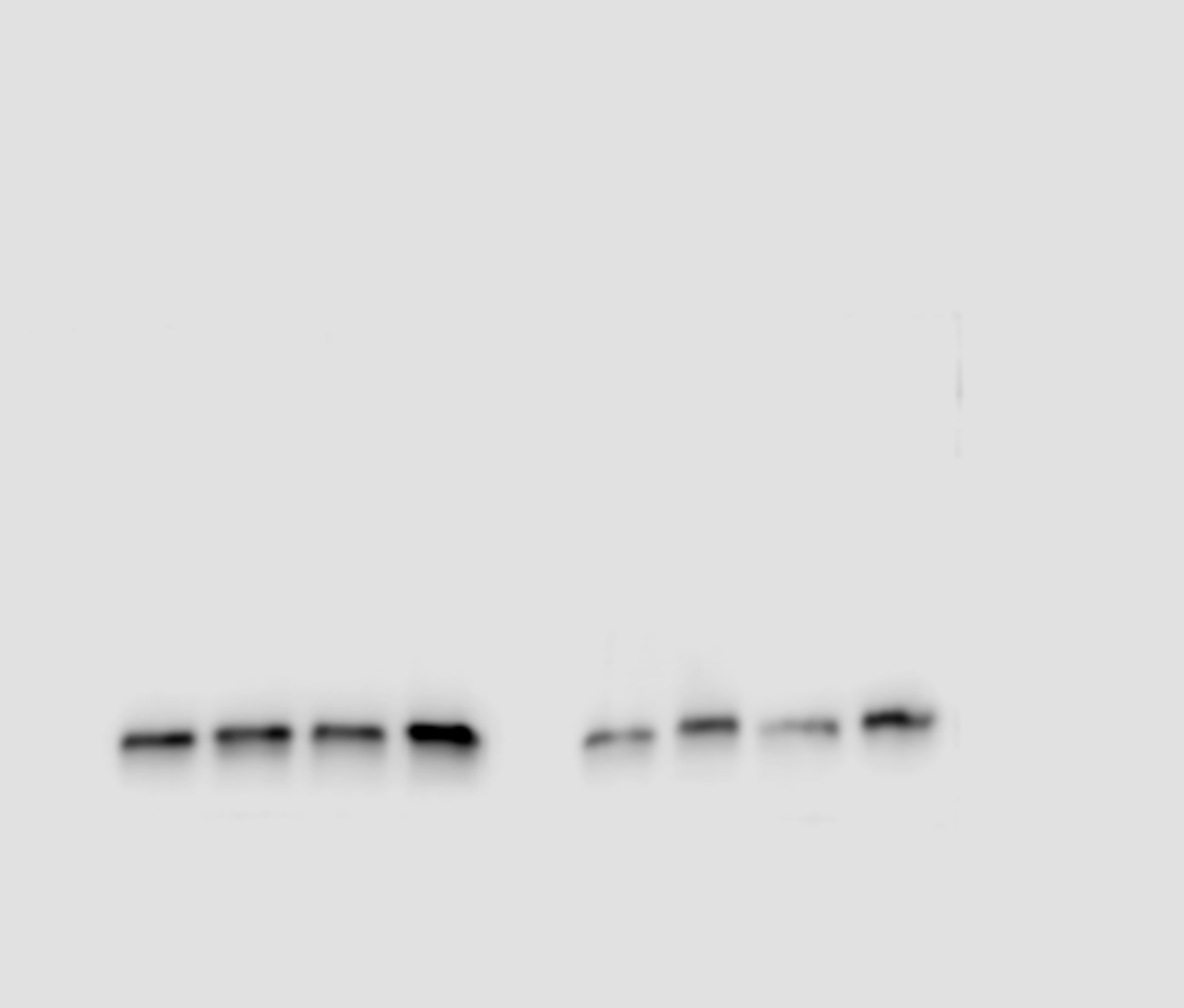

Supplement: FIGURES S1–S5 — File containing all the original uncropped western blot images depicted in the Figures 1(A,B), 2(A–E), 3(A,C–E), 4(A–E), and 5(B–E). [file Data_Sheet_1.ZIP › Figure 4 A/p4EBP1/Image_0000212_01.tif]

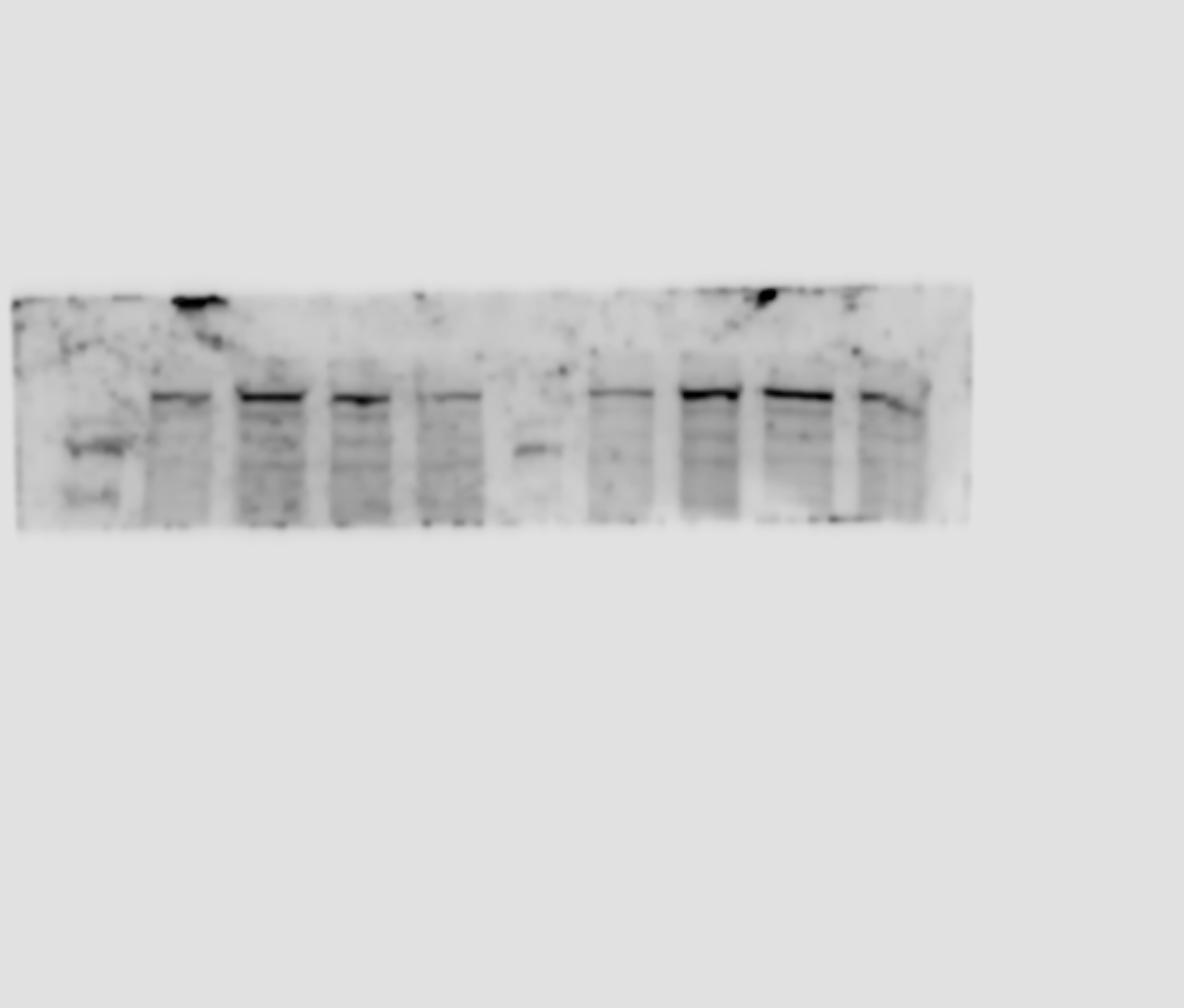

Supplement: FIGURES S1–S5 — File containing all the original uncropped western blot images depicted in the Figures 1(A,B), 2(A–E), 3(A,C–E), 4(A–E), and 5(B–E). [file Data_Sheet_1.ZIP › Figure 4 A/pmTOR/Image_0000129_01.tif]

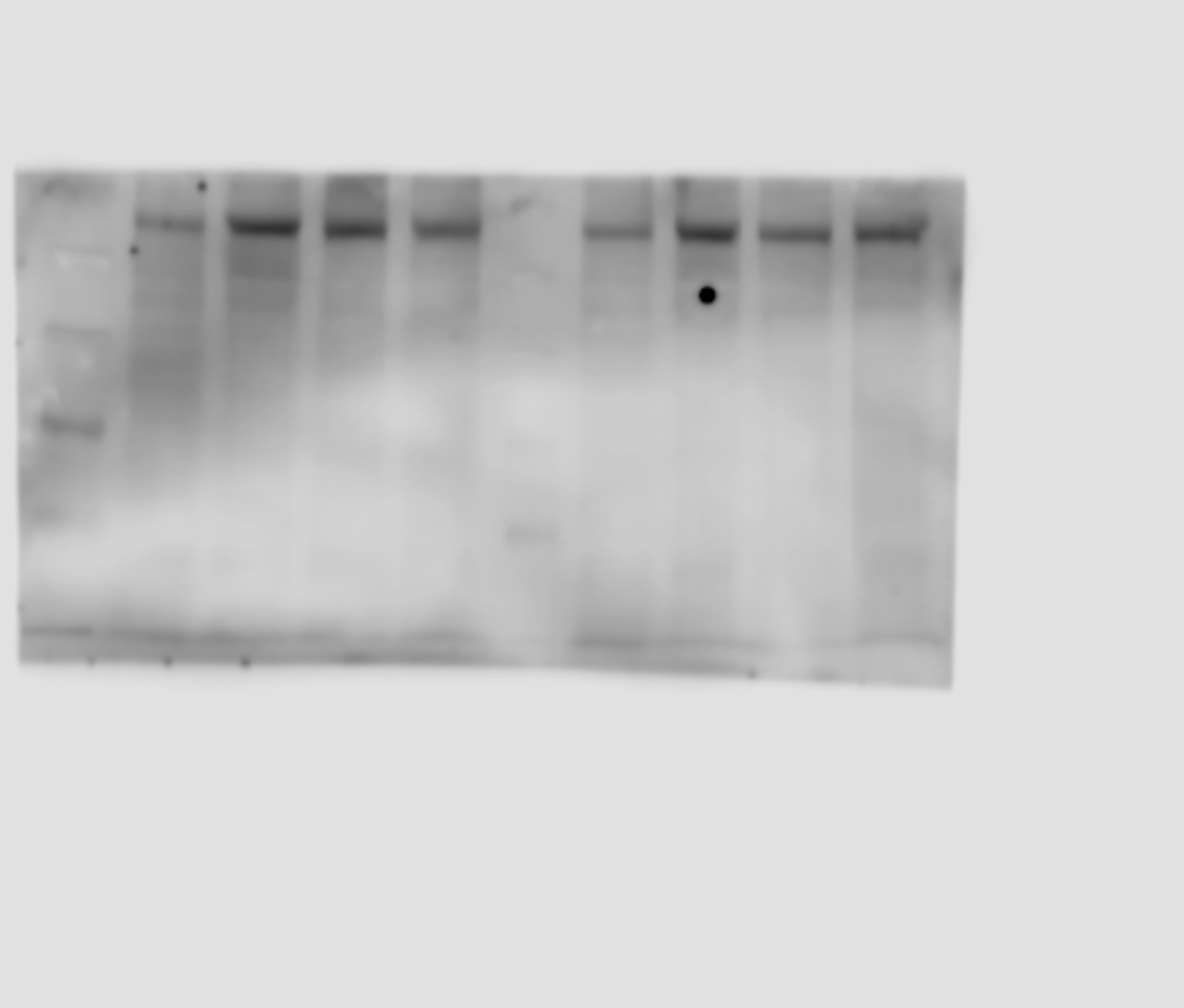

Supplement: FIGURES S1–S5 — File containing all the original uncropped western blot images depicted in the Figures 1(A,B), 2(A–E), 3(A,C–E), 4(A–E), and 5(B–E). [file Data_Sheet_1.ZIP › Figure 4 A/pp70s6k1/Image_0000171_01.tif]

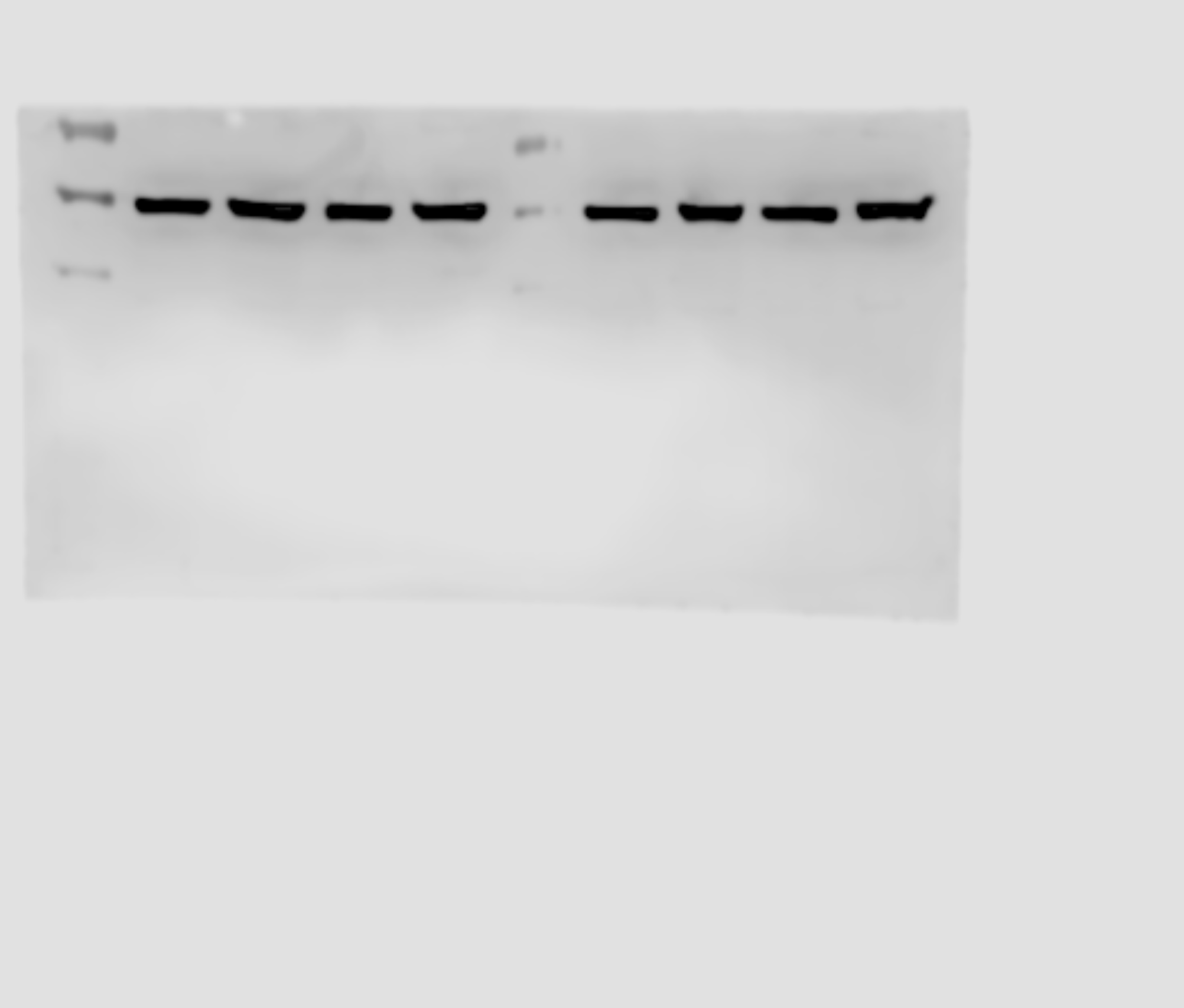

Supplement: FIGURES S1–S5 — File containing all the original uncropped western blot images depicted in the Figures 1(A,B), 2(A–E), 3(A,C–E), 4(A–E), and 5(B–E). [file Data_Sheet_1.ZIP › Figure 4 A/Tubulin/Image_0000185_01.tif]

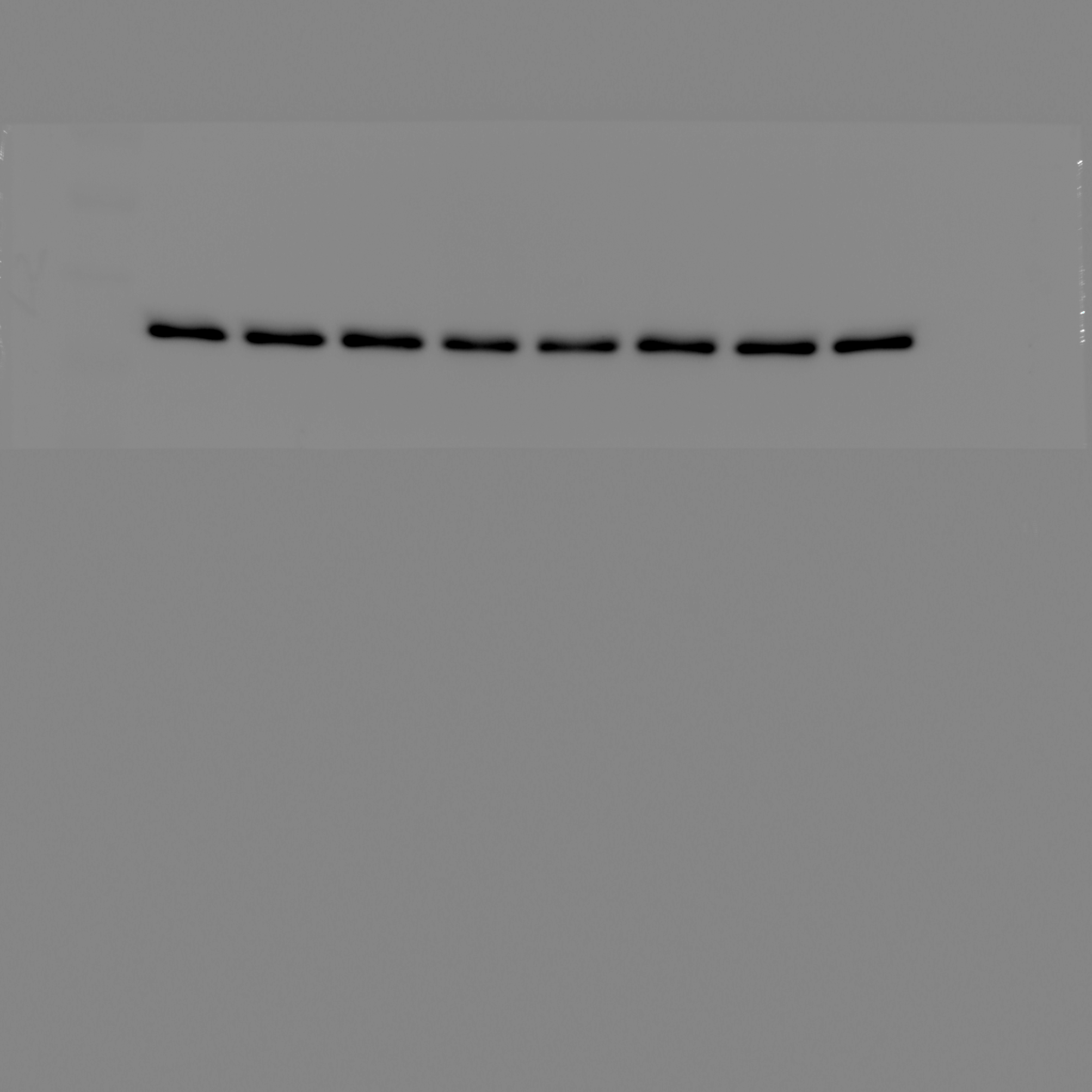

Supplement: FIGURES S1–S5 — File containing all the original uncropped western blot images depicted in the Figures 1(A,B), 2(A–E), 3(A,C–E), 4(A–E), and 5(B–E). [file Data_Sheet_1.ZIP › Figure 4 B/GAPDH/Image_0000263_01.tif]

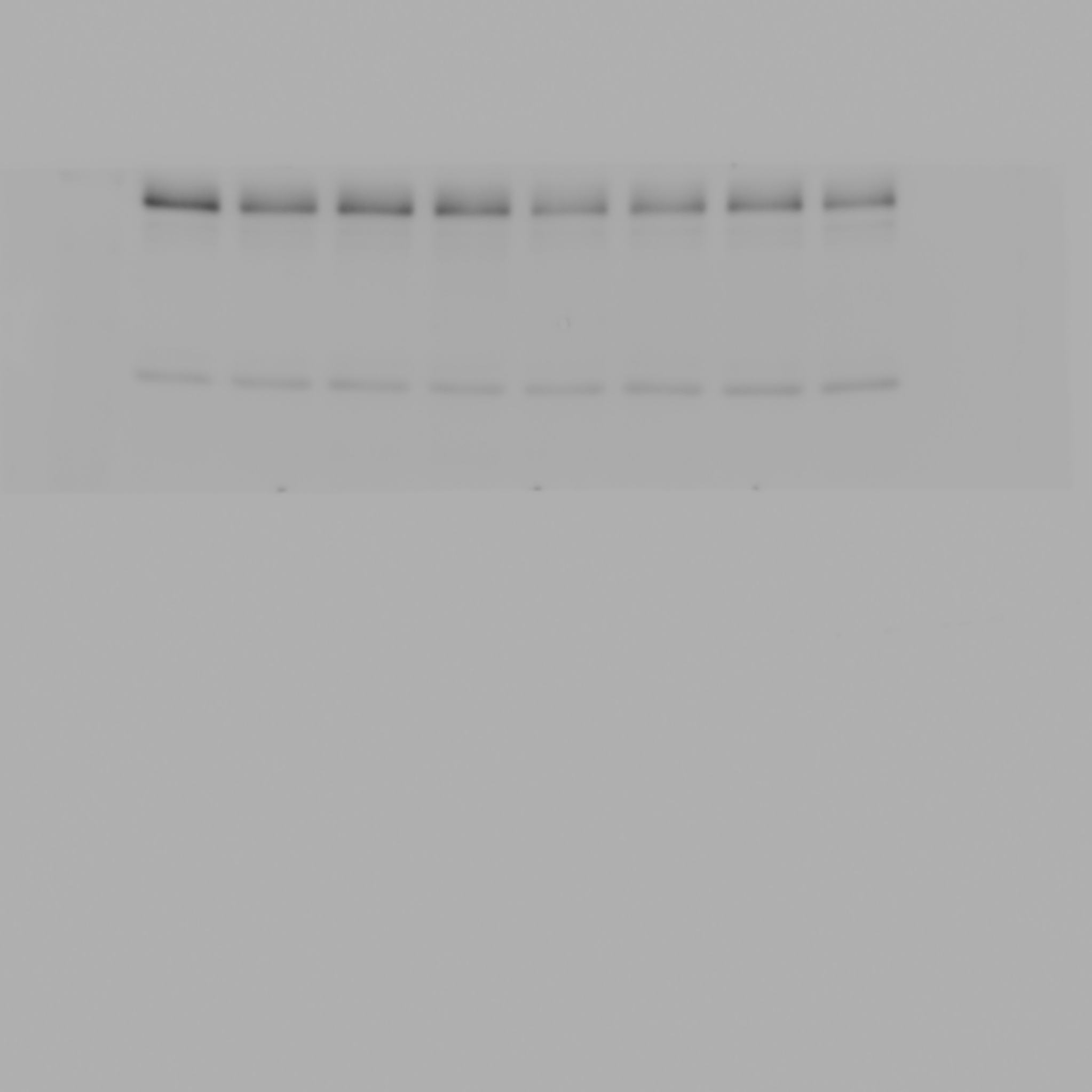

Supplement: FIGURES S1–S5 — File containing all the original uncropped western blot images depicted in the Figures 1(A,B), 2(A–E), 3(A,C–E), 4(A–E), and 5(B–E). [file Data_Sheet_1.ZIP › Figure 4 B/pAMPK/image.tif]

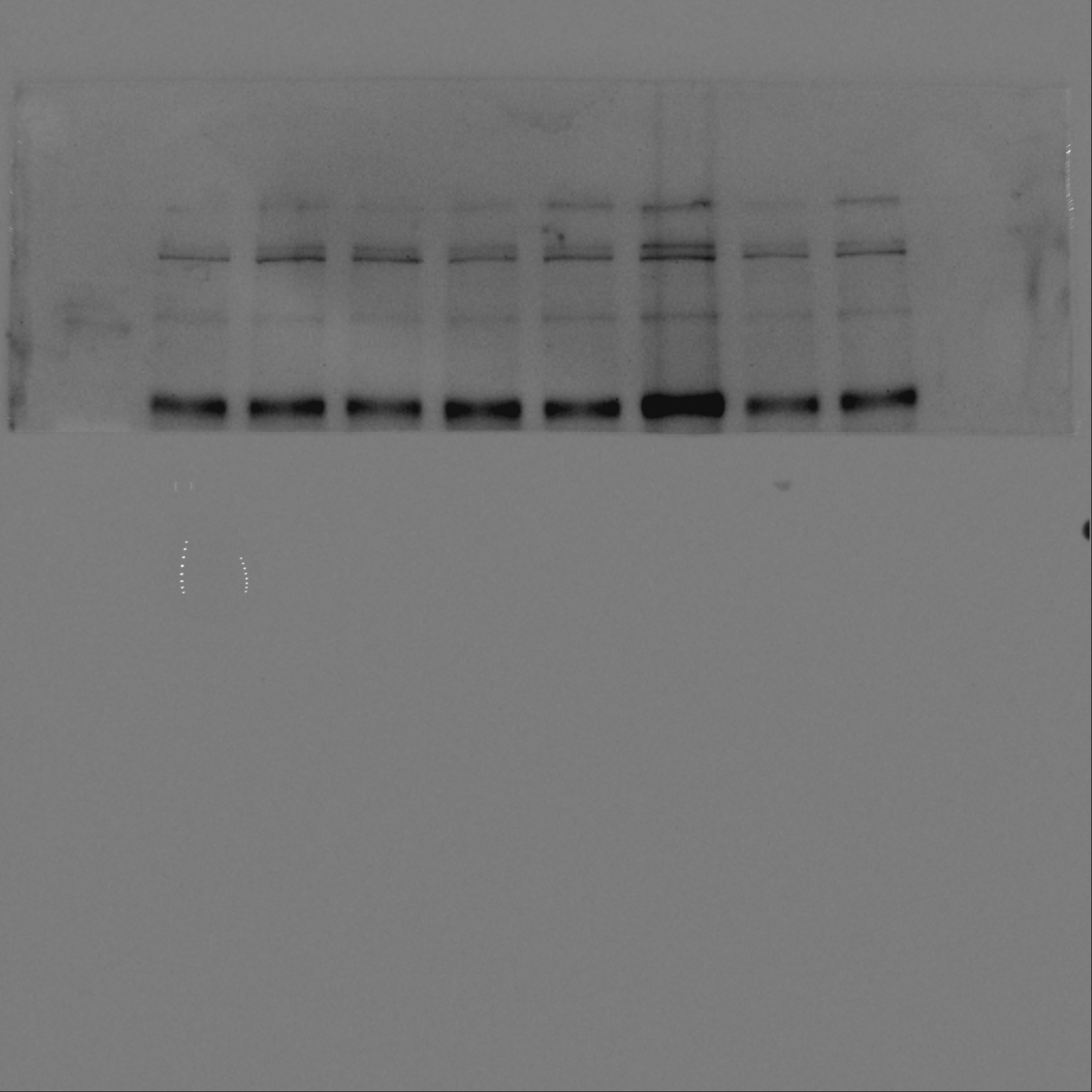

Supplement: FIGURES S1–S5 — File containing all the original uncropped western blot images depicted in the Figures 1(A,B), 2(A–E), 3(A,C–E), 4(A–E), and 5(B–E). [file Data_Sheet_1.ZIP › Figure 4 B/pTSC2/image.tif]

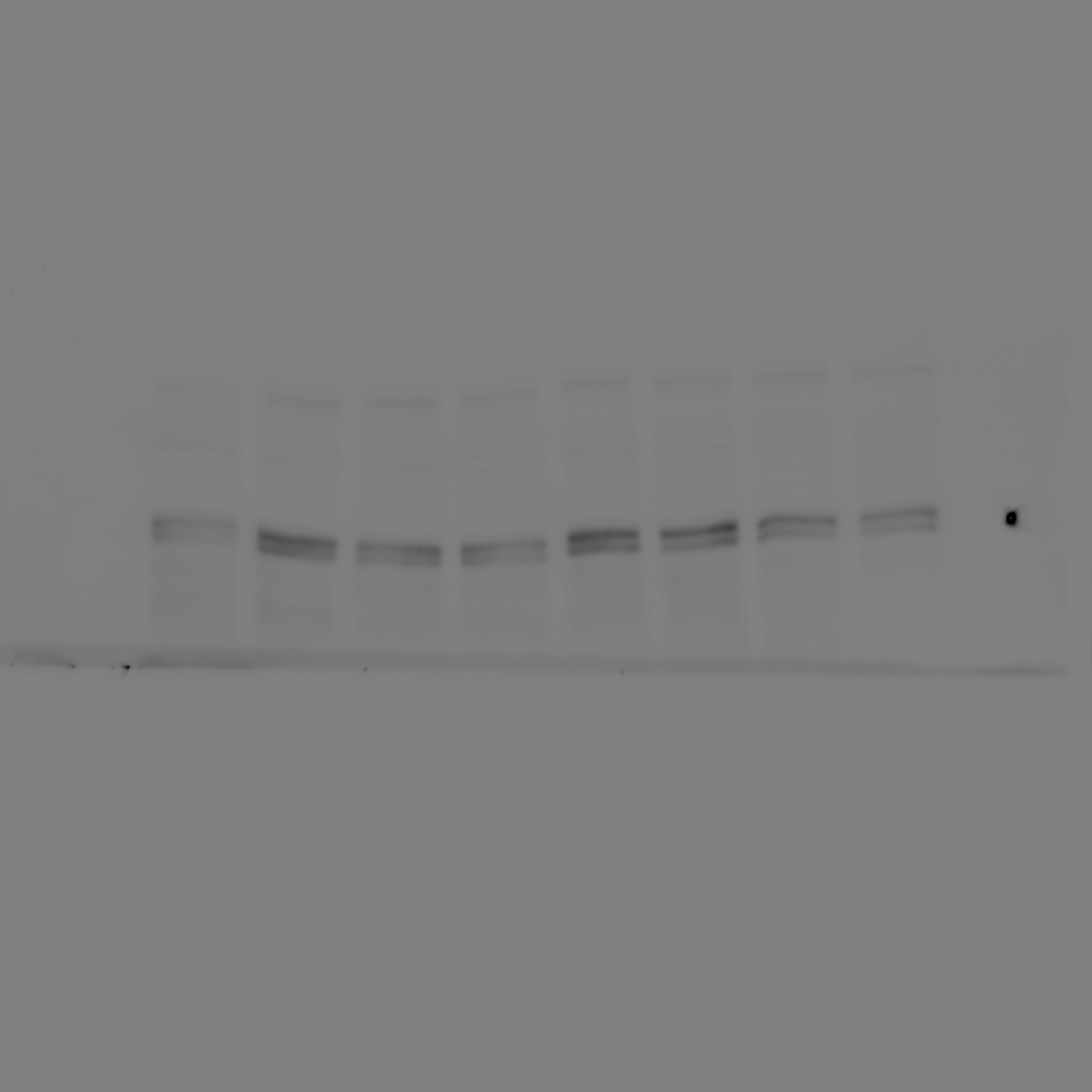

Supplement: FIGURES S1–S5 — File containing all the original uncropped western blot images depicted in the Figures 1(A,B), 2(A–E), 3(A,C–E), 4(A–E), and 5(B–E). [file Data_Sheet_1.ZIP › Figure 4 C/MYPT1/image.tif]

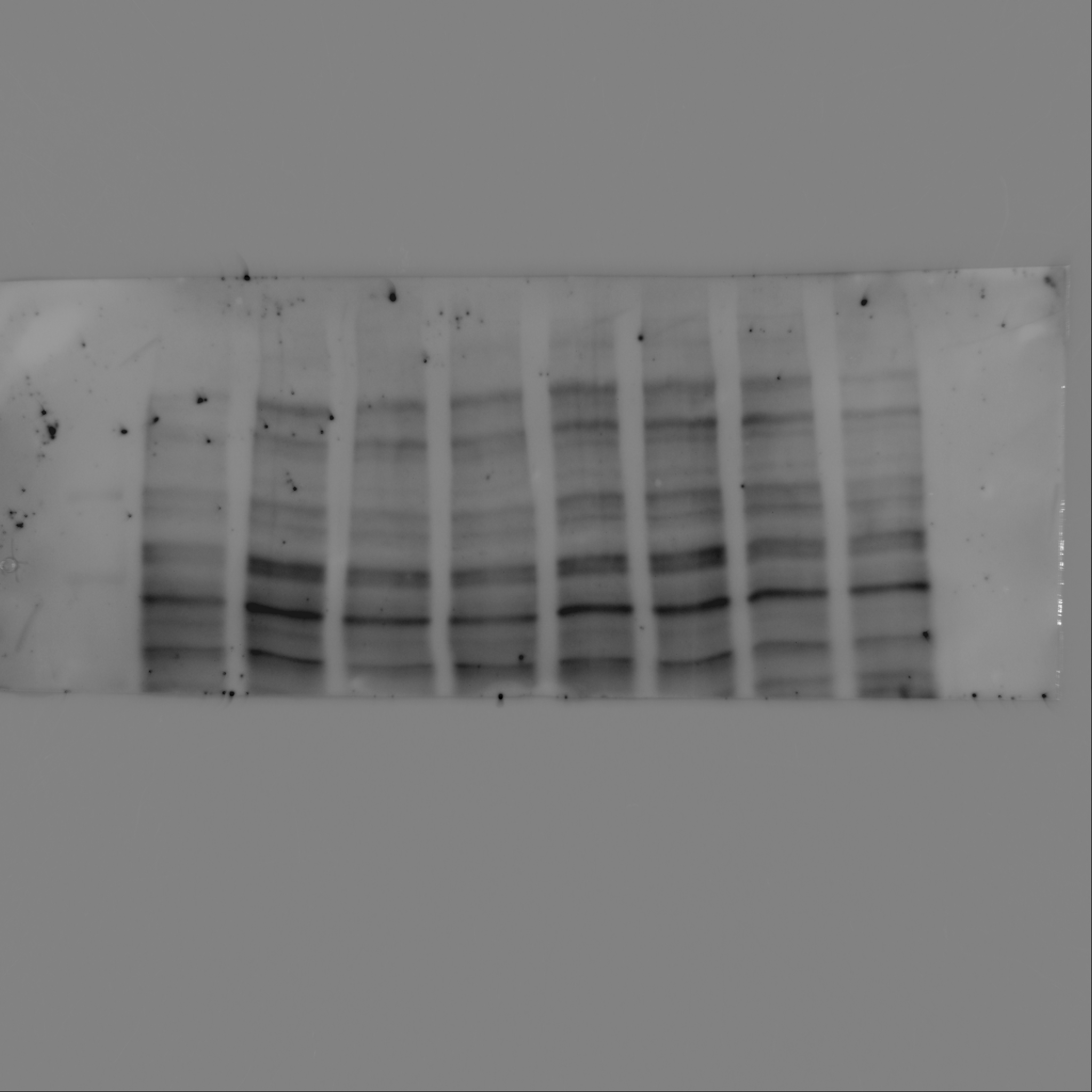

Supplement: FIGURES S1–S5 — File containing all the original uncropped western blot images depicted in the Figures 1(A,B), 2(A–E), 3(A,C–E), 4(A–E), and 5(B–E). [file Data_Sheet_1.ZIP › Figure 4 C/pMYPT1/image.jpg]

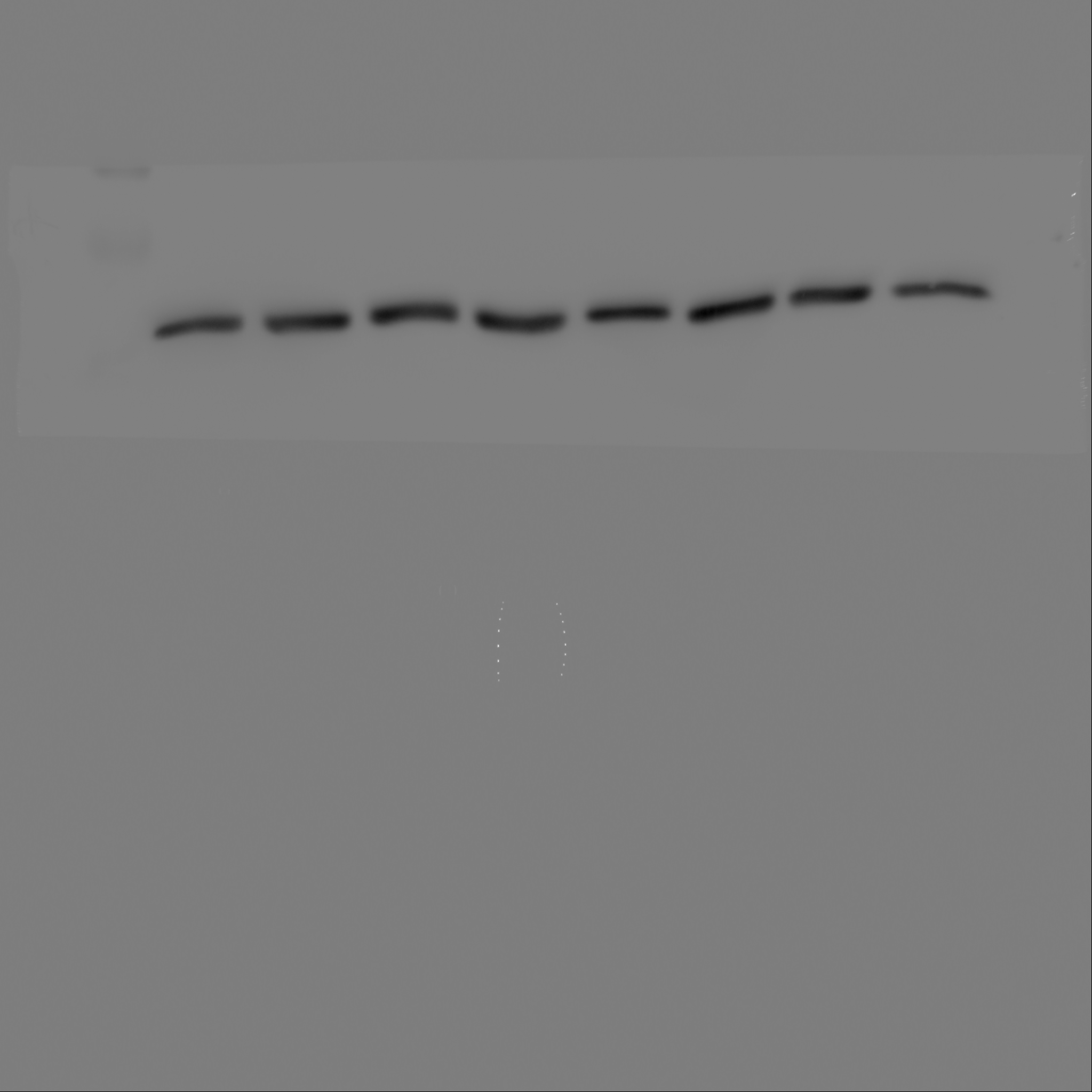

Supplement: FIGURES S1–S5 — File containing all the original uncropped western blot images depicted in the Figures 1(A,B), 2(A–E), 3(A,C–E), 4(A–E), and 5(B–E). [file Data_Sheet_1.ZIP › Figure 4 C/Tubulin/image.tif]

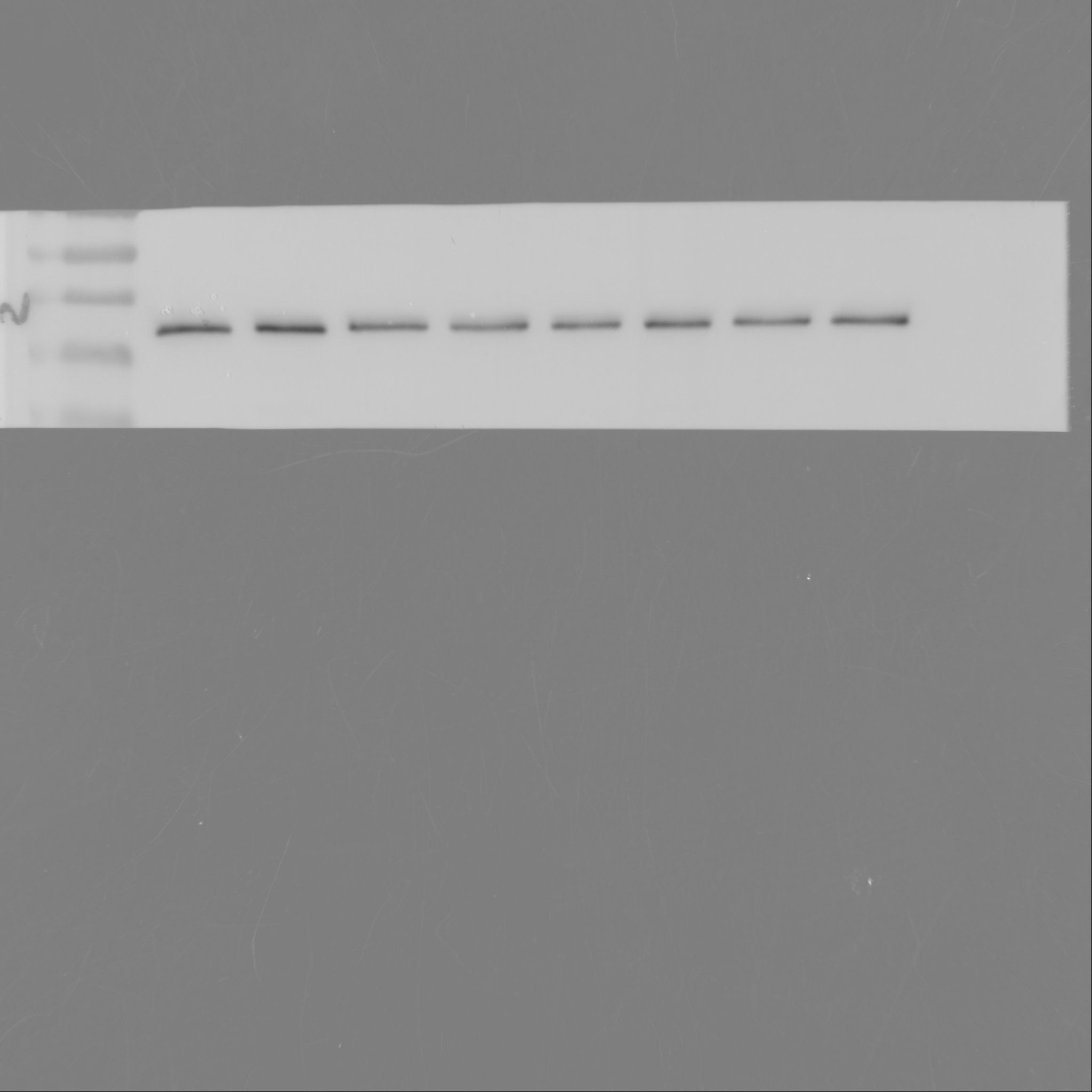

Supplement: FIGURES S1–S5 — File containing all the original uncropped western blot images depicted in the Figures 1(A,B), 2(A–E), 3(A,C–E), 4(A–E), and 5(B–E). [file Data_Sheet_1.ZIP › Figure 4 D/GAPDH/image.tif]

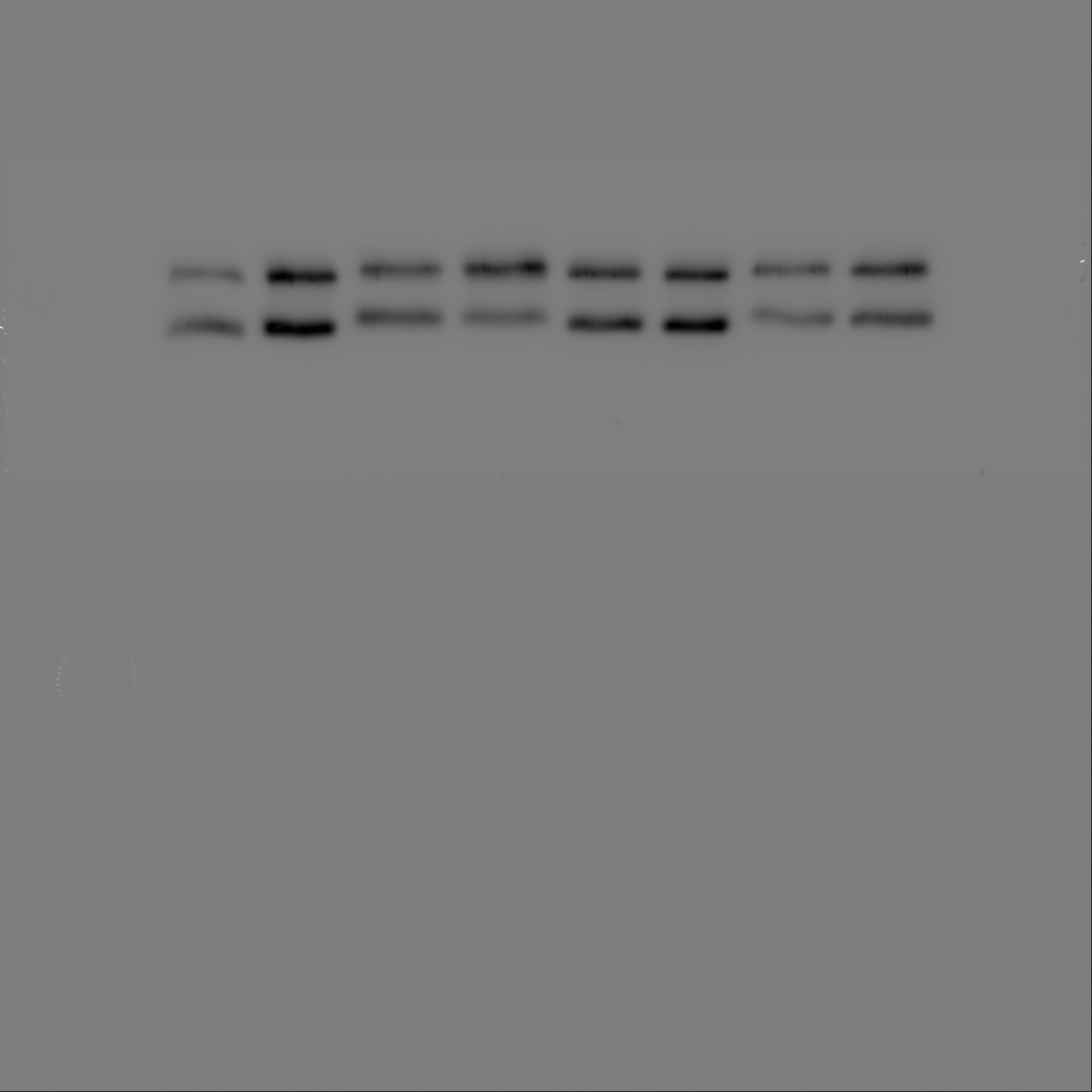

Supplement: FIGURES S1–S5 — File containing all the original uncropped western blot images depicted in the Figures 1(A,B), 2(A–E), 3(A,C–E), 4(A–E), and 5(B–E). [file Data_Sheet_1.ZIP › Figure 4 D/LC3/image.tif]

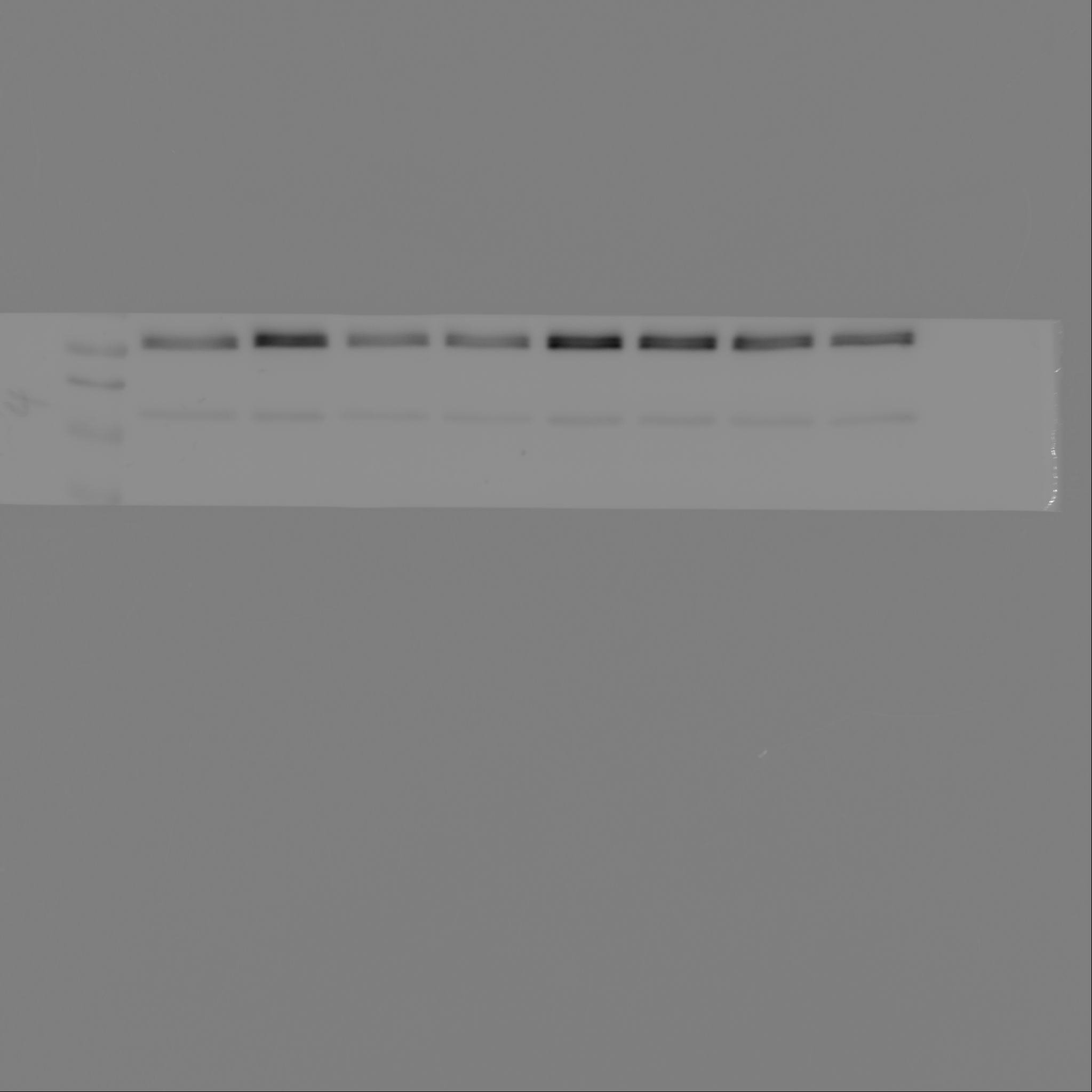

Supplement: FIGURES S1–S5 — File containing all the original uncropped western blot images depicted in the Figures 1(A,B), 2(A–E), 3(A,C–E), 4(A–E), and 5(B–E). [file Data_Sheet_1.ZIP › Figure 4 E/Beclin1/image.tif]

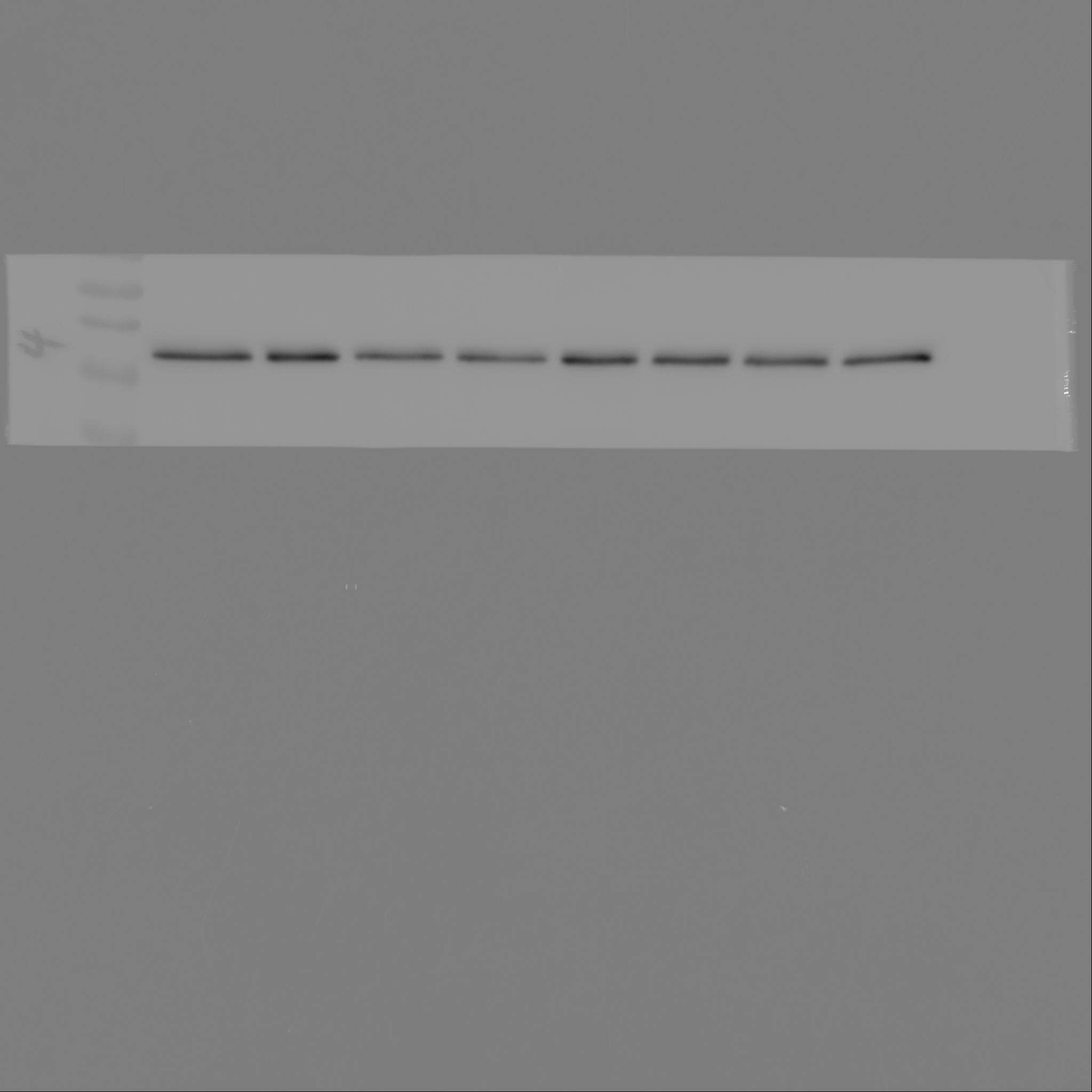

Supplement: FIGURES S1–S5 — File containing all the original uncropped western blot images depicted in the Figures 1(A,B), 2(A–E), 3(A,C–E), 4(A–E), and 5(B–E). [file Data_Sheet_1.ZIP › Figure 4 E/GAPDH/image.tif]

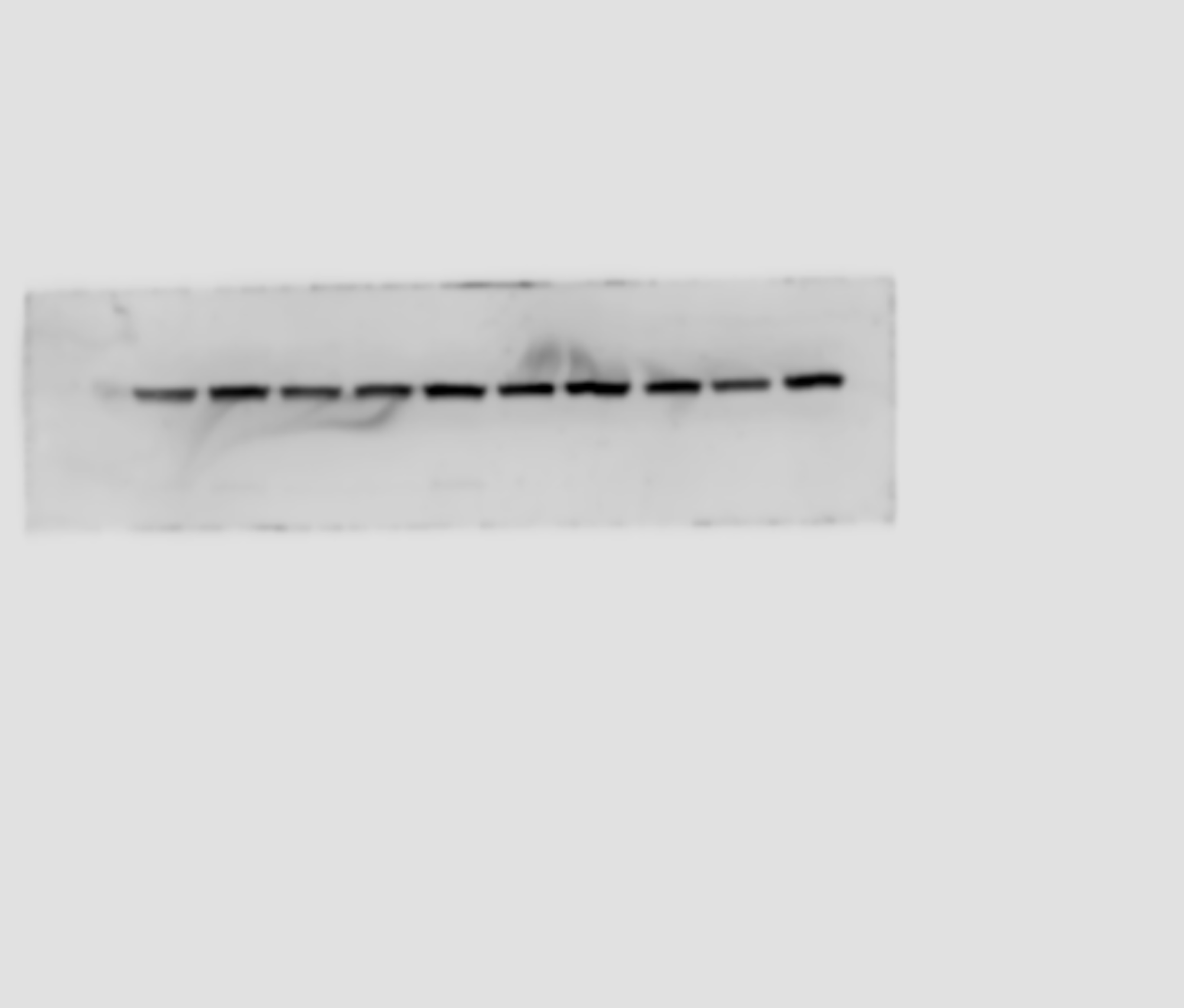

Supplement: FIGURES S1–S5 — File containing all the original uncropped western blot images depicted in the Figures 1(A,B), 2(A–E), 3(A,C–E), 4(A–E), and 5(B–E). [file Data_Sheet_1.ZIP › Figure 5 B/aSMA/Image_0000282_01.tif]

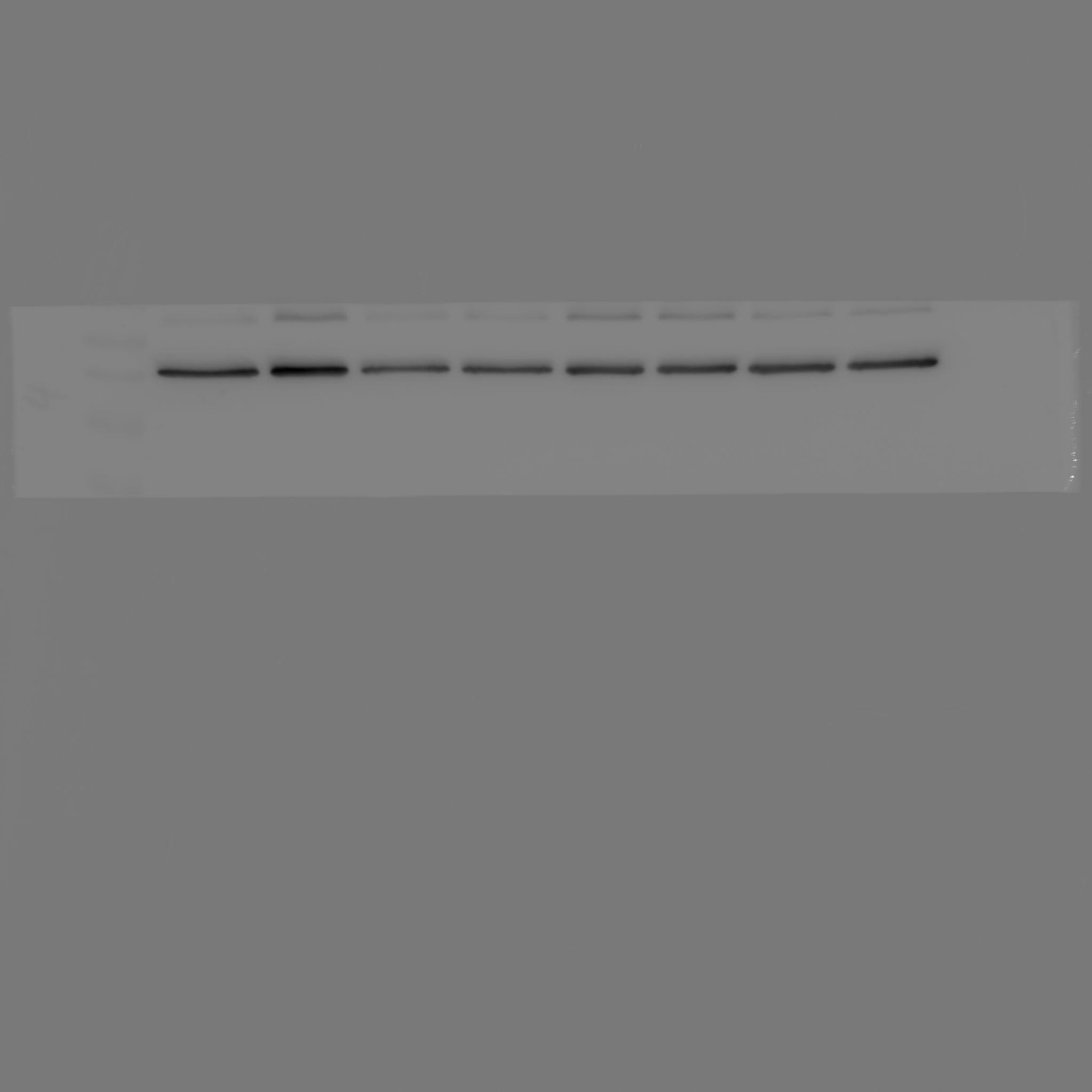

Supplement: FIGURES S1–S5 — File containing all the original uncropped western blot images depicted in the Figures 1(A,B), 2(A–E), 3(A,C–E), 4(A–E), and 5(B–E). [file Data_Sheet_1.ZIP › Figure 5 C/aSMA/image.tif]

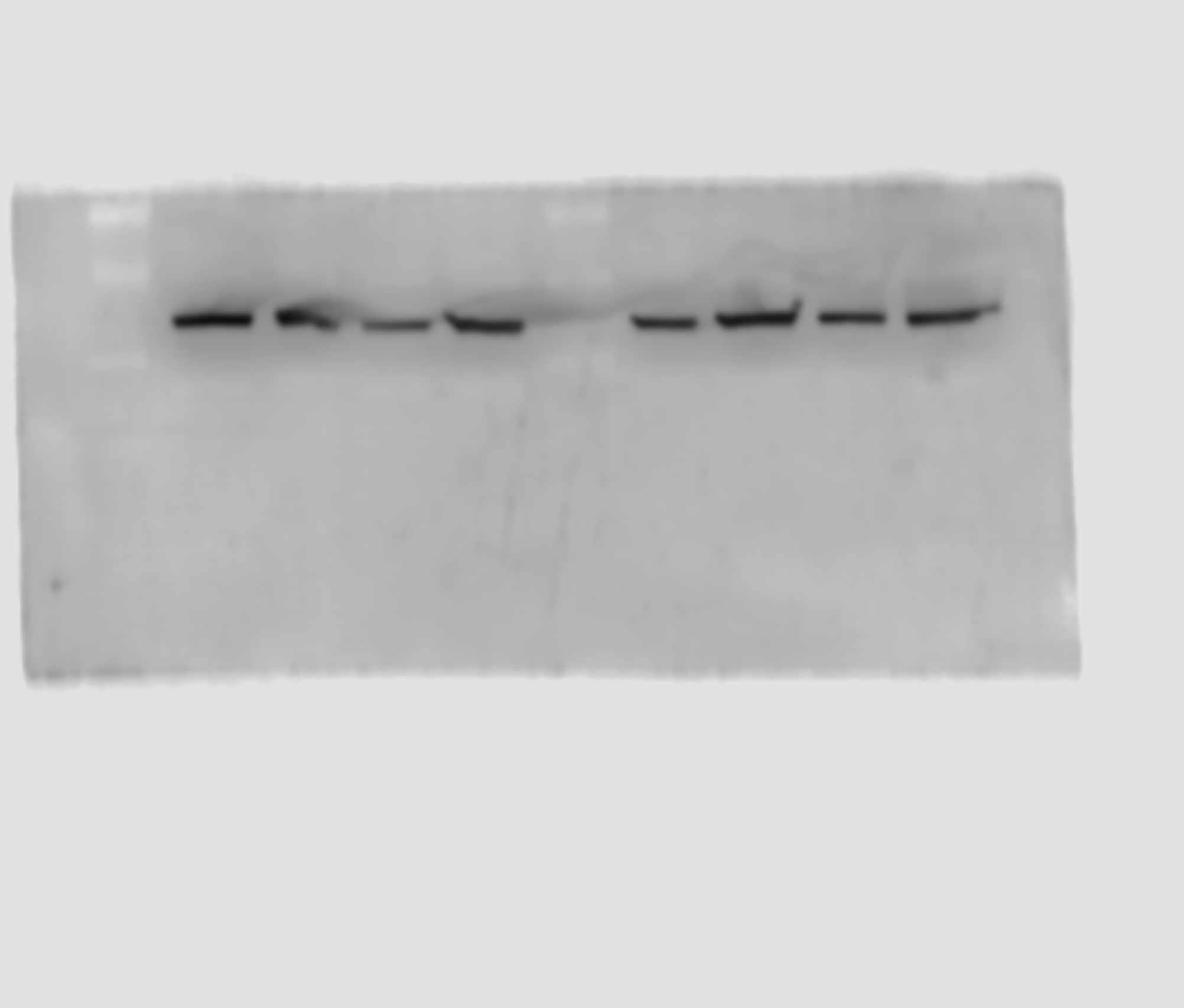

Supplement: FIGURES S1–S5 — File containing all the original uncropped western blot images depicted in the Figures 1(A,B), 2(A–E), 3(A,C–E), 4(A–E), and 5(B–E). [file Data_Sheet_1.ZIP › Figure 5 D/aSMA/Image_0000288_01.tif]

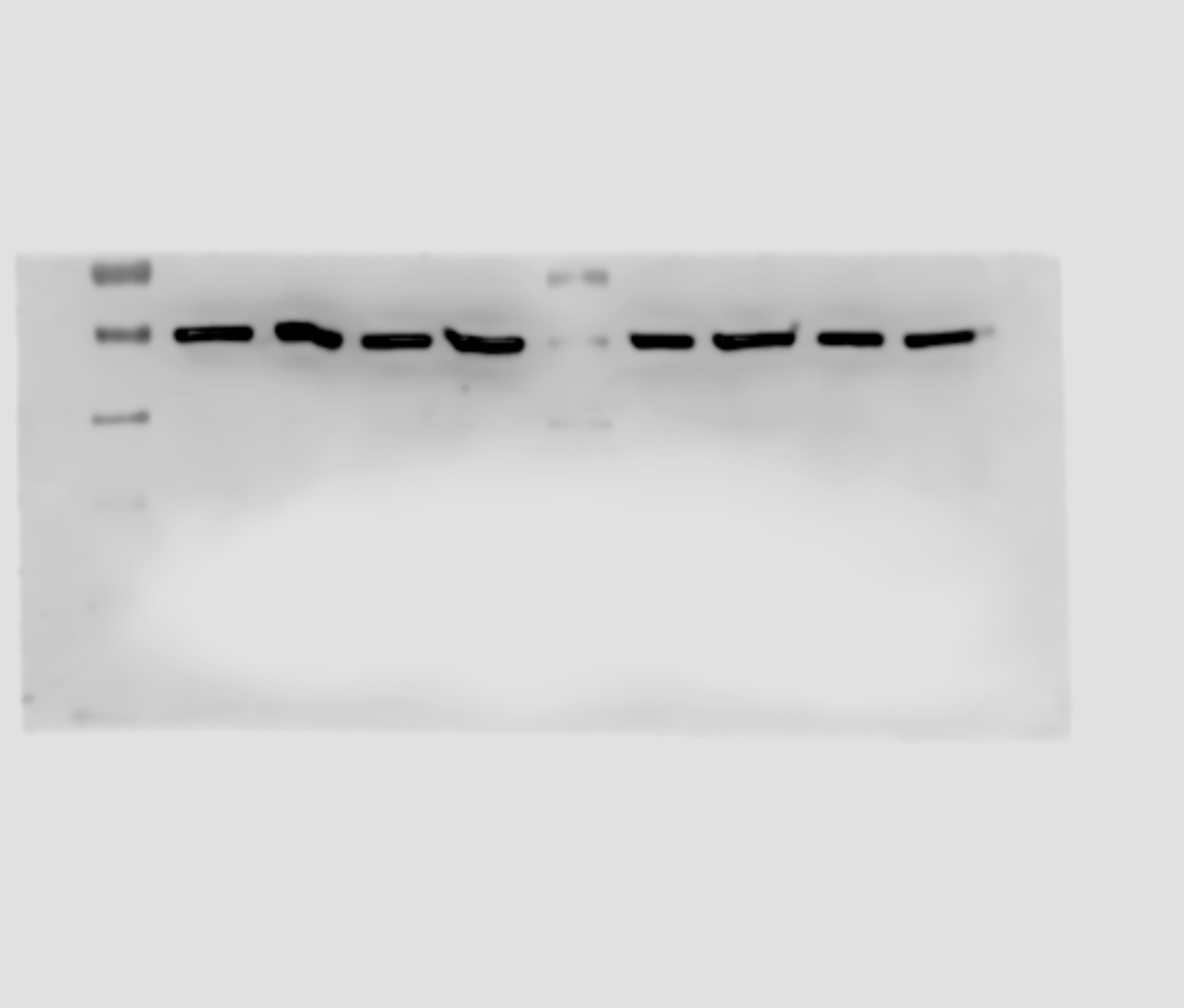

Supplement: FIGURES S1–S5 — File containing all the original uncropped western blot images depicted in the Figures 1(A,B), 2(A–E), 3(A,C–E), 4(A–E), and 5(B–E). [file Data_Sheet_1.ZIP › Figure 5 D/Tubulin/Image_0000184_01 1.tif]

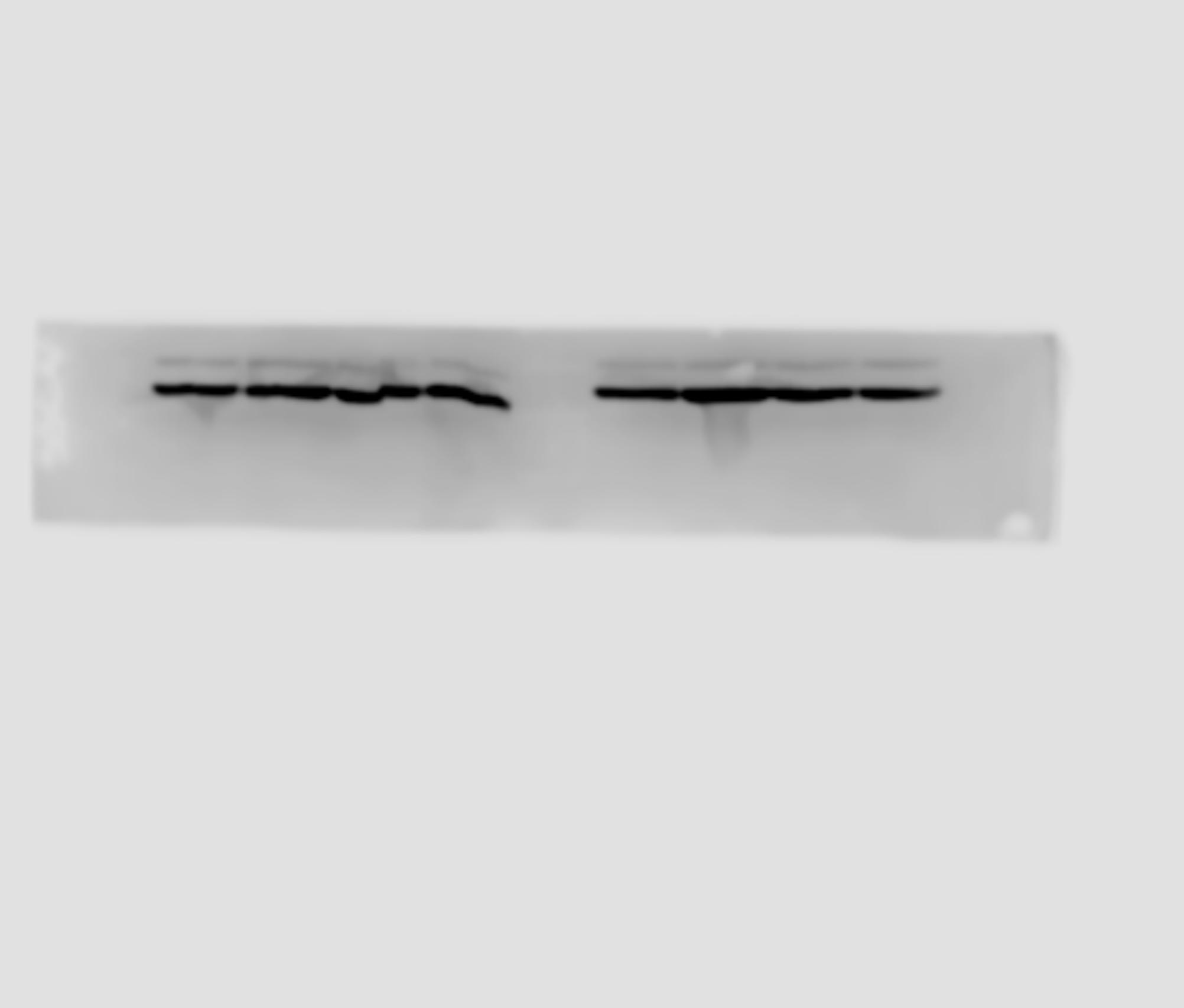

Supplement: FIGURES S1–S5 — File containing all the original uncropped western blot images depicted in the Figures 1(A,B), 2(A–E), 3(A,C–E), 4(A–E), and 5(B–E). [file Data_Sheet_1.ZIP › Figure 5 E/aSMA/Image_0000001_01.tif]
